# Supplementary material for: Between support and scepticism: Health professionals’ perceptions of a nutrition education program promoting low-carbohydrate, high-fat diets in under-resourced South African communities
Source: PLoS One. 2025 Jun 23;20(6):e0325179. doi: 10.1371/journal.pone.0325179 (PMC12184999; doi:10.1371/journal.pone.0325179)
Supplement: S1 File — (DOCX) [file pone.0325179.s001.docx]

**Supporting Information 1**

Table 1. Interview questions posed to participants.

| **In-depth questions for healthcare professionals** |
| --- |
| How would you describe your role as a health care provider in [community]? |
| What do you think are the major health problems/ diseases in [community]? |
| What are your thoughts about NCDs like obesity, diabetes, cardiovascular disease in [community]? |
| What lifestyle advice does the health care centre you work at offer to patients with chronic diseases, like obesity, diabetes and cardiovascular disease? |
| What role do you think nutrition plays in chronic diseases in [community]?   - Describe the foods/ nutrients/ diets you think lead to chronic diseases. - Describe the foods/ nutrients/ diets that you think should be used to prevent/ treat chronic disease in [community]. |
| How would you describe the diets of the patients that you see from [community]?   - Why do you think people from [community] eat this way? |
| Have you heard of the Eat Better South Africa nutrition education program?   - How and what did you hear about it? - If you had patients taking part in the EBSA program, how was their experience? |
| Could you describe the diet that you think EBSA recommends? |
| What do you think about EBSA holding a program in [community]? |
| How did you / would you respond if one of your patients told you they were following an EBSA/ Banting/ low carbohydrate high fat diet? |
| Have you heard about “Banting” or low-carbohydrate high-fat diets?   - Could you describe what a Banting or LCHF diet would look like? - What are your general impressions of this diet? - How do you think this diet relates to chronic diseases? Do you think it might make it better or worse? |
| How do you think nutrition can be improved for people living in [community]? |
