# Supplementary material for: Between support and scepticism: Health professionals’ perceptions of a nutrition education program promoting low-carbohydrate, high-fat diets in under-resourced South African communities
Source: PLoS One. 2025 Jun 23;20(6):e0325179. doi: 10.1371/journal.pone.0325179 (PMC12184999; doi:10.1371/journal.pone.0325179)
Supplement: Attachment — Submitted filename: PONE-D-24-55230 (1).pdf [file pone.0325179.s003.pdf]

PLOS ONE

Between support and skepticism: Health professionals' views on Low-Carbohydrate diets in under-resourced South African communities

--Manuscript Draft--

**Manuscript Number:**	PONE-D-24-55230
**Article Type:**	Research Article
**Full Title:**	Between support and skepticism: Health professionals' views on Low-Carbohydrate diets in under-resourced South African communities
**Short Title:**	Health professionals' views on Low-Carbohydrate diets in under-resourced South African communities
**Corresponding Author:**	Ahtisham Younas, Ph.D<br>Memorial University of Newfoundland<br>Saint John's, NL CANADA
**Keywords:**	thematic analysis; low-carbohydrate high-fat; South Africa; under-resourced communities; healthcare professionals
**Abstract:**	The burden of non-communicable diseases (NCDs) continues to rise, emphasizing the need for effective dietary interventions. Programs like Eat Better South Africa (EBSA) advocate for low-carbohydrate high-fat (LCHF) dietary choices, especially in disadvantaged communities. However, the adoption of such approaches among healthcare professionals remains contentious. This study explores healthcare professionals' perspectives on nutrition and the EBSA program, drawing on 16 in-depth individual interviews with physicians, nurses, and dietitians from False Bay Hospital, Groote Schuur Hospital, and a primary health clinic in Hout Bay, Western Cape, South Africa. Thematic analysis revealed four main findings. Healthcare professionals lacked confidence in their nutritional knowledge, and while many were familiar with the LCHF diet, opinions varied regarding its sustainability and health implications. Concerns were raised about the high fat content and affordability of LCHF foods. Professionals acknowledged the value of group support in behavior change, as promoted by EBSA, but expressed reservations about its strong emphasis on LCHF diets. Key challenges identified for patients included poverty, cultural beliefs, limited education, and access to nutritious foods. The findings highlight a reliance on traditional dietary advice, with uncertainties about the feasibility and affordability of LCHF diets, potentially conflicting with programs like EBSA. This study contributes novel insights into healthcare professionals' perspectives on LCHF diets and community-based interventions in South Africa, reflecting both cautious endorsement and critical concerns about their implications.
**Order of Authors:**	Georgina Pujol-Busquets
	Kate Larmuth
	Christopher C Webster
	James Smith
	Ahtisham Younas, Ph.D
	Sergi Fàbregues
	Anna Bach-Faig
**Opposed Reviewers:**	
**Additional Information:**	
**Question**	**Response**
**Financial Disclosure**<br><br>Enter a financial disclosure statement that describes the sources of funding for the	The author(s) received no specific funding for this work.

Powered by Editorial Manager® and ProduXion Manager® from Aries Systems Corporation

work included in this submission. Review the submission guidelines for detailed requirements. View published research articles from *PLOS ONE* for specific examples.<br><br>This statement is required for submission and **will appear in the published article** if the submission is accepted. Please make sure it is accurate.<br><br>**Funded studies**<br>Enter a statement with the following details:<br>• Initials of the authors who received each award<br>• Grant numbers awarded to each author<br>• The full name of each funder<br>• URL of each funder website<br>• Did the sponsors or funders play any role in the study design, data collection and analysis, decision to publish, or preparation of the manuscript?<br><br>Did you receive funding for this work?	
**Competing Interests**<br><br>Use the instructions below to enter a competing interest statement for this submission. On behalf of all authors, disclose any competing interests that could be perceived to bias this work—acknowledging all financial support and any other relevant financial or non-financial competing interests.<br><br>This statement is required for submission and **will appear in the published article** if the submission is accepted. Please make sure it is accurate and that any funding sources listed in your Funding Information later in the submission form are also declared in your Financial Disclosure statement.<br><br>View published research articles from *PLOS ONE* for specific examples.	The authors have declared that no competing interests exist.

**NO authors have competing interests**<br><br>Enter: *The authors have declared that no competing interests exist.*<br><br>**Authors with competing interests**<br><br>Enter competing interest details beginning with this statement:<br><br>*I have read the journal's policy and the authors of this manuscript have the following competing interests: [insert competing interests here]*<br><br>* typeset	
**Ethics Statement**<br><br>Enter an ethics statement for this submission. This statement is required if the study involved:<br><br>• Human participants<br>• Human specimens or tissue<br>• Vertebrate animals or cephalopods<br>• Vertebrate embryos or tissues<br>• Field research<br><br>Write "N/A" if the submission does not require an ethics statement.<br><br>General guidance is provided below. Consult the submission guidelines for detailed instructions. **Make sure that all information entered here is included in the Methods section of the manuscript.**	The Human Research Ethics Committee (HREC REF 512/2019) of the University of Cape Town approved the study protocol and informed consent procedures. Written informed consent was obtained from all participants

Powered by Editorial Manager® and ProduXion Manager® from Aries Systems Corporation

**Format for specific study types**<br><br>**Human Subject Research (involving human participants and/or tissue)**<br>• Give the name of the institutional review board or ethics committee that approved the study<br>• Include the approval number and/or a statement indicating approval of this research<br>• Indicate the form of consent obtained (written/oral) or the reason that consent was not obtained (e.g. the data were analyzed anonymously)<br><br>**Animal Research (involving vertebrate animals, embryos or tissues)**<br>• Provide the name of the Institutional Animal Care and Use Committee (IACUC) or other relevant ethics board that reviewed the study protocol, and indicate whether they approved this research or granted a formal waiver of ethical approval<br>• Include an approval number if one was obtained<br>• If the study involved *non-human primates*, add *additional details* about animal welfare and steps taken to ameliorate suffering<br>• If anesthesia, euthanasia, or any kind of animal sacrifice is part of the study, include briefly which substances and/or methods were applied<br><br>**Field Research**<br><br>Include the following details if this study involves the collection of plant, animal, or other materials from a natural setting:<br>• Field permit number<br>• Name of the institution or relevant body that granted permission	
**Data Availability**<br><br>Authors are required to make all data underlying the findings described fully available, without restriction, and from the time of publication. PLOS allows rare exceptions to address legal and ethical concerns. See the PLOS Data Policy and FAQ for detailed information.	No - some restrictions will apply

A Data Availability Statement describing where the data can be found is required at submission. Your answers to this question constitute the Data Availability Statement and **will be published in the article**, if accepted.<br><br>**Important:** Stating 'data available on request from the author' is not sufficient. If your data are only available upon request, select 'No' for the first question and explain your exceptional situation in the text box.<br><br>Do the authors confirm that all data underlying the findings described in their manuscript are fully available without restriction?	
**Describe where the data may be found in full sentences. If you are copying our sample text, replace any instances of XXX with the appropriate details.**<br><br>• If the data are **held or will be held in a public repository**, include URLs, accession numbers or DOIs. If this information will only be available after acceptance, indicate this by ticking the box below. For example: *All XXX files are available from the XXX database (accession number(s) XXX, XXX.).*<br>• If the data are all contained **within the manuscript and/or Supporting Information files**, enter the following: *All relevant data are within the manuscript and its Supporting Information files.*<br>• If neither of these applies but you are able to provide **details of access elsewhere**, with or without limitations, please do so. For example:<br><br>*Data cannot be shared publicly because of [XXX]. Data are available from the XXX Institutional Data Access / Ethics Committee (contact via XXX) for researchers who meet the criteria for access to confidential data.*<br><br>*The data underlying the results presented in the study are available from (include the name of the third party*	Data cannot be shared publicly because of ethical restrictions. Data are available from the first author Georgina Pujol-Busquets on reasonable request.

and contact information or URL).<br>• This text is appropriate if the data are owned by a third party and authors do not have permission to share the data.<br><br>* typeset	
Additional data availability information:	

AHTISHAM YOUNAS
Faculty of Nursing
Memorial University of Newfoundland
St. John's, NL A1C 5S7
P.O. Box 4200
CANADA

Dear PLOS One Editors,

On behalf of my coauthors, I am submitting a manuscript entitled “Between support and skepticism: Health professionals’ views on Low-Carbohydrate diets in under-resourced South African communities" for consideration as a research article in *PLOS One*.

This study explores healthcare professionals’ perspectives on the implementation of Low-Carbohydrate, High-Fat (LCHF) dietary interventions in under-resourced South African communities, focusing on the Eat Better South Africa (EBSA) program. To our knowledge, this is the first qualitative study examining healthcare practitioners’ views on LCHF diets and related community-based initiatives in South Africa. Our findings provide critical insights into the alignment and discord between healthcare professionals’ dietary recommendations and emerging community-based nutrition interventions, with implications for addressing non-communicable diseases (NCDs) in low-income settings.

This qualitative study is part of Dr Georgina Pujol-Busquets’s PhD dissertation, which encompasses four research studies. The thesis is available in the open-access institutional repository of the University of Cape Town (UCT) under the title, “Evaluation of a nutrition and health education program on components of metabolic health in women from under-resourced South African communities.” The following three studies from this project have already been published elsewhere and provide valuable insights into the topic under investigation:

1. Pujol-Busquets G, Smith J, Larmuth K, Fàbregues S, Bach-Faig Exploring the perceptions of women from under-resourced South African communities about participating in a low-carbohydrate high-fat nutrition and health education program: A qualitative focus group study. *Nutrients*. 2020; 12(4):894. https://doi.org/10.3390/nu12040894
2. Pujol-Busquets G, Smith J, Fàbregues S, Bach-Faig A, Larmuth K. Community assessment for a low-carbohydrate nutrition education program in South Africa. *Nutrients*. 2023; 15(1):67. https://doi.org/10.3390/nu15010067
3. Pujol-Busquets G, Smith J, Fàbregues S, Bach-Faig A, Larmuth K. Mixed methods evaluation of a low-carbohydrate high-fat nutrition education program for women from underserved communities in South Africa. *Appetite*. 2024;204:107725. https://10.1016/j.appet.2024.107725

This study builds on previous research on LCHF dietary interventions, particularly in resource-constrained environments, but adds a novel perspective by examining the views of healthcare practitioners. This work contributes to the broader literature on nutrition education, health equity, and the barriers to adopting non-traditional dietary approaches in disadvantaged communities.

We confirm that this manuscript has not been previously published and is not under consideration elsewhere. We have had no prior interactions with *PLOS One* regarding this manuscript. I hereby confirm that all authors listed on the manuscript accept full responsibility for all aspects of the work described.

Thank you for considering this submission. We believe our study aligns with the scope and mission of PLOS One, and we look forward to the possibility of contributing to your esteemed journal.

Yours sincerely,
Ahtisham Younas, PhD

Manuscript

Click here to access/download;Manuscript;Manuscript_Plosone_Updated.do

Between support and skepticism: Health professionals' views on ~~Low-Carbohydrate diets~~ in under-resourced South African communities

SHORT TITLE: Health professionals views on Low-Carbohydrate diets

Georgina Pujol-Busquets[1,2,], Kate Larmuth[1,3,4], Christopher C Webster [1,3,4], James Smith [1,3,4], Ahtisham Younas[5*], Sergi Fàbregues[6] and Anna Bach-Faig[7]

[1]Division of Physiological Sciences, Department of Human Biology, University of Cape Town, 7700, South Africa Health through Physical Activity Lifestyle and Sport Research Centre, Sports Science Institute of South Africa; University of Cape Town, 7700, South Africa

[2]Faculty of Health Sciences, Universitat Oberta de Catalunya (Open University of Catalonia, UOC), 08018 Barcelona, Spain

[3]International Federation of Sports Medicine (FIMS) Collaborative Centre of Sports Medicine, HPALS, University of Cape Town, 7700, South Africa

[4]Research Center for Health through Physical Activity, Lifestyle and Sport, Division of Physiology, Department of Human Biology, Faculty of Health Sciences, University of Cape Town, Cape Town 7700, South Africa

[5]Faculty of Nursing, Memorial University of Newfoundland, St. John's, NL, Canada.

[6]Department of Psychology and Education, Universitat Oberta de Catalunya (UOC), 08018 Barcelona, Spain

[7]FoodLab Research Group (2021 SGR 01357), Faculty of Health Sciences, Universitat Oberta de Catalunya (Open University of Catalonia, UOC), 08018 Barcelona, Spain

*Corresponding author: Ahtisham Younas, Memorial University, Office H2930, 300 Prince Phillip Drive, St. John's, Newfoundland A1B 3V6, Canada. Email: ay6133@mun.ca

Abstract

The burden of non-communicable diseases (NCDs) continues to rise, emphasizing the need for effective dietary interventions. Programs like Eat Better South Africa (EBSA) advocate for low-carbohydrate high-fat (LCHF) dietary choices, especially in disadvantaged communities. However, the adoption of such approaches among healthcare professionals remains contentious. This study explores healthcare professionals' perspectives on nutrition and the EBSA program, drawing on 16 in-depth individual interviews with physicians, nurses, and dietitians from False Bay Hospital, Groote Schuur Hospital, and a primary health clinic in Hout Bay, Western Cape, South Africa. Thematic analysis revealed four main findings. Healthcare professionals lacked confidence in their nutritional knowledge, and while many were familiar with the LCHF diet, opinions varied regarding its sustainability and health implications. Concerns were raised about the high fat content and affordability of LCHF foods. Professionals acknowledged the value of group support in behavior change, as promoted by EBSA, but expressed reservations about its strong emphasis on LCHF diets. Key challenges identified for patients included poverty, cultural beliefs, limited education, and access to nutritious foods. The findings highlight a reliance on traditional dietary advice, with uncertainties about the feasibility and affordability of LCHF diets, potentially conflicting with programs like EBSA. This study contributes novel insights into healthcare professionals' perspectives on LCHF diets and community-based interventions in South Africa, reflecting both cautious endorsement and critical concerns about their implications.

Keywords

thematic analysis; low-carbohydrate high-fat; South Africa; under-resourced communities; healthcare professionals.

Introduction

In South Africa, all citizens, including undocumented immigrants, are constitutionally entitled to healthcare services, available through both public facilities and private sectors, often requiring payment. The District Health System, a central part of the public sector, is the primary access point for many [1]. However, public health institutions face significant challenges, including long wait times, poor hygiene, inadequate infection control, preventable errors leading to litigation, medicine and equipment shortages, and poor recordkeeping [2-4]. These issues stem from unequal resource distribution, rapid urbanization, rising disease rates, and slow healthcare system restructuring [5-7]. Additionally, a shortage of healthcare professionals, exacerbated by inadequate training, recruitment, retention, and mismanagement, further strains the system [8,9].

The healthcare workforce is crucial to advancing universal healthcare goals, particularly in addressing South Africa's complex disease challenges, both infectious and non-communicable [2,9-12]. Despite nutrition's significant impact on health, healthcare training programs often lack comprehensive integration of nutritional science, leading to conflicting advice and insufficient training to address nutrition-related conditions effectively [13-16]. This is a global concern, as nutrition profoundly influences health outcomes [17-20]. International efforts are bridging gaps in nutrition education, supported by evidence of the effectiveness of dietary and physical activity interventions, which often rival pharmacological treatments in efficacy, safety, and cost-effectiveness [13,16, 21- 24]. Studies consistently show that nutrition interventions reduce the incidence and severity of diet-related non-communicable disease (NCDs), improve patient outcomes, reduce healthcare costs, and enhance the well-being of healthcare professionals, especially in acute care settings [25–30].

Various nutritional education initiatives are active in low socio-economic areas of South Africa to address the burden of NCDs, with Eat Better South Africa (EBSA) being one

prominent program [31]. EBSA aims to empower individuals, particularly women, through dietary education, believing in the transformative power of education on health behaviours and overall well-being [31]. EBSA advocates for low-carbohydrate high-fat (LCHF) diets, which some clinicians and professional societies support as an effective treatment for diabetes and obesity management [31–33]. However, this approach is contentious, with conflicting findings polarizing expert opinion and potentially causing confusion among healthcare professionals and patients [34]. Despite controversies, carbohydrate restriction for managing type 2 diabetes is supported by international consensus guidelines [35,36]. Although this concept is not new, dating back to as early as 1869 with William Banting, even then Banting acknowledged and anticipated controversy and reputational risks associated with this diet [37].

In focus group discussions involving previous participants of EBSA programs, participants noted differences between the dietary guidance provided by healthcare professionals, which aligns with a Western Standard diet, in their communities and the EBSA recommendations [38-40]. Some healthcare professionals initially skeptical of the EBSA LCHF diet have shifted towards supporting it, as they witnessed positive health outcomes in their patients. EBSA advocates for a lower-carbohydrate, higher-healthy-fat diet, aligning with many current dietary recommendations. While some health professionals may support LCHF diets for certain individuals or specific health conditions, others may have reservations based on concerns about long-term health effects, individual variability, sustainability, adherence, and potential nutrient deficiencies [41,42]. Understanding healthcare professionals' perceptions of the EBSA program, LCHF diets and community nutrition practices is crucial for program improvement. This article aims to explore healthcare professionals' perspectives on the EBSA program, particularly focusing on the effectiveness and sustainability of the LCHF diet in managing NCDs within South Africa. It examines the alignment and potential conflicts between the nutritional advice given by professionals and the guidelines promoted by EBSA,

identifying gaps in nutrition education and training. The article also explores the broader public health implications of integrating such dietary recommendations, and seeks to provide insights for refining the EBSA program to better meet the health needs of South Africa's diverse population.

Materials and Methods

This is a qualitative study using in-depth individual interviews (IDIs) with health professionals from health care facilities that would service the current and future EBSA programs. They were selected from two tertiary institutions, namely False Bay Hospital and Groote Schuur Hospital, and a primary health clinic in Hout Bay. False Bay Hospital is a public hospital that was opened in 1965 in Fish Hoek, a town approximately 30 kilometres south of Cape Town. The hospital has approximately 80 beds and its facilities include a 24-Hour Emergency Unit. It is the only district hospital in the South Peninsula Health District of the Metro Region and serves many communities, including Simonstown, Masiphumelele, Ocean View, Clovelly, and many more surrounding areas. Groote Schuur Hospital is one of Cape Town's largest government-funded academic hospitals and was officially opened in 1938. The hospital has almost 900 beds and it is renowned as the hospital where the world's first human-to-human heart transplant was performed in 1967 by Christiaan Barnard and his team [43,44]. The Hout Bay health clinic non-profit organization works mostly in the informal settlement called Imizamo Yethu and provides nutrition and health education to the community. Hout Bay is a seaside town in the Western Cape province situated in the Cape Peninsula, 20 kilometres south of Cape Town.

Participants

To be eligible for the study, participants had to be health professionals; work in and see patients from the communities where EBSA has conducted a nutrition education program or a community where EBSA is planning to run future programs; and be able to understand and

speak English. Community health clinics were contacted by email or phone calls to identify potential participants. A researcher explained the study to interested participants, either via phone or at a scheduled meeting, and, if they met the inclusion criteria, they were provided with a study information sheet and the informed consent form. An interview visit was then scheduled at a convenient time and place for participants. Participants met individually with a researcher for about an hour in a private venue, where they signed the informed consent and were interviewed in English.

Procedures

The IDIs were carried out between November 29, 2019 and February 21, 2020 and lasted an average of 30 minutes. The sample size is consistent with recommendations in the literature which ranges between 5 to 50 participants [45,46]. According to the literature, the first five to six IDIs generally produce the majority of the new data, and the majority of concepts are identified within the first 10 interviews. As such, new participants were interviewed until saturation was reached [45,46]. Participants were interviewed on a one-on-one basis to explore their understanding and perceptions of chronic disease, nutrition, low-carbohydrate high-fat diets, and the EBSA program. A semi structured interview guide (Table 1) was used which included both open-ended and probing questions as a guide for the discussion, although questions were not necessarily asked verbatim. Probing questions were asked only if subjects had not already been covered. All interviews were audio-recorded, with the permission of the participants. Interviews were conducted by GPB and saved in a password-protected folder. Recordings were transcribed by Cyber Transcription (South Africa) in the original language. The accuracy and editing of the transcriptions were double-checked by an external qualitative researcher for rigor and appropriateness. Each transcribed interview was compared with the corresponding audio, to ensure that the transcription had been done in context and captured the interview as accurately as possible.

Table 1. Interview questions posed to participant.

**In-depth questions for healthcare professionals**
How would you describe your role as a health care provider in [community]?
What do you think are the major health problems/ diseases in [community]?
What are your thoughts about NCDs like obesity, diabetes, cardiovascular disease in [community]?
What lifestyle advice does the health care centre you work at offer to patients with chronic diseases, like obesity, diabetes and cardiovascular disease?
What role do you think nutrition plays in chronic diseases in [community]?<br>• Describe the foods/ nutrients/ diets you think lead to chronic diseases.<br>• Describe the foods/ nutrients/ diets that you think should be used to prevent/ treat chronic disease in [community].
How would you describe the diets of the patients that you see from [community]?<br>• Why do you think people from [community] eat this way?
Have you heard of the Eat Better South Africa nutrition education program?<br>• How and what did you hear about it?<br>• If you had patients taking part in the EBSA program, how was their experience?
Could you describe the diet that you think EBSA recommends?
What do you think about EBSA holding a program in [community]?
How did you / would you respond if one of your patients told you they were following an EBSA/ Banting/ low carbohydrate high fat diet?
Have you heard about "Banting" or low-carbohydrate high-fat diets?<br>• Could you describe what a Banting or LCHF diet would look like?<br>• What are your general impressions of this diet?

• How do you think this diet relates to chronic diseases? Do you think it might make it better or worse?
How do you think nutrition can be improved for people living in [community]?

Data analysis

Braun and Clarke's approach to codebook type thematic analysis [47] was used to identify and analyse themes with NVivo 12 software (QSR Internationality Ltd., Melbourne, Australia). All the researchers participated in the analysis, which was conducted in a stepwise fashion. First, CCW and GPB read and summarized the transcripts. Second, two respective codebooks were developed inductively by CCW and GPB, based on the previous readings. Third, CCW and GPB discussed their individual codebooks and merged them into one codebook. Finally, the two researchers independently coded three randomly selected interviews using separate NVivo projects. Thereafter, CCW and GPB met again to revise the coding, and disagreements were discussed and resolved by consensus. The content and structure of the final codebook was subsequently finalised, with consensus from ABF, SF, and KL to check for consistency in the codes. Thereafter, the interviews were divided into two groups, each containing eight interviews, and one group each were assigned to GBP and CCW. Both coding schemes emerged in the same NVivo project, and GPB and CCW worked on developing the themes that were, subsequently, displayed by GPB as thematic maps.

The following step involved naming the themes and defining the main concepts of each theme, resulting in a total of four themes. The last phase, which was mainly conducted by GPB, consisted of producing a report. The resulting themes were shared with ABF, SF, KL and JS to confirm their relevance to the research questions. Although GPB conducted the interviews and participated in the analysis, multiple coders (CCW and GPB) were used to allow greater probability to ensure that all themes were identified, and that data were being consistently

interpreted to limit the potential effect of bias or singular point of view. Quotations from the qualitative data were selected to illustrate the emergent themes, and these capture the essence of the theme. Square brackets indicate text added by the authors to provide clarity or context and an ellipsis indicates the omission of irrelevant parts of the quote. The health professionals' professions are included with the participant quotes with the gender provided in parentheses at the end of the quote.

Ethical considerations

The Human Research Ethics Committee (HREC REF 512/2019) of the University of Cape Town approved the study protocol and informed consent procedures. All procedures performed were in accordance with the 1964 Helsinki Declaration and its later amendments. Written Informed consent was obtained from all individual participants involved in the study. The health facilities of the study consented to the interviews being conducted at their practices.

Results

Participants recruited for the study were from a variety of general practices and socio-economic localities. Sixteen participants completed the study, eleven women and five men; seven were medical physicians, seven were nurses and two were dietitians. The average age of participants was 37.1 ± 9.8 years. A majority of participants (n = 13) worked only in the public sector, two participants practiced in both the public and private sector and one participant practiced only in the private sector. Four themes (Table 2) were developed from the analysis.

Table 2. Themes developed from IDIs with health professionals.

Theme	Definition	Quotes
**Theme 1.**	Refers to their understanding of dietary principles,	*'The reason why I'm saying I'm confused is I remember 4 or 5 years ago there was a huge promotion of [foods containing] poly-unsaturated fatty acids. Two years ago, poly-unsaturated*

**Foundations of dietary understanding**	nutritional requirements, and the role of food in promoting health and preventing disease among the population. This knowledge encompasses various aspects including nutritional guidelines, assessment, dietary counselling and nutrition education.	*fatty acids were hailed as causing lots of cancer. So now, you're uncertain which to promote' (Male physician).*<br>*'Studies have shown that with diabetes even if you increase the medication or you optimize the medication, at the end of the day it's the diet that kind of makes or breaks the disease' (Female physician).*<br>*'We basically follow the protocols of the National Health Department and at the moment, there isn't really much with regards to prevention, they have information, but it's outdated and it's not up to speed with what's really happening' (Male physician).*<br>*'I definitely don't say you need to cut it out completely [sugar]. Because I don't think it is realistic. I understand we all do enjoy sweet things here and there' (Female dietitian).*
**Theme 2. LCHF: Controversies and beliefs**	Understanding of health professionals around the recommendations of LCHF diets which depend on their training, experience and personal beliefs.	*'I was on [a LCHF diet], but because I didn't have a lot of support (...) I just left it then, but it's actually a good diet, I found out, and I didn't feel so tired' (Female nurse).*<br>*'If you eat high fat things your cholesterol is going to go up (...) Our nutritional education is lacking because you're not taught about the good types of fats and the bad types of fats' (Female physician).*<br>*'I haven't read all the research about it [LCHF diet]. I know it's very controversial. I know that it's not completely accepted*

		(...) I think it's almost too much of a mindset change for most people' (Male physician).<br>*'I don't advocate a LCHF diet but I'm supportive of what my patients want to try and then I monitor them closely. I watch their cholesterols, I watch their triglycerides, I watch their blood pressures.' (Male physician).*<br>*'I wouldn't encourage it [LCHF diet], but if a patient really wanted to do it and ask for my advice, I would try to guide them on it yes, for sure' (Female dietitian).*
**Theme 3. EBSA's role in community nutrition**	Health professionals' understanding and perceptions of the EBSA program promoting LCHF within under-resourced communities which encompass EBSA's group support approach, diet recommendations and program's sustainability.	*'When you're in a group, then there's more support from one another. Then you don't feel like, no, I want to eat the chocolate, but now that support comes in' (Female nurse).*<br>*'I think it's a good thing, especially to educate yourself' (Female nurse).*<br>*'I think it's a great initiative, very needed, very necessary because it will probably, if it's successful, it will definitely reduce the disease burden in our community and that impacts on everything' (Female physician).*<br>*'If the people don't have the funding, they're not going to be able to buy it [EBSA's recommended food]' (Female physician).*

**Theme 4: Understanding socioeconomic determinants of health**	Health professionals' understanding of the barriers that under-resourced patients face in accessing healthcare and maintaining good health. Their views on these barriers are informed by their direct interactions with patients, as well as their broader understanding of social determinants of health.	*'When you're in that state of poverty with 3 to 4 children per household may be 50% of adults unemployed, only 20% of people are living in fixed accommodation as opposed to like" shacks". You kind of get to that expression that beggars can't be choosers, and so while they might know that what they're eating is not good for them, they don't have the means to put that into place' (Male physician).*<br>*'I think education is the most important thing. Most patients don't know that sweets are bad' (Female dietitian).*<br>*'It's a very big cultural thing to have, to be big [body size], so it's another thing, there's a stigma around it if you're skinny and you're losing weight, you are becoming sick' (Female physician).*<br>*'I think in certain cultures, women don't have a lot of say in what gets done, so if the man comes home and he says "he wants this sort of meal" that's what she must prepare. And a lot of the women also aren't educated about contraception, so they end up having lots of children and it's difficult to feed so many mouths with this expensive [LCHF] diet you know' (Female physician).*<br>*'Some people get addicted to cigarettes, and I wonder if some people get addicted to certain types of food, high fat food from outlet stores or a high carbohydrate diet. I wonder if that stimulates the same sort of mechanism, the same kind of pathway' (Male physician).*

LCHF: Low carbohydrate high fat; EBSA: Eat Better South Africa.

Theme 1: Foundations of dietary understanding

The health professionals recognised that they are not that well-informed regarding nutritional advice and its role in disease prevention. Professionals felt that they barely have enough time to see patients for clinical advice and feel that nutritional advice, although important for disease prevention, requires more time. They did, however, acknowledge that nutritional advice is ‘vital’ to promote healthy-diet behaviours so that diet-related chronic diseases can be addressed. There seems to be general confusion regarding nutrition in this sample as they mentioned that every second article one reads contradicts the previous one (male physician), there is not sufficient data, and they do not keep up with new studies. As a result, professionals find it difficult to deliver nutritional advice. As it stands, they tend to use many ‘catchphrases’ and their own statements of beliefs. This is effectively based on what has traditionally been promoted as healthy behaviour: small portions, reduce red meat, eat fruit and vegetables, drink water, reduce dietary fats, avoid unsaturated fats (for instance, remove fat and skin from chicken) and practise physical activity. Some of them produced unsupported statements to explain this advice, and they repeated those statements to their patients.

Nurses and physicians frequently came across as lacking confidence in their own nutritional knowledge. Several professionals said they found nutrition confusing, and there was often uncertainty when talking about nutritional concepts. Rather than advising patients themselves, many professionals said they would refer patients to a dietitian, especially if they felt the patient required specific nutritional advice. They mentioned that a large amount of often contradictory information from science, the media, and the government were often the major sources of the confusion about diet-related diseases. Health professionals highlighted the contradictory information as a source of confusion for themselves, their patients and the general public. A few health professionals blamed their lack of self confidence in this matter on the

lack of nutrition education they received during their training and in continued education. Few health professionals made any reference to science when justifying or explaining their nutritional advice. In those few instances where the health professional did appeal to science, it was in a broad and vague manner.

Despite the fact that they are not able to remain current in the advances in nutrition science, participants have a communal understanding that there is a need for more clinical trials so that the current dietary guidelines may change. They agree that more clinical trials should be conducted to support new dietary trends or to break dietary stigmas, for instance, those regarding dietary fats and its effects on cholesterol levels. The health professionals interviewed in this study used a range of jargon and diet statements that were stated as fact, such as ‘*balanced diet’* ‘*sustainable* diet’. These ‘catchphrases’ can be defined as phrases or statements that are repeated without any justification or attempt to explain them, as if they were obvious. Some of these ‘catchphrases’ appeared when they had to define a healthy or unhealthy diet. Moreover, several statements appeared when they were asked about the type of advice given to patients, especially from the two dietitians. ‘*Because it is low carbohydrate, there are certain food groups that they kind of remove and we know that we should be eating all types of vegetables. There shouldn’t be one that you should cut out’*.

Another aspect considered in this theme takes into consideration statements about nutrition that provide insight into the beliefs of the health professional. Phrases such as *‘obviously’,* ‘we *all know’,* and ‘we *have known for years’* demonstrate that the health professionals hold these nutritional beliefs to be true and beyond doubt. Some nutritional statements were unique, and sometimes quite specific advice was given about a specific food. Some also provided broad nutritional statements without any context, explanation, definition or understanding of the statement being demonstrated. The health professionals said that they

have an idea of what are considered problematic foods based on their understanding and seemed to agree on the foods that they consider unhealthy or that are causing medical problems.

There was also some consistent advice that was tailored to specific conditions. For instance, low salt for hypertension and portion control and exercise for obesity. The professionals agreed indirectly that healthy food improves immunity, therefore, human immunodeficiency virus (HIV) patients should be encouraged to eat healthy foods because their immune system is compromised. In general, the cohort thought that good advice would be to promote physical activity, cut out simple sugar, stop or reduce smoking, lower salt intake, reduce alcohol, not to eat spicy food, remove the skin off chicken, remove fat from red meat, and zero sugar sodas instead of regular ones. The above advice was consistent among the professions. There were, however, some differences in their approach to nutrition, based on profession. Although there were only two dietitians, the one approach was quite simple and general, namely 'portion and moderation'. For example, when talking about sugar addiction, it was thought unrealistic to completely exclude sugar from their patients' diet but to simply reduce it and adapt the diet to the patient's life and circumstances instead.

Physicians and nurses, when advising patients, tend to be more specific by defining some foods as problematic and causative of chronic disease. Of those foods, high sugar and fat content foods were the majority identified as problematic. Examples of such foods were described as: instant or convenience food, inexpensive and easy to access, served in large portions, high-starch content, sugary drinks, '*chippies and sweeties'*, junk food, high calorie food, salty food, '*braai-ed'* [*cooked on a barbecue*] meat and more. When advising on healthy food alternatives, health professionals referred to the following: vegetables and fruits, small portions, oats, three meals a day, snacks in between (apples, whole-wheat bread with peanut butter and crackers biscuits). Some also mentioned low fat foods, eating chicken instead of red meat and less carbohydrates. Some mentioned that they advised their patients to eat food in

their natural state, not too over processed, in moderation and avoid sauces. In general, the advice on practicing physical activity seems to be non-specific and used when explaining healthy living behaviours and to promote health.

Theme 2. LCHF: Controversies and beliefs

Almost all the health professionals had heard of the LCHF diet. For those who did not know about the diet and for clarity for the others, it was defined as: *'A low-carbohydrate high-fat (LCHF) diet is a way of eating that restricts the amount of carbohydrates and increases the number of healthy fats that you eat to optimize your health and nutrition'.* The statements on this theme refer to the health professionals talking about their own knowledge of the diet. Some knew LCHF diets as the *Banting* or Tim Noakes diet, as they said that these terms have been popularised over the past few years in South Africa. Most professionals learnt of the LCHF diet through social media. Mostly, they defined LCHF as a diet that promotes unprocessed foods. Some of the cohort had a clear understanding of the diet and the foods that are recommended but most did not. Regarding their opinions on LCHF diets, many of them mentioned some catch phrases such as: '*long term health' (Female physician) and 'not sustainable' (Female dietitian)* as a general understanding of this diet. A relatively large number of participants had some personal experience or first-hand knowledge of LCHF diets. These accounts were predominantly positive, but some explained why they stopped following a LCHF diet, given the potential challenges one may face on a LCHF diet. These include a lack of support from relatives and the perceived expense of it.

Those who had personally followed the diet mentioned that they experienced that they needed to eat less, had increased energy levels and fewer cravings. These same individuals stated that they were initially sceptical of the LCHF diet, as it contrasted with their university nutrition education. Although some mentioned that according to them the diet limits one from eating what they considered essential foods - such as fruit or fibre -, that one should eat a

complete diet, and that the body requires starch. The heightened public controversy surrounding the LCHF diet initially increased the health professionals' hesitancy. They expressed concerns about the diet and reported sharing these concerns with patients who were considering or already following an LCHF diet. Overall, the professionals were primarily worried about the high fat content of LCHF diets. Some also noted that sustaining the diet could be challenging due to the high cost of LCHF foods, leading to the common perception that the diet is expensive and therefore difficult for many patients to maintain. On average, they were prepared to be supportive of the diets but would monitor patients to see if there are any harmful effects. In some of the health professionals there was a high degree of scepticism around the efficacy of LCHF to treat metabolic disorders, largely due to the controversy surrounding the LCHF diet and a lack of scientific consensus. Specifically, some common factors that made them sceptical were the sustainability of the diet, the affordability, the amount of meat, the exclusion of an entire macronutrient ('no carbohydrate at all') and, mostly, the fat intake due to the apparent link to cholesterol levels. Although, some mentioned that they were aware that their perceptions regarding cholesterol levels and dietary fats might be outdated since they did not have access to enough nutrition information.

Moreover, some were concerned that patients do not really understand the diet, that they lack information and that it would be difficult for patients to change their dietary behaviours to this diet and to monitor patients on this diet. When asked about introducing a LCHF diet to treat or prevent metabolic conditions, health professionals said that they would support it but not advocate it due to the above mentioned concerns. This led them to a cautious and vigilant attitude regarding their patients who followed a LCHF diet. Alternative reasons for this attitude include beliefs that the LCHF diet is a restrictive diet and that no vegetables should be cut out of a nutritional plan. The professionals said that they believed that their patients do not have enough knowledge about their own health prior to embarking on a LCHF

diet – further reducing their willingness to advocate. The two dietitians mentioned that adherence is a problem and that the most effective nutritional approach could be LCHF or a individualised one. Hence, they do not advocate LCHF diets as they do not consider it applicable to everyone.

Theme 3. EBSA's role in community nutrition

Some of the interviewees were familiar with the EBSA program, but the majority were introduced to the program for the first time during the interview. Those who knew about EBSA had patients who had participated in the program. The EBSA program was defined to all participants as: *'Eat Better South Africa is a nutrition education program for people from under-resourced communities that promotes low-carbohydrate high-fat diet or Banting diet to prevent or treat metabolic conditions. It consists of groups of people that meet every week for an educational session for a period of six weeks'.*

This theme involves the thoughts and opinions provided by the health professionals specific to the EBSA program. Interviewees mostly highlighted the importance of group support to successfully achieve a lifestyle behaviour change. Some believed it to be a good initiative to change the way people think, some concerns regarding the program are also related to concerns about LCHF diets. The concept of EBSA stimulated stress on the importance of support for the healthcare system both inside and outside of hospital facilities. They also stated that health professionals would appreciate having a similar nutrition education program to give them the tools to better address diet-related diseases. The majority of participants said they would support the patient but were specific about how they would offer this support:

Despite this attitude towards professional patient support, group support was considered the most valuable aspect of the EBSA program. In their opinion, behaviour change is not easy to achieve as an individual, and it is easier in a group. Some professionals valued the education aspect of the EBSA program. They indicated that, if more unhealthy people participated in support groups such as EBSA, there would be less pressure on hospital facilities

from chronic diseases. EBSA was seen as a good initiative, and that it could change the way people think. The only concerns regarding the EBSA program were those related to the LCHF diet that were mentioned before. There were suggestions for community implementation and adherence to a LCHF diet – that it needs to be tailored to food prices, accessibility and sustainability. They stated that it is necessary to ask whether people can afford it and what the food options are in their communities and surroundings because it has to be sustainable. Some health professionals even provided suggestions that EBSA could incorporate, regarding the food prices and how to approach the sustainability of the programs in communities where poverty is a common challenge.

Theme 4. Understanding socioeconomic determinants of health

This theme emerged from questions pertaining to reasons why people from poor communities (patients) eat in the way they eat. The professionals spoke in general about the challenges that people living in under-resourced communities face. Thus, they are quite aware of the community challenges to healthy living. They specifically mentioned those related to poverty and the social determinants of health found in communities such as Ocean View or Masiphumelele. Some of the health professionals interviewed live in the same communities as their patients. They have great insight into the rationale behind food choices and the challenges that patients face. These communities are commonly known, usually appearing in the media because of crime, violence and safety concerns. Moreover, this theme is also a combination of those codes related to accessibility, affordability, understanding and safety regarding food and food resources. Some health professionals acknowledged that *'Food is not a priority. (Male physician)'* explaining that people from under-resourced communities are in a survival mode. The price of food was often cited as an issue, emphasizing that healthy foods are more expensive than unhealthy food. Specifically, that meat is extremely expensive, but others pointed out that vegetables are also expensive.

The interviewees cited poverty or lack of resources due to socio-economic circumstances as a challenge to healthy lifestyle choices and changes. Communities such as Ocean View and Masiphumelele are considered under-resourced communities with a high rate of unemployment, gangsterism, lack of resources, lack of housing and limited access to health and proper nutrition education [48,49]. Unemployment appeared to be a major contributing factor to the high level of poverty in these communities. Health Professionals also considered a lack of education as an obstacle to adopting healthy lifestyle choices, change, or nutritional interventions. They recognised that nutrition education could have a huge beneficial influence on unhealthy habits. Alcohol abuse was mentioned a few times during the interviews and the health professionals said that it can be a problem for some people, as it leads to increased rates of crime in addition to perpetuating unhealthy habits and poor adherence. Compliance to the medication/treatment/lifestyle advice was identified as a challenge for the patients from the health professionals' perspective.

Health professionals acknowledged that culture and tradition around food and nutrition has a significant role in a patient's lifestyle choices and that this is heavily influenced by their upbringing. Cultural stigmatization occurs around weight and physical appearance, especially for women. Slim people are regarded as being poor, malnourished and unhealthy, yet large to obese people are seen as wealthy and healthy [50]. The health professionals stated that there are gender differences in the communities living conditions. The women's roles in the community and household were also highlighted - women are usually responsible for cooking yet have to deal with gender role challenges [51]. Some women would have to manage household duties while navigating traditional expectations that limit their decision-making power, particularly regarding health and economic autonomy [51]. Another aspect that appeared to be important according to health professionals was that these communities are made up of different people from diverse races and cultures. Relocation from rural areas to

cities for greater job opportunities was considered a potential problem regarding food habits. When people relocate, especially from rural to more urban environments, there is less opportunity to move and less physical work, yet they want to eat the same diet. This diet is usually high in carbohydrates, but in rural areas people tend to have larger and healthier sources of food through gardening or farming. When they come to the cities, they tend to eat more processed foods and poorly [52].

When talking about traditional ways of eating or upbringing, the health professionals found that, despite dispensing nutritional advice, patients do not follow the recommendations. They spoke about a lack of compliance from patients who were largely averse to different foods. The health professionals recognise that unhealthy ultra-processed foods are being eaten because they are enjoyable. This does not aid in compliance to dietary advice or education and ties in with food addiction and eating disorders as medical conditions. An interesting aspect of the culture in these communities mentioned by health professionals is one of food and status. According to them, people who lack resources desire to appear as if they can afford foods that they cannot, and there is a certain status attached to those ultra-processed foods from specific well-known brands.

Discussion

This study is the first to explore health professionals' perceptions of a community-based program promoting LCHF diets in under-resourced South African communities. The findings reveal a general alignment with conventional dietary guidelines among health professionals, who emphasize the importance of vegetables and fruits while expressing concerns about dietary fats and meat [53]. Physicians and nurses acknowledged their limited expertise in nutrition but recognized the critical role of nutritional education in disease prevention. However, they also highlighted the challenges of staying updated with evolving nutritional science and expressed uncertainty in providing dietary guidance due to the constant influx of new information [54].

This reflects a broader issue of confusion and inconsistency in nutritional advice, which can undermine the effectiveness of health promotion efforts.

The study also found that different healthcare professions approach nutrition advice differently. Physicians admitted to having some nutritional knowledge but lacked confidence in offering dietary guidance, while nurses, who are often the primary healthcare providers, demonstrated more openness to new information and a greater willingness to provide lifestyle counselling, particularly in managing non-communicable diseases (NCDs) [55-59]. An in-depth qualitative investigation with nurses delved into their encounters while counselling diabetes patients on diet, exercise, and smoking cessation [59]. Nurses highlighted challenges like patients' limited knowledge and motivation, as well as their own counselling skills and time constraints, hindering effective lifestyle counselling. Despite these obstacles, the study revealed nurses' readiness to offer weight management advice as part of their professional duties, aligning with previous research findings [58, 59]. Dietitians, though fewer in number, adhered more strictly to existing dietary guidelines and were cautious about endorsing LCHF diets due to concerns about their sustainability and potential health risks [60].

Despite the cautious approach of most health professionals, there was an acknowledgment of the potential benefits of LCHF diets in clinical nutrition, especially if patients are closely monitored. However, concerns about controversies surrounding meat and dietary fat intake, the cost and variety of recommended foods, and the alignment with national dietary guidelines were prevalent [34, 61, 62]. The concerns raised by most of the health professionals were linked to the national guideline recommendations that have been recently questioned in recent scientific reviews [62]. While most health professionals claimed they would support patients, their approach often inadvertently discouraged them, resembling backhanded support, explicitly noted in some instances. Alternatively, physicians and nurses

sometimes directed patients to dietitians, who might dissuade them from LCHF diets in favour of a more balanced macronutrient approach, as revealed in interviews.

Some study participants had personal experiences with or first-hand knowledge of LCHF diets from friends or family, which tended to be positive. Interestingly, despite their cautiousness in endorsing or advocating for the diet, some had personally experimented with it and experienced favourable results, reflecting findings from other literature where individuals reported improved well-being, weight loss, and reduced hunger on the diet [63-67]. When discussing the LCHF diet and introducing the EBSA program as a nutrition and health education initiative promoting this approach, health professionals showed interest in the concept of group support, suggesting potential backing for community-based programs that provide such support. The availability of group support within the program was seen as a catalyst for encouraging dietary behavior changes. However, this interest did not necessarily translate into full endorsement of the LCHF diet in communities, as concerns persisted about various controversies, gaps in education, and socioeconomic challenges like poverty. While some professionals acknowledged that the program might be effective in changing perceptions about diet, concerns about the LCHF approach were also raised.

There was a general recognition of the challenging environmental conditions in under-resourced South African communities, with food insecurity emerging as a significant concern due to issues of accessibility and affordability. Additionally, although not explicitly highlighted, discussions also touched on women's safety concerns. Cultural influences and traditional dietary habits were frequently mentioned, echoing previous research [68-70]. These factors were seen as obstacles to both individual and community well-being. Health professionals often framed these challenges as justifications for patients' difficulties in adhering to medical advice, reflecting a tendency to downplay patients' aspirations for better health. This rationalization of limited intervention in nutritional guidance aligns with previous observations

[54]. This perspective connects with the notion of "backhanded support" when it comes to assisting patients interested in following an LCHF diet. Health professionals acknowledged that unhealthy foods are often consumed because they are enjoyable [55,60,71-73], linking this to concepts of food addiction and predictors of problematic eating behaviours. Notably, healthcare providers, particularly dietitians, appeared hesitant to challenge these preferences among participants.

Based on the study findings, EBSA could improve their program by offering targeted training for healthcare providers, particularly nurses and physicians, to boost their confidence in recommending LCHF diets. This training should address misconceptions about dietary fats and provide strategies for monitoring patients effectively. Additionally, EBSA could develop resources that consider the socioeconomic challenges and cultural influences in under-resourced communities, offering practical and affordable dietary advice. Enhancing the group support component could also strengthen community involvement and help sustain dietary changes, leading to better health outcomes. This study is a pioneering effort to explore health professionals' views on a community-based program promoting LCHF diets in underprivileged South African communities. Through qualitative interviews, it reveals that health professionals largely align with conventional dietary guidelines, emphasizing vegetables and fruits while expressing concerns about fats and meat. The diverse perspectives of physicians, nurses, and dietitians offer a comprehensive view, but the uneven sample size, limited facility access, and the affiliation of one dietitian with an NGO may introduce biases. While highlighting the crucial role of nurses in health promotion, the study suggests that further research is needed to address its limitations and deepen our understanding of the complex factors influencing healthcare practices. Despite these challenges, the study offers valuable insights into health professionals' attitudes towards nutritional advice and LCHF diets, contributing to discussions on community healthcare and NCD management.

Conclusions

The scientific controversy surrounding LCHF diets reflects broader challenges in dietary interventions and the limitations of nutritional epidemiology. Health professionals generally exhibited a supportive but non-advocating stance towards LCHF diets, reflecting the ongoing debate and uncertainty in clinical practice regarding therapeutic nutritional interventions. Given the popularity of LCHF diets in South Africa, healthcare professionals are likely to encounter patients interested in or already following such diets. While international guidelines increasingly endorse LCHF diets, confusion and knowledge gaps persist, particularly concerning patients with complex health conditions. For these patients, including those on multiple medications or with comorbidities like renal disease, LCHF diets should be supported by experienced healthcare professionals to ensure optimal nutritional intake. Although long-term compliance and sustainability of carbohydrate restriction remain uncertain, existing evidence suggests that LCHF diets can be a legitimate and potentially effective option for managing diet-related chronic diseases.

Funding:

This research received funding from The Noakes Foundation and The National Research Federation of South Africa.

Acknowledgments:

We acknowledge the health professionals who volunteered to participate in this study, and Cyber Transcription for transcriptions. Thank you to the hospitals and non-profit organizations for allowing the interviews to be conducted in their facilities.

Data availability:

Data cannot be shared publicly as participants did not give consent for their transcripts to be shared in this manner. Since the consent statement approved by the Human Research Ethics Committee of the University of Cape Town, and signed by the participants, did not

include the provision that data would be made publicly available, we do not have participant consent to share this data. Requests for anonymized data can be made to the Principal Investigator of the study, Georgina Pujol-Busquets (georgipbg@uoc.edu).

Conflicts of Interest:

The authors declare no conflicts of interest. G.P.B. is a recipient of the Tim and Marilyn Noakes postdoctoral fellowship. The funders had no role in the design of the study; in the collection, analysis, or interpretation of data; in the writing of the manuscript; or in the decision to publish the results.

References

1. The Constitution and public health policy. Chapter 2. Health & Democracy. [Internet]. [cited 2024 Mar 17]. Available from: https://section27.org.za/wp-content/uploads/2010/04/Chapter2.pdf
2. Maphumulo WT, Bhengu BR. Challenges of quality improvement in the healthcare of South Africa post-apartheid: A critical review. Curationis. 2019;42(1):1–9. doi:10.4102/curationis.v42i1.1901.
3. Stuckler D, Basu S, Mckee M. Health Care Capacity and Allocations Among South Africa’s Provinces: Infrastructure-Inequality Traps After the End of Apartheid. Am J Public Health. 2011;101(1):165–72. doi:10.2105/AJPH.2009.184895.
4. Baker A. What South Africa Can Teach Us as Worldwide Inequality Grows. Time. 2019;1. [Internet]. [cited 2024 Mar 17]. Available from: https://time.com/longform/south-africa-unequal-country/
5. Oladipo JA. Utilization of health care services in rural and urban areas: a determinant factor in planning and managing health care delivery systems. Afr Health Sci. 2014;14(2):322-33. doi:10.4314/ahs.v14i2.6.

6. Turok I. Urbanisation and development in South Africa: economic imperatives, spatial distortions and strategic responses. Urbanisation and Emerging Population Issues: Working Paper; 8. London: International Institute for Environment and Development (IIED). [Internet]. [cited 2024 Mar 17]. Available from: http://hdl.handle.net/20.500.11910/3082.
7. Kon ZR, Lackan N. Ethnic Disparities in Access to Care in Post-Apartheid South Africa. Am J Public Health. 2008;98(12):2272–7. doi: 10.2105/AJPH.2007.127829.
8. Voget U. Professional nurses' lived experiences of moral distress at a district hospital. [Master's Thesis]. Stellenbosch University, South Africa; 2017. Available from: https://core.ac.uk/download/pdf/188221241.pdf.
9. Coovadia H, Jewkes R, Barron P, Sanders D, McIntyre D. The health and health system of South Africa: historical roots of current public health challenges. Lancet. 2009;374(9692):817–34. doi:10.1016/S0140-6736(09)60951-X.
10. Kruger HS, Puoane T, Senekal M, van der Merwe M-T. Obesity in South Africa: challenges for government and health professionals. Public Health Nutr. 2005;8(5):491–500. doi: 10.1079/phn2005785.
11. Mayosi BM, Lawn JE, Niekerk A van, Bradshaw D, Karim SSA, Coovadia HM, et al. Health in South Africa: changes and challenges since 2009. Lancet. 2012;380(9858):5–19. doi: 10.1016/S0140-6736(12)61814-5.
12. Sanders D, Chopra M. Key challenges to achieving health for all in an inequitable society: The case of South Africa. Am J Public Health. 2006;96(1):73–8. doi: 10.2105/AJPH.2005.062679.
13. Crowley J, Ball L, Hiddink GJ. Nutrition in medical education: a systematic review. Lancet Planet Health. 2019;3(9). doi: 10.1016/S2542-5196(19)30171-8.
14. Gallegos D. Global challenges and opportunities for dietitians. Nutr Diet. 2020;77:179-81. doi: 10.1111/1747-0080.12614.

15. Kohlmeier M, Nowson CA, DiMaria-Ghalili RA, Ray S. Nutrition Education for the Health Care Professions. J Biomed Educ. 2015;2015:1–2. doi: 10.1155/2015/380917.

16. Köse S, Yildirim G. The Effect of a Nutrition Education. Int J Caring Sci. 2020;13(6):573–82. [Internet]. [cited 2024 Mar 17]. Available from: https://www.internationaljournalofcaringsciences.org/docs/63_selmin_%20original_13_1.pdf

17. Riddell MA, Edwards N, Thompson SR, Bernabe-Ortiz A, Praveen D, Johnson C, et al. Developing consensus measures for global programs: Lessons from the Global Alliance for Chronic Diseases Hypertension research program. Global Health. 2017;13(1):1–12. doi:10.1186/s12992-017-0242-8.

18. Popkin BM. Global nutrition dynamics: the world is shifting rapidly toward a diet linked with noncommunicable diseases. Am J Clin Nutr. 2006;84(2):289-98. doi: 10.1093/ajcn/84.1.289.

19. Beckfield J, Olafsdottir S, Bakhtiari E. Health Inequalities in Global Context. Am Behav Sci. 2013;57(8):1014-1039. doi: 10.1177/0002764213487343.

20. Perez-Escamilla R, Bermudez O, Buccini GS, Kumanyika S, Lutter CK, Monsivais P, et al. Nutrition disparities and the global burden of malnutrition. BMJ. 2018;361:k2252. doi: 10.1136/bmj.k2252.

21. Kris-Etherton PM, Akabas SR, Douglas P, et al. Nutrition competencies in health professionals' education and training: a new paradigm. Adv Nutr. 2015;6(1):83–87. doi:10.3945/an.114.006734.

22. Viswanath K, Bond K. Social Determinants and Nutrition: Reflections on the Role of Communication. J Nutr Educ Behav. 2007;39(2). doi: 10.1016/j.jneb.2006.07.008.

23. Burgess A. Nutrition Education in Public Health Programs—What Have We Learned? Am J Public Health. 1961;51(11):1715–26. doi: 10.2105/ajph.51.11.1715.

24. Baute V, Sampath-Kumar R, Nelson S, Basil B. Nutrition Education for the Health-care Provider Improves Patient Outcomes. Glob Adv Health Med. 2018;7:216495611879599.
25. Schultz JA. Community-based nutrition education: documentation and evaluation of effectiveness [Master's Thesis]. Iowa State University; 2015. Available from: https://dr.lib.iastate.edu/handle/20.500.12876/28900/
26. Oldewage-Theron WH, Egal A. Impact of nutrition education on nutrition knowledge of public school educators in South Africa: A pilot study. Health SA Gesondheid. 2012;17(1). [Internet]. [cited 2024 Mar 17]. Available from: http://www.scielo.org.za/scielo.php?script=sci_arttext&pid=S2071-97362012000100011&lng=en&nrm=iso
27. Rustad C, Smith C. Nutrition knowledge and associated behaviour changes in a holistic, short-term nutrition education intervention with low-income women. J Nutr Educ Behav. 2013;45(6):490–8. doi: 10.1016/j.jneb.2013.06.009.
28. Walsh CM, Dannhauser A, Joubert G. Impact of a nutrition education programme on nutrition knowledge and dietary practices of lower socioeconomic communities in the Free State and Northern Cape. S Afr J Clin Nutr. 2003;16(3):89–95. [Internet]. [cited 2024 Mar 17]. Available from: https://www.semanticscholar.org/paper/Impact-of-a-nutrition-education-programme-on-and-of-Walsh-Dannhauser/33731bd4dc0e3c542fbd87cf837f380cc802eada
29. Whitehead D, Wang Y, Wang J, Zhang J, Sun Z, Xie C. Health promotion and health education practice: Nurses' perceptions. J Adv Nurs. 2008;61(2):181–7. doi: 10.1111/j.1365-2648.2007.04479.x.
30. Martin L, Leveritt MD, Desbrow B, Ball LE. The self-perceived knowledge, skills and attitudes of Australian practice nurses in providing nutrition care to patients with chronic disease. Fam Pract. 2014;31(2):201-8. doi: 10.1093/fampra/cmt070.

31. Volek JS, Feinman RD. Carbohydrate restriction improves the features of Metabolic Syndrome. Nutr Metab. 2005;2:1–17. doi: 10.1186/1743-7075-2-31.
32. Feinman RD, Volek JS. Low carbohydrate diets improve atherogenic dyslipidemia even in the absence of weight loss. Nutr Metab. 2006;3:24. doi: 10.1186/1743-7075-3-24.
33. Feinman RD, Vernon MC, Westman EC. Low carbohydrate diets in family practice: What can we learn from an internet-based support group. Nutr J. 2006;5(1). doi: 10.1186/1475-2891-5-26.
34. Sboros S. The Noakes Trial, two years later. 2020. [Internet]. [cited 2024 Mar 17]. Available from: https://www.nmsociety.org/the-scene/noakestrial.
35. Murdoch C, Unwin D, Cavan D, Cucuzzella M, Patel M. Adapting diabetes medication for low carbohydrate management of type 2 diabetes: a practical guide. Br J Gen Pract. 2019;69(684):360-1. doi: 10.3399/bjgp19X704525.
36. Mozaffarian D. Dietary and Policy Priorities for Cardiovascular Disease, Diabetes, and Obesity. Circulation. 2016;133(2):187–225. doi: 10.1161/CIRCULATIONAHA.115.018585.
37. Banting WB. Letter on Corpulence, Addressed to the Public Third Edition. Classic in Obesity. 1987. [Internet]. [cited 2024 Mar 17]. Available from: https://onlinelibrary.wiley.com/doi/pdf/10.1002/j.1550-8528.1993.tb00605.x.
38. Pujol-Busquets G, Smith J, Larmuth K, et al. Exploring the Perceptions of Women from Under-Resourced South African Communities about Participating in a Low-Carbohydrate High-Fat Nutrition and Health Education Program: A Qualitative Focus Group Study. Nutrients. 2020;12(4):894. doi: 10.3390/nu12040894.
39. Pujol-Busquets G, Smith J, Fàbregues S, et al. Community Assessment for a Low-Carbohydrate Nutrition Education Program in South Africa. Nutrients. 2023;15(1):67. doi: 10.3390/nu15010067.

40. Pujol-Busquets G, Smith J, Fàbregues S, Bach-Faig A, Larmuth K. Mixed methods evaluation of a low-carbohydrate high-fat nutrition education program for women from underserved communities in South Africa. Appetite. 2025;204:107725. doi: 10.1016/j.appet.2024.107725.
41. Johnston BC, Kanters S, Bandayrel K, et al. Comparison of weight loss among named diet programs in overweight and obese adults: a meta-analysis. JAMA. 2014;312(9):923-33. doi: 10.1001/jama.2014.10397.
42. Franz MJ, VanWormer JJ, Crain AL, et al. Weight-loss outcomes: a systematic review and meta-analysis of weight-loss clinical trials with a minimum 1-year follow-up. J Am Diet Assoc. 2007;107(10):1755-67. doi: 10.1016/j.jada.2007.07.017.
43. Digby A. The global and the local: changes at Groote Schuur Hospital. Lancet. 2009;374(9692):778–9. doi: 10.1016/S0140-6736(09)61582-8.
44. Brink JG, Hassoulas J. The first human heart transplant and further advances in cardiac transplantation at Groote Schuur Hospital and the University of Cape Town. Cardiovasc J Afr. 2009;20(1):31-5. PMID: 19287813; PMCID: PMC4200566.
45. Adler P, Adler P. How many qualitative interviews is enough? Natl Cent Res Methods Rev Pap. 2012;1–42. [Internet]. [cited 2024 Mar 17]. Available from: http://eprints.ncrm.ac.uk/2273/.
46. Dworkin SL. Sample Size Policy for Qualitative Studies Using In-Depth Interviews. Arch Sex Behav. 2012;41(6):1319–20. doi: 10.1007/s10508-012-0016-6.
47. Braun V, Clarke V. Using thematic analysis in psychology. Qual Res Psychol. 2006;3(2):77–101. doi: 10.1191/1478088706qp063oa.
48. Alaba O, Chola L. The social determinants of multimorbidity in South Africa. Int J Equity Health. 2013;12(1):1–10. doi: 10.1186/1475-9276-12-63.

49. Seekings J, Nattrass N. Class, Race, and Inequality in South Africa. Foreign Affairs. 2006;85(3):173. doi: 10.2307/20032025.
50. Puoane T, Fourie JM, Shapiro M, Rosling L, Tshaka NC, Oelefse A. "Big is beautiful" - an exploration with urban black community health workers in a South African township. S Afr J Clin Nutr. 2005;18(1):6-15. doi: 10.1080/16070658.2005.11734033.
51. Ndinda C, Uzodike UO, Chimbwete C, Pool R. Gender relations in the context of HIV/AIDS in rural South Africa. AIDS Care. 2007;19(7):844-9. doi: 10.1080/09540120701203923.
52. Vorster HH, Kruger A, Margetts BM. The nutrition transition in Africa: Can it be steered into a more positive direction? Nutrients. 2011;3(4):429-41. doi: 10.3390/nu3040429.
53. Steyn N, Ochse R. Food-Based Dietary Guidelines for South Africa: “Enjoy a variety of foods”: as a food-based dietary guideline. S Afr J Nutr. 2013;26(3):13–7. (Supplement):S1-S164. [Internet]. [cited 2024 Mar 17]. Available from: https://openknowledge.fao.org/server/api/core/bitstreams/2cf12134-bb2d-47b1-92b0-403b028c60ad/content
54. Hunter-Adams J, Battersby J. Health care providers’ perspectives of diet-related non-communicable disease in South Africa. BMC Public Health. 2020;20(1). doi: 10.1186/s12889-020-8364-y.
55. McKinlay E, Plumridge L, McBain L, McLeod D, Pullon S, Brown S. “What sort of health promotion are you talking about?”: A discourse analysis of the talk of general practitioners. Soc Sci Med. 2005;60(5):1099–106. doi: 10.1016/j.socscimed.2004.06.041.
56. Smolowitz J, Speakman E, Wojnar D, Whelan EM, Ulrich S, Hayes C, et al. Role of the registered nurse in primary health care: Meeting health care needs in the 21st century. Nurs Outlook. 2015;63(2):130–6. doi: 10.1016/j.outlook.2014.08.004.

57. Barton AJ, Skiba DJ, Sorensen L. Preparing the next generation of advanced practice nurses. Stud Health Technol Inform. 2004;107(Pt 2):860-4. PMID: 15360934.
58. Xu X, Parker D, Ferguson C, Hickman L. Where is the nurse in nutritional care? Contemp Nurse. 2017;53(3):267-70. doi: 10.1080/10376178.2017.1370782.
59. Jansink R, Braspenning J, van der Weijden T, Elwyn G, Grol R. Primary care nurses struggle with lifestyle counseling in diabetes care: A qualitative analysis. BMC Fam Pract. 2010;11. doi: 10.1186/1471-2296-11-41.
60. Marshall AP, Takefala T, Williams LT, Spencer A, Grealish L, Roberts S. Health professional practices and their influence on nutritional intake of hospitalised patients. Int J Nurs Sci. 2019;6(2):162–8. doi: 10.1016/j.ijnss.2019.03.008.
61. Holmberg C. Politicization of the Low-Carb High-Fat Diet in Sweden, Promoted On Social Media by Non-Conventional Experts. Int J E-Polit. 2015;6(3):27–42. doi: 10.4018/ijep.2015070103.
62. Guidelines Advisory Committee D. Scientific Report of the Dietary Guidelines Advisory Committee Advisory Report to the Secretary of Agriculture and Secretary of Health and Human Services. 2020. [Internet]. [cited 2024 Mar 17]. Available from: https://www.dietaryguidelines.gov/2020-advisory-committee-report.
63. McPhee J. Exploring the Acceptability of, and Adherence to, a Carbohydrate-Restricted, Higher Fat Diet as an Instrument for Weight Loss in Women Aged 40–55 Years [Master’s Thesis]. Auckland University of Technology; 2018. [Internet]. [cited 2024 Mar 17]. Available from: https://researchspace.auckland.ac.nz/docs/uoa-docs/rights.htm
64. Webster CC, Murphy TE, Larmuth KM, Noakes TD, Smith JA. Diet, Diabetes Status, and Personal Experiences of Individuals with Type 2 diabetes Who Self-Selected and Followed a Low Carbohydrate High Fat diet. Diabetes Metab Syndr Obes. 2019;12:2567–82. doi:10.2147/DMSO.S227090.

65. Jallinoja P, Niva M, Helakorpi S, Kahma N. Food choices, perceptions of healthiness, and eating motives of self-identified followers of a low-carbohydrate diet. Food Nutr Res. 2014;58:1–9. doi: 10.3402/fnr.v58.23552.

66. Clarke C, Best T. Low-carbohydrate, high-fat dieters: Characteristic food choice motivations, health perceptions and behaviours. Food Qual Prefer. 2017;62:162–71. doi: 10.1016/j.appet.2019.104324.

67. Arthur TH. Perceptions and acceptability of Low Carbohydrate, High Fat (LCHF) diets among Māori whānau in Dunedin. [Master's Thesis]. University of Otago; 2018. [Internet]. [cited 2024 Mar 17]. Available from: https://ourarchive.otago.ac.nz/esploro/

68. Aubel J. The role and influence of grandmothers on child nutrition: Culturally designated advisors and caregivers. Matern Child Nutr. 2012;8(1):19–35. doi: 10.1111/j.1740-8709.2011.00333.x.

69. Cassel J. Social and Cultural Implications of Food and Food Habits. Am J Public Health. 1957;47:732–40. doi: 10.2105/ajph.47.6.732.

70. Hartanto A, Lau IYM, Yong JC. Culture moderates the link between perceived obligation and biological health risk: Evidence of culturally distinct pathways for positive health outcomes. Soc Sci Med. 2020;244. doi: 10.1016/j.socscimed.2019.112644.

71. Rankin D, Backett-Milburn K, Platt S. Practitioner perspectives on tackling health inequalities: Findings from an evaluation of healthy living centres in Scotland. Soc Sci Med. 2009;68(5):925–32. doi: 10.1016/j.socscimed.2008.12.013.

72. Strang AB, Ager A. Psychosocial interventions: Some key issues facing practitioners. Intervention. 2003;1(3):2–12. [Internet]. [cited 2024 Mar 17]. Available from: https://citeseerx.ist.psu.edu/document?repid=rep1&type=pdf&doi=bd2177acc71bac53c13b6735ae7c918f85283aa8

73. Kok MO, Vaandrager L, Bal R, Schuit J. Practitioner opinions on health promotion interventions that work: Opening the “black box” of a linear evidence-based approach. Soc Sci Med. 2012;74(5):715–23. doi: 10.1016/j.socscimed.2011.11.021.

Click here to access/download
Supporting Information
IRB_Approval.pdf

Click here to access/download

Supporting Information

CRediT author statement.docx
