# Supplementary material for: Between support and scepticism: Health professionals’ perceptions of a nutrition education program promoting low-carbohydrate, high-fat diets in under-resourced South African communities
Source: PLoS One. 2025 Jun 23;20(6):e0325179. doi: 10.1371/journal.pone.0325179 (PMC12184999; doi:10.1371/journal.pone.0325179)
Supplement: Attachment — Submitted filename: PONE-D-24-55230_R1_Mickael Essouma comments.pdf [file pone.0325179.s006.pdf]

PLOS ONE

Between support and scepticism: Health professionals’ perceptions of a nutrition education program promoting low-carbohydrate, high-fat diets in under-resourced South African communities

--Manuscript Draft--

**Manuscript Number:**	PONE-D-24-55230R1
**Article Type:**	Research Article
**Full Title:**	Between support and scepticism: Health professionals’ perceptions of a nutrition education program promoting low-carbohydrate, high-fat diets in under-resourced South African communities
**Short Title:**	Health professionals’ views on Low-Carbohydrate diets in under-resourced South African communities
**Corresponding Author:**	Ahtisham Younas, Ph.D<br>Memorial University of Newfoundland<br>Saint John's, NL CANADA
**Keywords:**	Perspectives on nutrition; Healthcare professionals; Low-carbohydrate, high-fat diet (LCHF); Under-resourced communities; South Africa; Thematic analysis; Qualitative Research
**Abstract:**	The burden of non-communicable diseases (NCDs) continues to rise, emphasizing the need for effective dietary interventions. Programs such as Eat Better South Africa (EBSA) advocate for low-carbohydrate high-fat (LCHF) dietary choices, especially in disadvantaged communities. However, the adoption of such approaches among healthcare professionals remains contentious. This qualitative study explores healthcare professionals' perspectives on nutrition and the EBSA program, drawing on 16 in-depth individual interviews with physicians, nurses, and dietitians from False Bay Hospital, Groote Schuur Hospital, and a primary health clinic in Hout Bay, Western Cape, South Africa. Thematic analysis of the interviews revealed four main findings. Healthcare professionals lacked confidence in their nutritional knowledge, and while many were familiar with the LCHF diet, opinions varied regarding its sustainability and health implications. Concerns were raised about the high fat content and affordability of LCHF foods. Professionals acknowledged the value of group support in behaviour change, as promoted by EBSA, but expressed reservations about its strong emphasis on LCHF diets. Key challenges identified for patients included poverty, cultural beliefs, limited education, and access to nutritious foods. The findings highlight a reliance on traditional dietary advice, with uncertainties about the feasibility and affordability of LCHF diets. These findings offer novel insights into the complexities of implementing community-based dietary interventions in South Africa, with implications for policy and practice.
**Order of Authors:**	Georgina Pujol-Busquets
	Kate Larmuth
	Christopher C Webster
	James Smith
	Ahtisham Younas, Ph.D
	Sergi Fàbregues
	Anna Bach-Faig
**Opposed Reviewers:**	
**Response to Reviewers:**	Manuscript Title: Between support and scepticism: Health professionals’ views on Low-Carbohydrate diets in under-resourced South African communities.<br><br>New Tile: Between support and scepticism: Health professionals’ perceptions on a nutrition education program promoting Low-Carbohydrate, High-fat diets in under-resourced South African communities.

Powered by Editorial Manager® and ProduXion Manager® from Aries Systems Corporation

Manuscript ID: PONE-D-24-55230

Dear Editor and Esteemed Reviewers,
On behalf of all co-authors, I would like to sincerely thank the reviewers for their thoughtful and constructive feedback on our manuscript. We greatly appreciate the time and expertise that went into reviewing our work, and we are confident that the suggested revisions have significantly improved the quality and clarity of our submission. We have carefully considered each comment and have responded in detail below, outlining the changes made to the manuscript and providing clarifications where necessary.
We trust that our revisions have addressed the reviewers' concerns, and we look forward to your further consideration of our manuscript for publication in PLOS ONE.
Sincerely,
Georgina Pujol-Busquets

Reviewers' comments:
Comments to the Author
1. Is the manuscript technically sound, and do the data support the conclusions?

The manuscript must describe a technically sound piece of scientific research with data that supports the conclusions. Experiments must have been conducted rigorously, with appropriate controls, replication, and sample sizes. The conclusions must be drawn appropriately based on the data presented.

Reviewer #1: Yes

Reviewer #2: No

Reviewer #3: Yes

Reviewer #4: Partly

2. Has the statistical analysis been performed appropriately and rigorously?

Reviewer #1: N/A

Reviewer #2: No

Reviewer #3: Yes

Reviewer #4: N/A

3. Have the authors made all data underlying the findings in their manuscript fully available?

The PLOS Data policy<http://track.editorialmanager.com/CL0/http:%2F%2Fwww.plosone.org%2Fstatic%2Fpolicies.action%23sharing/1/010f019547d21ab6-08e7bc50-c91e-4e43-a149-14affdf0e8f0-000000/uoBneyU2ESJ2opnWfelmiRHTzi2lhcmhawIbFSjISQo=200> requires authors to make all data underlying the findings described in their manuscript fully available without restriction, with rare exception (please refer to the Data Availability Statement in the manuscript PDF file). The data should be provided as part of the manuscript or its supporting information, or deposited to a public repository. For example, in addition to summary statistics, the data points behind means, medians and variance measures should be available. If there are restrictions on publicly sharing data—e.g. participant privacy or use of data from a third party—those must be specified.

Reviewer #1: No

Reviewer #2: No

Reviewer #3: No

Reviewer #4: No

4. Is the manuscript presented in an intelligible fashion and written in standard English?

PLOS ONE does not copyedit accepted manuscripts, so the language in submitted articles must be clear, correct, and unambiguous. Any typographical or grammatical errors should be corrected at revision, so please note any specific errors here.

Reviewer #1: Yes

Reviewer #2: Yes

Reviewer #3: Yes

Reviewer #4: Yes

5. Review Comments to the Author

Please use the space provided to explain your answers to the questions above. You may also include additional comments for the author, including concerns about dual publication, research ethics, or publication ethics. (Please upload your review as an attachment if it exceeds 20,000 characters)

Responses to Reviewers' Comments

Reviewer #1

This study explores healthcare professionals' perspectives on nutrition and the EBSA program, drawing on 16 in-depth individual interviews with physicians, nurses, and dietitians from False Bay Hospital, Groote Schuur Hospital, and a primary health clinic in Hout Bay, Western Cape, South Africa. Thematic analysis revealed four main theme- Foundations of dietary understanding, LCHF: Controversies and beliefs; EBSA's role in community nutrition; and understanding socioeconomic determinants of health. It is nicely written, and I have no major concerns. I offer the following suggestions for your consideration:

1. While I absolutely agree with the first paragraph, I felt that it didn't align well with the study performed. Can this be more focussed on the research question? Or, perhaps, just delete it and start with the sentence in line 70 ("Despite nutrition's…")
Response:
We agree that the first paragraph did not fully align well with the study focus. As suggested, the paragraph has been modified, and the Introduction now begins with: "South Africa's public health system faces major challenges, including long wait times, resource shortages, poor management, inadequate infection control, and workforce deficits—exacerbated by inequitable resource distribution, rapid urbanization, and the dual burden of infectious and non-communicable diseases (NCDs) [1–9]."

2. What would be useful to a reader not intimately familiar with SA nutrition/health systems would be a brief (just brief, no need for >4 sentences) comparison of EBSA vs. standard dietary guidance in South Africa. Could this be added to provide this context?
Response:
We have added a brief comparison between EBSA and standard dietary guidance in South Africa in the Introduction. This contextual framing is now included to orient readers unfamiliar with South African dietary policy.

"EBSA promotes a low-carbohydrate high-fat (LCHF) dietary approach, with an emphasis on nutrient-dense, minimally processed foods [31]. The LCHF approach contrasts with the South African Food-Based Dietary Guidelines, which emphasise balanced diets with staple whole grains, moderate fat intake, and a heavy focus on plant-based foods [32]."

Reference:
32. Vorster HH, Badham JB, Venter CS. An introduction to the revised South African food-based dietary guidelines. S Afr J Clin Nutr. 2013;26(Suppl 1):S5–S12.

3. Was only selecting English speaking participants a major bias?
Response:
We acknowledge this as a limitation and have now explicitly mentioned this in the limitations section at the of the Discussion, noting that the exclusion of non-English speakers may have limited the diversity of perspectives captured.
“The diverse perspectives of physicians, nurses, and dietitians offer a comprehensive view, but the uneven sample size, limited facility access, the affiliation of one dietitian with an NGO, and the exclusion of non-English speakers may have limited the diversity of perspectives captured and introduced biases. Future studies should include non-English-speaking health professionals to capture broader cultural perspectives.”

4. Why is “original language” stated in line 153? Were all interviews not conducted in English, as stated in line 137-38?
Response:
This has been corrected to explicitly state that all interviews were conducted in English. The term “original language” has been removed to avoid confusion.

5. Was member checking performed? If not, might be good to justify why it was not done.
Response:
Member checking was not performed in this study due to both logistical and methodological considerations. Practically, the time and resource constraints inherent in the research timeline made it infeasible to re-engage participants for feedback. However, beyond these constraints, there are also epistemological and methodological concerns associated with the use of member checking in qualitative research of this nature. One common critique, as discussed by Bloor (1997), is that member validation exercises are not neutral or definitive tests of validity, but rather complex social encounters influenced by context, participant subjectivity, and the changing nature of perception. As Bloor demonstrates, members’ reactions to findings are often shaped by the passage of time, evolving perspectives, and the dynamics of the feedback process itself. Participants might reject or revise earlier statements not because the initial data were invalid, but because their current viewpoints differ, highlighting the inherently dynamic and situated nature of perceptions. Furthermore, member checking has been critiqued for its roots in positivist assumptions about data validation—that there is a singular, fixed “truth” that participants can confirm. In interpretive qualitative paradigms, such as the one guiding this study, the researcher acknowledges that data are co-constructed and context-dependent, and that seeking verification from participants can sometimes undermine the complexity and multiplicity of meanings present in qualitative data (Bloor, 1997).
Therefore, the decision not to employ member checking aligns with both practical limitations and a reflexive approach that prioritizes depth of interpretation over post hoc validation by participants.

“We did not use member checking for practical and methodological considerations. Logistically, time and resource constraints prevented following up with participants. Methodologically, we adopted a reflexive approach that recognises the dynamic and contextual nature of participants’ perceptions, which may shift over time and are not necessarily fixed or singular. Participant feedback is shaped by the circumstances in which it is elicited and does not constitute a definitive test of validity [47].”
Reference:
47. Bloor M. Techniques of validation in qualitative research: a critical commentary. In: Miller G, Dingwall R, editors. Context and method in qualitative research. London: SAGE Publications; 1997. p. 37–50.

6. Which profession usually counsels on nutrition in this setting? If it’s dietitians, would having only 2/16 participants as dietitians be a limitation of perspective?
Response:
We have clarified in the limitations section in the Discussion that while dietitians are typically the lead providers of dietary counselling, nurses and physicians in under-resourced primary care settings often deliver nutrition advice due to staff shortages.

This sampling choice, therefore, reflects real-world task-shifting common in South African healthcare.
“Additionally, while dietitians are typically the primary providers of dietary counselling, in under-resourced primary care settings, nurses and physicians often take on this role due to staff shortages [52]. This reflects the real-world task-shifting that is common in South African healthcare, where non-specialist providers must frequently step in to deliver nutrition advice. While dietitians play a central role in dietary counselling, this study included physicians and nurses to capture how nutrition messages are communicated in primary care, aligning with community health models for under-resourced settings. Future research focusing solely on dietitians could provide more specialized insights into LCHF counselling and implementation.”

7. Discussion line 448- could you provide an example of what is meant by “backhanded support”?
Response:
This phrase has been clarified in the Discussion to mean that professionals reluctantly supported patients who chose LCHF diets, not because they endorsed the diet themselves, but because they wanted to encourage patient autonomy. This illustrates a phenomenon known as therapeutic pragmatism, where professionals adopt a supportive stance despite personal doubts.
“Their reluctance stemmed from concerns about the diet’s alignment with conventional dietary guidelines, a lack of confidence in providing LCHF-specific advice, and uncertainty about its long-term health impacts. Rather than actively endorsing LCHF diets, professionals supported patients out of a desire to respect patient autonomy, a stance known as therapeutic pragmatism, where healthcare providers adopt a supportive role despite personal doubts [59]. This meant that while they outwardly agreed to assist patients following an LCHF approach, their hesitance and underlying scepticism often resulted in a lack of meaningful guidance, leaving patients to navigate dietary changes largely on their own.”
Reference:
59. Pilnick A, Hindmarsh J, Gill VT, editors. Communication in healthcare settings: policy, participation and new technologies. Chichester: Wiley-Blackwell; 2010. 160 p. ISBN: 978-1-4051-9827-1.

8. Discussion line 448-450- this is an interesting and possibly loaded interpretation. Could you expand? Is it that the doctors believed in LCHF (despite admitting limited nutrition knowledge), and were reluctant (“sometimes”) to send to dietitians because they feared that dietitians would not provide LCHF advice?
Response:
This section has been expanded at the end of the Discussion to explain that some doctors privately believed in LCHF’s potential benefits (often informed by media rather than training), but were reluctant to refer to dietitians because they expected dietitians to discourage LCHF in favor of conventional low-fat advice.
“Despite these challenges, the study offers valuable insights into health professionals' attitudes towards nutritional advice and LCHF diets, contributing to discussions on community healthcare and NCD management. Notably, some doctors privately believed in LCHF’s potential benefits—often informed more by media than formal training—but were reluctant to refer patients to dietitians, anticipating that dietitians would discourage LCHF in favor of conventional low-fat advice [13].”

9. Discussion lines 459-464; could you clarify- was the failure to endorse LCHF in communities one of the group, in that the support group itself (was this composed of patients) was not endorsing LCHF? It’s not clear
Response:
This has been revised to clarify in the Discussion that some professionals felt community-based support groups themselves were hesitant to fully endorse LCHF diets. This stemmed from uncertainty, conflicting advice from different health workers, and broader scepticism about non-traditional diets within these groups.
“Many healthcare professionals remained cautious about formally supporting the diet, reflecting a broader global trend where media-driven interest does not always translate into clinical adoption [60]. This suggests that scepticism about LCHF is not solely a matter of questioning its health implications but rather an uncertainty about how it can be practically adopted in resource-limited settings. Some professionals also noted that hesitation toward LCHF did not come solely from healthcare providers but was also

present within the support groups themselves, which were composed of community members, including patients. This reluctance within the groups stemmed from conflicting advice received from different health workers, scepticism about non-traditional diets, and broader uncertainty about the feasibility of LCHF within low-income settings [61-63]. While some professionals acknowledged that the program might be effective in changing perceptions about diet, concerns about the LCHF approach were also raised."

Reviewer #2

1. May please add some of the observations in the tabulated form.
Response:
Thank you for your suggestion. Table 2 (now Table 1) was developed initially to provide deeper insights into the themes, offering a structured view of health professionals' perspectives with definitions and direct quotes to support the qualitative analysis. By summarizing key findings while maintaining participant perspectives, we think that Table 1 effectively presents observations in a clear, accessible format. We believe it meets the request but welcome further clarification if needed.

2. It would be better if you could convert the qualitative discussion output as quantitative values, which can be statistically analyzed.
Response:
Thank you for the comment. While some qualitative studies may include elements of quantification, we do not consider it appropriate in the context of this particular study. The approach employed is interpretive, where the aim is to explore meaning and complexity rather than to measure frequency.

3. Sentences may be edited for clarity.
Response:
The manuscript has undergone thorough language editing to improve clarity and readability.

4. The presented conclusion could be part of the discussion, and a new conclusion based on the study conducted may be developed.
Response:
Thank you for your comment. The Conclusion has been rewritten to focus explicitly on the study's contributions, see the revised section:
"This study provides valuable insights into healthcare professionals' perspectives on a nutrition education program recommending LCHF diets, highlighting key challenges in their understanding and implementation within under-resourced South African communities. Professionals expressed uncertainty around LCHF, often shaped by conflicting guidelines, limited exposure to recent evidence, and scepticism about its long-term effects. The findings also reveal significant training gaps in nutrition education, with many professionals, mostly doctors and nurses, lacking confidence in providing dietary guidance beyond conventional recommendations. Additionally, socioeconomic barriers such as food affordability, cultural norms, and structural inequalities further complicate dietary behaviour change in these communities. Despite these challenges, according to participants, EBSA presents a promising model for integrating nutrition education into healthcare strategies, particularly by offering structured support and practical guidance tailored to local contexts. Future efforts should focus on bridging knowledge gaps, strengthening professional training, and ensuring that dietary interventions are both evidence-based and accessible to those who need them most."

5. Was there any control group?
Response:
As an exploratory qualitative study, no control group was used, as this approach aligns with the principles of qualitative research, which prioritise an in-depth understanding of the phenomena under investigation over comparative analysis (Yin 2011).
Reference:
Yin, R. K. (2011). Qualitative research from start to finish. Guilford Press.

Reviewer #3

Powered by Editorial Manager® and ProduXion Manager® from Aries Systems Corporation

	Title: Between support and scepticism :Health professional's views on low carbohydrate diest in under-resourced South African communities.<br>The paper is written well except the following gaps;<br>1.Content or focus: much of professional/expert skepticism is raised against the applicability or practicability of EBSA's LCHF program ,not on its health implications. All the issues under theme 4 are arguments questioning practicability.Thus, it would have been better the research was framed along that direction.<br>Response:<br>We appreciate the reviewer's insightful feedback and agree that much of the skepticism raised by professionals focused on the practical applicability of EBSA's LCHF program rather than purely on concerns about health implications. However, we believe that both aspects—uncertainty about the diet's feasibility in under-resourced settings and concerns about its long-term health effects—were present in the data. To align with the reviewer's suggestion, we have revised the Discussion section to ensure that implementation challenges—such as affordability, accessibility, and food security—are clearly emphasized as primary concerns among professionals. We have clarified that practical barriers, rather than outright rejection of LCHF as a nutritional intervention, were central to skepticism. However, we have also retained references to health-related concerns, as some professionals questioned the sustainability and long-term impact of LCHF on metabolic health, particularly in patients with comorbidities. We appreciate this valuable feedback and believe these refinements strengthen the clarity of our findings. We hope this revision adequately addresses the concern, but we welcome any further suggestions for improvement. Please see the track changes version of the manuscript to see all the changes in the Discussion.<br><br>2.Your findings go beyound the scope of your title. Your title is restricted only to Low Carbohydrate Diets, but your findings and discussions are focusing more on the controvercies over High Fat Diets. Look at the folowing on the sources of skepticism; line 35, 303, 311-312, and 420. These all are stressing on expert caution on High Fat diets than Low Carbohydrates.<br>Response:<br>We appreciate this observation and have revised the title to better reflect the scope of the study, incorporating both low carbohydrate and high fat aspects to align with the term "LCHF" (Low-Carbohydrate, High-Fat). This dual emphasis is critical as it captures the full nature of the dietary recommendations under review, consistent with international definitions of LCHF diets. Please see the Title Page document for the revised version.<br>Revised Title: "Between support and scepticism: Health professionals' perceptions on a nutrition education program promoting Low-Carbohydrate, High-fat diets in under-resourced South African communities."<br><br>3.Unsupported and contradictory statements. For example,<br>A)health professional opinions on the role of nutritional education, (line 2002-2003 contradicts with (line 421_422).<br>Response:<br>Thank you for pointing out this inconsistency regarding health professionals' views on nutritional education. We recognize that the statements in line 202-203 and line 421-422 could be interpreted as contradictory. To clarify, professionals acknowledged the importance of nutritional education in disease prevention but simultaneously expressed challenges in integrating it into their routine practice due to time constraints and limited expertise. Their recognition of its importance does not necessarily translate into their ability or confidence to provide such education effectively.<br>To resolve this contradiction, we have revised the Discussion.<br>"While physicians and nurses recognized the value of nutritional education in disease prevention, their ability to provide effective dietary counselling was hindered by systemic barriers, including time constraints, competing clinical responsibilities, and inconsistent nutritional messaging. [50]."<br><br>B)information access of professionals,(line 229 contradicts with line 430).<br>Response:<br>We appreciate the reviewer's observation regarding the apparent contradiction in how professionals discuss access to nutritional information. We acknowledge that while professionals reported struggles in keeping up with evolving nutrition science, they simultaneously relied on established dietary concepts that were not always critically

	examined. To address this, we have revised the Discussion section to clarify that health professionals acknowledged the need for more clinical trials to strengthen evidence-based dietary guidelines but continued to use established dietary terminology without necessarily scrutinizing its scientific basis. This revision ensures that both aspects, their difficulty in staying updated with nutrition science and their reliance on conventional dietary messaging, are accurately represented. To resolve this contradiction, we have revised the Discussion.<br>"While health professionals expressed difficulty in staying updated with advancements in nutrition science, they also relied heavily on widely accepted dietary principles without necessarily questioning or critically analyzing them. Furthermore, many professionals agreed that more clinical trials were needed to reassess current dietary guidelines, reflecting an awareness of the evolving nature of nutrition science. This duality, acknowledging the need for stronger evidence while continuing to use conventional dietary rhetoric, highlights the complexity of how professionals navigate nutritional knowledge in practice."<br><br>C)popularity of LCHF diets in south africa (504) contradicts with lack of support and stopping to follow those diets mentioned in the manuscript<br>Response:<br>We appreciate the reviewer's observation regarding the apparent contradiction between the reported popularity of LCHF diets in South Africa and the lack of professional support for and continued adherence to these diets. To clarify, we have revised the paragraph to distinguish between public/media-driven popularity and actual adoption by healthcare professionals and their patients. This revision ensures that the distinction between public enthusiasm and professional skepticism is made explicit, addressing the concern raised by the reviewer.<br>"While LCHF diets are widely recognized in South Africa, their popularity is largely driven by media visibility and public discourse rather than strong institutional or professional endorsement. Many healthcare professionals remained cautious about formally supporting the diet, reflecting a broader global trend where media-driven interest does not always translate into clinical adoption [60]. This suggests that scepticism about LCHF is not solely a matter of questioning its health implications but rather an uncertainty about how it can be practically adopted in resource-limited settings. Some professionals also noted that hesitation toward LCHF did not come solely from healthcare providers but was also present within the support groups themselves, which were composed of community members, including patients. This reluctance within the groups stemmed from conflicting advice received from different health workers, scepticism about non-traditional diets, and broader uncertainty about the feasibility of LCHF within low-income settings [61-63]."<br><br>D.effectiveness of LCHF, consensus guidelines on LCHF (line 74)Vs lack of scientific consensu on it (309)<br>Response:<br>We appreciate the reviewer's observation regarding the apparent contradiction between the existence of international consensus guidelines on LCHF (line 74) and the mention of a lack of scientific consensus on it (line 309). To clarify, we have revised the paragraph to distinguish between the growing international support for carbohydrate restriction in diabetes management and the remaining concerns among healthcare professionals about its long-term effects, feasibility, and sustainability. This revision ensures that both the established guidelines and the persisting concerns among practitioners are accurately represented.<br>"Their primarily concern centred around the high fat content of LCHF diets and whether the diet was financially sustainable for patients from low-income communities. Many professionals believed that LCHF foods were expensive, making it difficult for patients to maintain the diet long-term. Some were willing to support patients who chose to follow LCHF, but their support was often condition on closely monitoring patients for potential negative effects. While international consensus guidelines recognize carbohydrate restriction as an effective approach for managing type 2 diabetes, scepticism remains among some health professionals, particularly regarding its long-term sustainability and applicability in under-resourced settings. Some health professionals questioned whether LCHF could effectively treat metabolic disorders, largely due to ongoing controversy surrounding the diet and the lack of full scientific agreement on its long-term effects."

Powered by Editorial Manager® and ProduXion Manager® from Aries Systems Corporation

	E. Venue of meeting interviewee: private venue(line 137) Vs in the health facilities(519)<br>F.Duration of contact with interviewees: one hour(137) Vs thirty minutes(141)<br>Response:<br>This has been corrected for consistency. Most interviews were conducted at private venues within the participating health facilities, ensuring both privacy and accessibility and they were scheduled for 30-60 minutes allowing for flexibility depending on participant availability and depth of discussion. Please see the Participants section for the revision.<br>"The participants met individually with a researcher for approximately thirty minutes to an hour in a private venue within the participating health facilities, ensuring both privacy and accessibility, where they signed the informed consent form and were interviewed in English."<br><br>G.selection of interviwee was done from facilities that run EBSA program nutrition education (113). That means they are expected to be knowledgabe about the program.However, you have written (line 329-330) that "majority were introduced to (EBSA) program for the first time during the interview"<br>Response:<br>We clarified that although participants were drawn from facilities serving communities where EBSA had run programs, this did not guarantee their direct involvement in or awareness of the program itself. This is now explained explicitly in the Participant section.<br>"Although participants were selected from some facilities serving communities where EBSA had run programs, this did not guarantee their direct involvement in or awareness of the program itself. Additionally, including professionals from communities where EBSA was planning future programs allowed the study to capture prospective opinions on the program, offering insight into potential facilitators and barriers before implementation."<br><br>4) lack of clarity on the role of science: line 309 and 221 which similarly state that "confusing information comes from science". These two contradict with line 226 "few health professjonals made any reference to science". Does science resolve doubts or produces and ignites them? It also begs the question , if most of the experts are not refering science, why and how do they can be confused with scientific evidences on the topic?<br>Response:<br>We appreciate the reviewer's insightful observation regarding the apparent contradiction between the claim that science causes confusion (lines 309 and 221) and the statement that few professionals referenced science (line 226). To clarify, we have revised the paragraph to reflect that while science is often perceived as a source of contradictory information, most professionals were not directly engaging with primary scientific literature. Instead, their confusion appears to stem from institutional dietary guidelines, media narratives, and public health messaging rather than direct interpretation of research.<br>"Nurses and physicians often lacked confidence in their own nutritional knowledge and reported feeling uncertain when discussing dietary concepts. Rather than advising patients themselves, many preferred to refer them to dietitian, especially if they felt the patient required specific nutritional advice. They cited contradictory information from science, media, and government policies as key sources of confusion. Health professionals highlighted this inconsistency as a challenge for both themselves and their patients, contributing to public uncertainty about nutrition. A few health professionals attributed their lack of confidence in providing nutrition advice to insufficient training in both their formal education and continued professional development. Few participants referenced scientific evidence when justifying dietary recommendations. Therefore, while science was often cited as a source of conflicting information, it was rarely directly referenced by participants. Their confusion appeared to be from inconsistent public health messaging, institutional guidelines, and media influence rather than direct interaction with scientific literature."<br><br>5). Tha dat collecton (for this research) conducted in nov 2019 and the corresponding findings might not be of high relevance to adress the new dietary trends, including dietary transition initiatives.<br>Response:<br>We acknowledge the reviewer's concern regarding the relevance of findings given that

data collection occurred in 2019-2020. To address this, we have added a statement in the Limitations section clarifying that the findings reflect a specific period and that dietary discourse in South Africa may have evolved, particularly in response to pandemic-related changes in food access and health priorities. This temporal framing ensures that readers interpret the findings within the appropriate context.
"This study has a temporal limitation, as data collection took place between November 2019 and February 2020, meaning the findings capture a specific moment in time. Dietary discourse in South Africa may have evolved since then, particularly due to pandemic-related shifts in food access, nutrition policies, and public health priorities. While the study provides valuable insights into health professionals' perspectives, future research is needed to explore how these views may have changed with emerging dietary trends and transition initiatives."

6).inaccurate targeting of the sample population. The right targets should be nutritionists/dieticians. Not only that, they also should not be from private facilities. Generally, the paper has methodological and content gaps that need to be corrected.
Response:
We acknowledge the reviewer's concern regarding the sample selection and the inclusion of healthcare professionals beyond dietitians. While dietitians are indeed key informants in dietary counselling, our study intentionally included physicians and nurses to capture a broader perspective on how nutrition messages are conveyed within primary care. This approach aligns with community health models, which emphasize the role of generalist providers in under-resourced settings where dietitians are not always available. To address this, we have clarified this rationale we expanded the limitations section to acknowledge the potential for further specialized insights from a dietitian-focused study. This ensures that readers understand the study's scope while recognizing opportunities for future research.
"While dietitians play a central role in dietary counselling, this study included physicians and nurses to capture how nutrition messages are communicated in primary care, aligning with community health models for under-resourced settings. Future research focusing solely on dietitians could provide more specialized insights into LCHF counselling and implementation."

Reviewer #4

I am grateful for the opportunity to review this study, which already has the merit of bringing to light a little-known reality and featuring authors whose backgrounds make the work international.
The study is interesting, but I notice that the interviews were conducted more than 5 years ago, and the bibliography is a lot but very dated. I suggest motivating why it took so long to publish this work and what justifies using interviews from 5 years ago. Health issues and contexts evolve very quickly.
I wonder what is new about a paper that analyses data from 5 years earlier. I would therefore suggest specifying well what this study brings that is new.
Response:
We appreciate the reviewer's concern regarding the timing of data collection and the relevance of findings given the evolving nature of health issues. The study was conducted in 2019-2020, and while there has been a time gap in publication, the findings remain relevant as they provide critical insights into healthcare professionals' perspectives on dietary counseling and LCHF diets in under-resourced settings—issues that continue to be debated globally. To address this, we have added a justification in the Discussion section noting that while dietary discourse and healthcare priorities may have shifted since data collection, the core challenges identified—such as conflicting dietary guidance, limited nutrition training, and practical barriers to implementing dietary interventions—remain persistent in South Africa's primary care landscape. Additionally, we have acknowledged this as a study limitation while emphasizing that the findings offer a valuable historical perspective on professional attitudes toward LCHF diets at a critical moment in public health discourse.
Furthermore, to ensure that the study remains current, we have reviewed and updated the bibliography to include more recent references where applicable. We also clarify that this study brings new contributions by being one of the first to explore how a multidisciplinary group of healthcare providers, beyond just dietitians, perceive LCHF diets and community-based nutrition programs in South Africa. These insights help

inform future strategies for improving dietary counseling in primary care settings, particularly in under-resourced communities.

Furthermore, the bibliography should be updated to the last 10 years, preferably 5 years. Braun and Clarke 2006 can also be updated.
Response:
Thank you for your suggestion regarding the bibliography. We have reviewed and updated several references to ensure the inclusion of recent and relevant literature. Regarding Braun and Clarke (2006), we have intentionally retained this citation as it remains the foundational reference for the type of thematic analysis conducted in our study. Although more recent work by the authors exists, including their 2022 publication, the methodological approach we used aligns specifically with the principles outlined in their 2006 paper, rather than with reflexive thematic analysis as defined in later works. We have clarified this in the manuscript to maintain consistency and methodological rigour. However, we have included Braun and Clarke (2022) in the reference list to acknowledge and address the ongoing debate around the concept of data saturation in qualitative research, as discussed in our section on sampling and data sufficiency.
We have added the following statement to clarify our approach:
"The 2006 version of Braun and Clarke's thematic analysis was used. This version is characterised by its epistemological flexibility and openness to the use of codebooks, a feature that has since been abandoned by the authors in more recent iterations of this analytical method [45]."

Here are some more suggestions:
Title: Specify in the title the type of research: a qualitative research
Response:
We appreciate the reviewer's suggestion to specify the research type in the title. However, we believe that the current title, "Between support and skepticism: Health professionals' views on a nutrition education program promoting Low-Carbohydrate, High-fat diets in under-resourced South African communities" already implies a qualitative approach. The term "views" naturally indicates that the study explores perceptions and experiences, which aligns with qualitative research methodologies. Additionally, the abstract and methods section clearly define the study as a qualitative investigation, including details on in-depth interviews and thematic analysis. Given this, we feel that explicitly stating "qualitative research" in the title is unnecessary. That said, if the editorial team or reviewers strongly prefer including a methodological descriptor, we are open to refining the title to ensure clarity while maintaining its impact.

Keywords: Redefine keywords by adding for example perceptions, diet... and changing the order of the words: 6. thematic analysis; 3 low-carbohydrate, high-fat diet; 5 South Africa; 4 under-resourced communities; 2. health professionals. 1. perspectives on nutrition
Response:
We appreciate the reviewer's suggestion to refine the keywords for better alignment with the study's focus. These adjustments improve clarity by emphasizing perspectives and perceptions, restructuring the order for better readability, and ensuring the keywords accurately reflect the study's scope. We appreciate this valuable suggestion. Based on this feedback, we have revised the keywords as follows:
Revised Keywords:
perspectives on nutrition; healthcare professionals; low-carbohydrate, high-fat diet (LCHF); under-resourced communities; South Africa; thematic analysis; qualitative research.

Background:
The EBSA programme is not adequately described and the relevant literature does not support the use of this diet with solid evidence. I suggest that it should be better explained and recent and specific literature on this programme should be included. If it is a new programme, it should be explained in detail.
I would also explain in more detail studies 38-39-40 that are already specifically evaluating this programme.
Response:
We have added detail on EBSA's approach and placed it within the context of broader South African dietary guidelines in the Introduction.

	"Various nutritional education initiatives are active in low socio-economic areas of South Africa to address the burden of NCDs, with Eat Better South Africa (EBSA) being one prominent program [31]. EBSA promotes a low-carbohydrate high-fat (LCHF) dietary approach, focusing on nutrient-dense, minimally processed foods [31]. In contrast, the South African Food-Based Dietary Guidelines (FBDG) emphasize balanced diets, with staple whole grains, moderate fat intake, and a heavy focus on plant-based foods [32]."<br>We appreciate the request for further elaboration on previous evaluations of the EBSA program. We have now incorporated findings in the Introduction section from three studies that assessed the program's impact on participants' perceptions, metabolic health, and adherence challenges. These studies provide important context, demonstrating both the benefits and the practical difficulties of implementing LCHF diets in under-resourced communities. The revisions ensure that our discussion aligns with existing evidence and highlights the evolving understanding of EBSA's role in community nutrition interventions.<br>"A mixed methods evaluation of the EBSA program showed significant improvements in metabolic health markers among participants after six months, including weight loss, reduced waist circumference, and improved glycemic control, although adherence remained a challenge due to socioeconomic factors [40]. Additionally, an earlier study reported that program participants felt empowered through nutritional education, but required ongoing support to sustain dietary changes [38]. A qualitative community assessment exploring women's willingness to participate in an EBSA program highlighted both interest in and concerns about the affordability of LCHF diets, as well as challenges related to dietary transition [39]. These findings indicate that while EBSA has had measurable success in improving metabolic health, practical barriers to long-term adherence remain, necessitating better integration with healthcare professionals and community resources."<br><br>In this case, perhaps the research problem could be specified before the objective. For example, it could be: The results on the population of this dietetic education are positive (?), but the professionals still have different opinions. What then are the opinions of the professionals on the ESBA education programme?<br>The objectives of the study are different and do not facilitate consistency between objective, data collection instrument (interview) and analysis. I would propose to select only one objective, the first one, at least as a macro-objective. The others could become sub-objectives.<br>Response:<br>We appreciate the reviewer's suggestion to refine the research problem and objectives for greater clarity and consistency. We have clarified the primary objective while integrating the other objectives within the same framework to ensure alignment between the study's aim, data collection, and analysis. These revisions strengthen the connection between the research problem, objectives, and methodological approach, ensuring a clearer focus on how healthcare professionals perceive and engage with EBSA's dietary recommendations.<br>"While some health professionals may support LCHF diets for certain individuals or specific health conditions, others may have reservations based on concerns about long-term health effects, individual variability, sustainability, adherence, and potential nutrient deficiencies [41,42]. Although the EBSA program has shown positive outcomes in improving dietary behaviours and metabolic health among participants, healthcare professionals hold varying opinions on its dietary approach, highlighting the need to explore their perspectives in greater depth. This article aims to explore healthcare professionals' perspectives on the EBSA program, particularly focusing on the effectiveness and sustainability of the LCHF diet in managing NCDs within South Africa. While the primary objective is to examine how healthcare professionals perceive the effectiveness of the EBSA program and its dietary recommendations, this study also investigates the barriers and facilitators that influence their willingness to support or integrate EBSA dietary guidance into their practice. Furthermore, it assesses how professional training, healthcare system constraints, and exposure to evolving nutrition science shape the attitudes of these healthcare professionals toward LCHF diets and nutrition education. It examines the alignment and potential conflicts between the nutritional advice given by professionals and the guidelines promoted by EBSA, identifying gaps in nutrition education and training. This article also explores the broader public health implications of integrating such dietary recommendations and seeks to provide insights for refining the EBSA program to better meet the health

needs of South Africa's diverse population."

Material and methods
Since the setting is very detailed, insert a sub-paragraph with 'setting'. Line 116
Response:
We have added a sub-paragraph titled Setting, which describes the context of the healthcare facilities involved in the study. This helps to better frame the environment in which data collection took place, as follows:

Participants. As a rule, participants in the study are also given a consent form for the use of the data. With this consent, anonymised and aggregated data can be disseminated. Has a particular choice been made in this respect?
Response:
We appreciate the reviewer's concern regarding participant consent for data sharing. As outlined in the Data Availability section, participants did not specifically provide consent for their transcripts to be shared publicly. The consent statement, approved by the Human Research Ethics Committee of the University of Cape Town, did not include a provision for making the data publicly available. Therefore, we do not have participant consent to share these data openly. However, requests for anonymized data can be made to the Principal Investigator, Georgina Pujol-Busquets (georgipbg@uoc.edu), in accordance with ethical guidelines. This ensures that data confidentiality and participant agreements are upheld.

Inclusion criteria: 'a community where EBSA is planning to run future programmes'. Why?
Response:
The inclusion criterion regarding "communities where EBSA plans to run future programs" was included to explore prospective opinions of healthcare professionals who may soon work with the EBSA program. This forward-looking approach provides valuable insight into how the program could be received, adapted, or challenged in the future. This has been added in the Participants section.
"Additionally, including professionals from communities where EBSA was planning future programs allowed the study to capture prospective opinions on the program, offering insight into potential facilitators and barriers before implementation."

Procedures
Line 142: There is the problem of the year of the interviews, but it has already been addressed in the introduction.
Response:
We have clarified in the text that the interviews were conducted between November 2019 and February 2020, and that this timing is acknowledged as a limitation in understanding any shifts in dietary discourse.
"This study has a temporal limitation, as data collection took place between November 2019 and February 2020, meaning the findings capture a specific moment in time. Dietary discourse in South Africa may have evolved since then, particularly due to pandemic-related shifts in food access, nutrition policies, and public health priorities. While the study provides valuable insights into health professionals' perspectives, future research is needed to explore how these views may have changed with emerging dietary trends and transition initiatives."

Line 144: I have some concerns about the sample and saturation. I prefer to talk about participants, rather than sampling, which is more typical of quantitative research. With respect to data saturation, opinions are still very diverse today. While I do not agree with what has been written, I would not change it as you have cited the literature. (I would add, however, that using thematic analysis by Braun and Clarke, these authors have written several articles on data saturation, always in a reflexive manner).
Response:
We appreciate the reviewer's concern regarding the terminology of sampling in qualitative research and the debate around data saturation in reflexive thematic analysis. In response, we have adjusted the wording in the Procedures section to emphasize that our study follows qualitative research principles that prioritize depth of insight. Additionally, we have acknowledged Braun and Clarke's position on data saturation, which views thematic development as an interpretive process rather than a point of theoretical exhaustion. This ensures that the discussion of saturation is aligned

	with the methodological framework used in the study.<br>“The IDIs were conducted between 29th of November and 21st February 2020 and lasted, on average, 30 minutes. The number of participants aligns with qualitative research recommendations, which suggest that meaningful insights can be drawn from smaller, information-rich samples [44]. According to the literature, the first five to six IDIs generally produce the majority of new data, and the majority of concepts are identified within the first 10 interviews. As such, new participants were interviewed until saturation was reached [4...
**Additional Information:**	
**Question**	**Response**
**Financial Disclosure**<br><br>Enter a financial disclosure statement that describes the sources of funding for the work included in this submission. Review the submission guidelines for detailed requirements. View published research articles from *PLOS ONE* for specific examples.<br><br>This statement is required for submission and **will appear in the published article** if the submission is accepted. Please make sure it is accurate.<br><br>**Funded studies**<br>Enter a statement with the following details:<br>• Initials of the authors who received each award<br>• Grant numbers awarded to each author<br>• The full name of each funder<br>• URL of each funder website<br>• Did the sponsors or funders play any role in the study design, data collection and analysis, decision to publish, or preparation of the manuscript?<br><br>Did you receive funding for this work?	The author(s) received no specific funding for this work.
**Competing Interests**<br><br>Use the instructions below to enter a competing interest statement for this submission. On behalf of all authors, disclose any competing interests that could be perceived to bias this work—acknowledging all financial support and any other relevant financial or non-financial competing interests.	The authors have declared that no competing interests exist.

This statement is required for submission and **will appear in the published article** if the submission is accepted. Please make sure it is accurate and that any funding sources listed in your Funding Information later in the submission form are also declared in your Financial Disclosure statement.<br><br>View published research articles from *PLOS ONE* for specific examples.<br><br>**NO authors have competing interests**<br><br>Enter: *The authors have declared that no competing interests exist.*<br><br>**Authors with competing interests**<br><br>Enter competing interest details beginning with this statement:<br><br>*I have read the journal's policy and the authors of this manuscript have the following competing interests: [insert competing interests here]*<br><br>* typeset	
**Ethics Statement**<br><br>Enter an ethics statement for this submission. This statement is required if the study involved:<br><br>• Human participants<br>• Human specimens or tissue<br>• Vertebrate animals or cephalopods<br>• Vertebrate embryos or tissues<br>• Field research<br><br>Write "N/A" if the submission does not require an ethics statement.<br><br>General guidance is provided below. Consult the submission guidelines for detailed instructions. **Make sure that all information entered here is included in the**	The Human Research Ethics Committee (HREC REF 512/2019) of the University of Cape Town approved the study protocol and informed consent procedures. Written informed consent was obtained from all participants

**Methods section of the manuscript.**<br><br>**Format for specific study types**<br><br>**Human Subject Research (involving human participants and/or tissue)**<br>• Give the name of the institutional review board or ethics committee that approved the study<br>• Include the approval number and/or a statement indicating approval of this research<br>• Indicate the form of consent obtained (written/oral) or the reason that consent was not obtained (e.g. the data were analyzed anonymously)<br><br>**Animal Research (involving vertebrate animals, embryos or tissues)**<br>• Provide the name of the Institutional Animal Care and Use Committee (IACUC) or other relevant ethics board that reviewed the study protocol, and indicate whether they approved this research or granted a formal waiver of ethical approval<br>• Include an approval number if one was obtained<br>• If the study involved *non-human primates*, add *additional details* about animal welfare and steps taken to ameliorate suffering<br>• If anesthesia, euthanasia, or any kind of animal sacrifice is part of the study, include briefly which substances and/or methods were applied<br><br>**Field Research**<br><br>Include the following details if this study involves the collection of plant, animal, or other materials from a natural setting:<br>• Field permit number<br>• Name of the institution or relevant body that granted permission	
**Data Availability**<br><br>Authors are required to make all data underlying the findings described fully available, without restriction, and from the time of publication. PLOS allows rare exceptions to address legal and ethical concerns. See the PLOS Data Policy and FAQ for detailed information.	No - some restrictions will apply

A Data Availability Statement describing where the data can be found is required at submission. Your answers to this question constitute the Data Availability Statement and **will be published in the article**, if accepted.<br><br>**Important:** Stating 'data available on request from the author' is not sufficient. If your data are only available upon request, select 'No' for the first question and explain your exceptional situation in the text box.<br><br>Do the authors confirm that all data underlying the findings described in their manuscript are fully available without restriction?	
**Describe where the data may be found in full sentences. If you are copying our sample text, replace any instances of XXX with the appropriate details.**<br><br>• If the data are **held or will be held in a public repository**, include URLs, accession numbers or DOIs. If this information will only be available after acceptance, indicate this by ticking the box below. For example: *All XXX files are available from the XXX database (accession number(s) XXX, XXX.).*<br>• If the data are all contained **within the manuscript and/or Supporting Information files**, enter the following: *All relevant data are within the manuscript and its Supporting Information files.*<br>• If neither of these applies but you are able to provide **details of access elsewhere**, with or without limitations, please do so. For example:<br><br>*Data cannot be shared publicly because of [XXX]. Data are available from the XXX Institutional Data Access / Ethics Committee (contact via XXX) for researchers who meet the criteria for access to confidential data.*<br><br>*The data underlying the results*	The full transcripts and raw data from this study cannot be shared publicly due to ethical and legal restrictions. The research involved interviews with healthcare professionals discussing potentially sensitive information related to their workplace, patients, and professional practices. The approved protocol, which was reviewed by the Human Research Ethics Committee of the University of Cape Town (HREC REF 512/2019), did not include provisions for public data sharing. Participants consented to participate in the study under the condition that their full transcripts and identifying information would remain confidential and would not be made publicly available. However, researchers who meet the criteria for access to confidential data may request access to de-identified datasets by contacting the Human Research Ethics Committee of the University of Cape Town (Hub-PhysiologicalSciences@uct.ac.za) or the P.I. Georgina Pujol-Busquets (georgipbg@uoc.edu).

presented in the study are available from (include the name of the third party and contact information or URL).<br>• This text is appropriate if the data are owned by a third party and authors do not have permission to share the data.<br><br>* typeset	
Additional data availability information:	

Powered by Editorial Manager® and ProduXion Manager® from Aries Systems Corporation

AHTISHAM YOUNAS
Faculty of Nursing
Memorial University of Newfoundland
St. John's, NL A1C 5S7
P.O. Box 4200
CANADA

Dear Dr. Essouma,

On behalf of my coauthors, I am resubmitting the manuscript entitled "Between support and scepticism: Health professionals' perceptions of a nutrition education program promoting low-carbohydrate, high-fat diets in under-resourced South African communities" for consideration as a research article in *PLOS One*.

We would like to thank you for your feedback and for the opportunity to submit a revised version of the manuscript. As requested, we have revised the manuscript based on your and the reviewers' suggestions, and we believe that these revisions have improved the overall quality of the manuscript.

This study explores healthcare professionals' perspectives on the implementation of Low-Carbohydrate, High-Fat (LCHF) dietary interventions in under-resourced South African communities, focusing on the Eat Better South Africa (EBSA) program. To our knowledge, this is the first qualitative study examining healthcare practitioners' views on LCHF diets and related community-based initiatives in South Africa. Our findings provide critical insights into the alignment and discord between healthcare professionals' dietary recommendations and emerging community-based nutrition interventions, with implications for addressing non-communicable diseases (NCDs) in low-income settings.

This qualitative study is part of Dr Georgina Pujol-Busquets's PhD dissertation, which encompasses four research studies. The thesis is available in the open-access institutional repository of the University of Cape Town (UCT) under the title, "Evaluation of a nutrition and health education program on components of metabolic health in women from under-resourced South African communities." The following three studies from this project have already been published elsewhere and provide valuable insights into the topic under investigation:

1. Pujol-Busquets G, Smith J, Larmuth K, Fàbregues S, Bach-Faig Exploring the perceptions of women from under-resourced South African communities about participating in a low-carbohydrate high-fat nutrition and health education program: A qualitative focus group study. *Nutrients*. 2020; 12(4):894. https://doi.org/10.3390/nu12040894
2. Pujol-Busquets G, Smith J, Fàbregues S, Bach-Faig A, Larmuth K. Community assessment for a low-carbohydrate nutrition education program in South Africa. *Nutrients*. 2023; 15(1):67. https://doi.org/10.3390/nu15010067
3. Pujol-Busquets G, Smith J, Fàbregues S, Bach-Faig A, Larmuth K. Mixed methods evaluation of a low-carbohydrate high-fat nutrition education program for women from underserved communities in South Africa. *Appetite*. 2024;204:107725. https://10.1016/j.appet.2024.107725

This study builds on previous research on LCHF dietary interventions, particularly in resource-constrained environments, but adds a novel perspective by examining the views of healthcare practitioners. This work contributes to the broader literature on nutrition education, health equity, and the barriers to adopting non-traditional dietary approaches in disadvantaged communities.

The full transcripts and raw data from this study cannot be shared publicly due to ethical and legal restrictions. The research involved interviews with healthcare professionals discussing potentially sensitive information related to their workplace, patients, and professional practices. The approved protocol, which was reviewed by the Human Research Ethics Committee of the University of Cape Town (HREC REF 512/2019), did not include provisions for public data sharing. Participants consented to participate in the study under the condition that their full transcripts and identifying information would remain confidential and would not be made publicly available. However, researchers who meet the criteria for access to confidential data may request access to de-identified datasets by contacting the Human Research Ethics Committee of the University of Cape Town (Hub-PhysiologicalSciences@uct.ac.za) or the P.I. Georgina Pujol-Busquets (georgipbg@uoc.edu).

This study received funding from The Noakes Foundation and The National Research Foundation of South Africa. The funders had no role in study design, data collection and analysis, decision to publish, or preparation of the manuscript.

We confirm that this manuscript has not been previously published and is not under consideration elsewhere. We have had no prior interactions with *PLOS One* regarding this manuscript. I hereby confirm that all authors listed on the manuscript accept full responsibility for all aspects of the work described.

Thank you for considering this resubmission. We believe our study aligns with the scope and mission of PLOS One, and we look forward to the possibility of contributing to your esteemed journal.

Yours sincerely,
Ahtisham Younas, PhD

Manuscript
Click here to access/download;Manuscript;Manuscript.docx

Between support and scepticism: Health professionals' perceptions of a nutrition education program promoting low-carbohydrate, high-fat diets in under-resourced South African communities

Georgina Pujol-Busquets[1,2,*], Kate Larmuth[1,3,4] , Christopher C Webster[1,3,4], James Smith[1,3,4], Ahtisham Younas[5,*], Sergi Fàbregues[2,6] and Anna Bach-Faig[2]

[1]Division of Physiological Sciences, Department of Human Biology, University of Cape Town, South Africa.

[2]NUTRALiSS Research Group, Faculty of Health Sciences, Universitat Oberta de Catalunya (UOC), Barcelona, Spain.

[3]International Federation of Sports Medicine (FIMS) Collaborative Centre of Sports Medicine, HPALS, University of Cape Town, South Africa.

[4]Research Center for Health through Physical Activity, Lifestyle and Sport, Division of Physiology, Department of Human Biology, Faculty of Health Sciences, University of Cape Town, Cape Town, South Africa.

[5]Faculty of Nursing, Memorial University of Newfoundland, St. John's, NL, Canada.

[6]Department of Psychology and Education, Universitat Oberta de Catalunya (UOC), Barcelona, Spain.

*Corresponding author: ay6133@mun.ca

Abstract

The burden of non-communicable diseases (NCDs) continues to rise, emphasizing the need for effective dietary interventions. Programs such as *Eat Better South Africa (EBSA)* advocate for low-carbohydrate high-fat (LCHF) dietary choices, especially in disadvantaged communities. However, the adoption of such approaches among healthcare professionals remains contentious. This qualitative study explores healthcare professionals' perspectives on nutrition and the EBSA program, drawing on 16 in-depth individual interviews with physicians, nurses, and dietitians from False Bay Hospital, Groote Schuur Hospital, and a primary health clinic in Hout Bay, Western Cape, South Africa. Thematic analysis of the interviews revealed four main findings. Healthcare professionals lacked confidence in their nutritional knowledge, and while many were familiar with the LCHF diet, opinions varied regarding its sustainability and health implications. Concerns were raised about the high fat content and affordability of LCHF foods. Professionals acknowledged the value of group support in behaviour change, as promoted by EBSA, but expressed reservations about its strong emphasis on LCHF diets. Key challenges identified for patients included poverty, cultural beliefs, limited education, and access to nutritious foods. The findings highlight a reliance on traditional dietary advice, with uncertainties about the feasibility and affordability of LCHF diets. These findings offer novel insights into the complexities of implementing community-based dietary interventions in South Africa, with implications for policy and practice.

Keywords

perspectives on nutrition; healthcare professionals; low-carbohydrate, high-fat diet (LCHF); under-resourced communities; South Africa; thematic analysis; qualitative research.

Introduction

South Africa's public health system faces major challenges, including long wait times, resource shortages, poor management, inadequate infection control, and workforce deficits—exacerbated by inequitable resource distribution, rapid urbanization, and the dual burden of infectious and non-communicable diseases (NCDs) [1-9]. Within this strained system, the healthcare workforce is crucial to advancing universal healthcare goals, particularly in addressing South Africa's complex disease challenges, both infectious and non-communicable [2,9-12]. Despite its critical role in health, nutrition remains poorly integrated into healthcare training, leading to inconsistent advice and under prepared professionals. Yet globally, nutrition is increasingly recognised as central to disease prevention and management, with dietary interventions often matching or exceeding pharmacotherapy in effectiveness and cost-efficiency [13-16]. This is a global concern, as nutrition profoundly influences health outcomes [17-20]. International efforts are bridging gaps in nutrition education, supported by evidence of the effectiveness of dietary and physical activity interventions, which often rival pharmacological treatments in efficacy, safety, and cost-effectiveness [13,16, 21- 24]. Studies consistently show that nutrition interventions reduce the incidence and severity of diet-related non-communicable disease (NCDs), improve patient outcomes, reduce healthcare costs, and enhance the well-being of healthcare professionals, especially in acute care settings [25–30].

Various nutritional education initiatives are active in low socio-economic areas of South Africa to address the burden of NCDs, with *Eat Better South Africa (EBSA)* being one prominent program [31]. EBSA promotes a low-carbohydrate high-fat (LCHF) dietary approach, with an emphasis on nutrient-dense, minimally processed foods [31]. The LCHF approach contrasts with the South African Food-Based Dietary Guidelines, which emphasise balanced diets with staple whole grains, moderate fat intake, and a heavy focus on plant-based foods [32]. EBSA aims to empower individuals, particularly women, through dietary education, believing in the transformative power of education on health behaviours and overall well-being [31]. LCHF

diets are supported by some clinicians and professional societies as effective treatments for diabetes and obesity management [33]. However, this approach is contentious, with conflicting findings polarising expert opinion and potentially causing confusion among healthcare professionals and patients [34]. Despite these controversies, carbohydrate restriction for managing type 2 diabetes is supported by international consensus guidelines [35,36]. International consensus guidelines cautiously support carbohydrate restriction for type 2 diabetes [35,36], yet historical precedents—such as William Banting's 19th-century dietary polemics—highlight the enduring controversy surrounding this approach [37].

In focus group discussions involving previous participants of EBSA programs, participants noted differences between the dietary guidance provided by healthcare professionals, which aligns with a Western Standard diet in their communities, and the EBSA recommendations [38-40]. Some healthcare professionals who were initially sceptical of the EBSA LCHF diet shifted towards supporting it, as they witnessed positive health outcomes in their patients. A mixed methods evaluation of the EBSA program showed significant improvements in metabolic health markers among participants after six months, including weight loss, reduced waist circumference, and improved glycaemic control, although adherence remained a challenge due to socioeconomic factors [40]. Additionally, an earlier study reported that program participants felt empowered through nutritional education, but required ongoing support to sustain dietary changes [38]. A qualitative community assessment exploring women's willingness to participate in an EBSA program highlighted both interest in and concerns about the affordability of LCHF diets, as well as challenges related to dietary transition [39]. These findings indicate that while EBSA has had measurable success in improving metabolic health, practical barriers to long-term adherence remain, necessitating better integration with healthcare professionals and community resources.

While some health professionals may support LCHF diets for certain individuals or specific health conditions, others may have reservations based on concerns about long-term health effects, individual variability, sustainability, adherence, and potential nutrient deficiencies [41,42]. Although the EBSA program has shown positive outcomes in improving dietary behaviours and metabolic health among participants, healthcare professionals hold varying opinions on its dietary approach, highlighting the need to explore their perspectives in greater depth. This article aims to explore healthcare professionals' perspectives on the EBSA program, particularly focusing on the effectiveness and sustainability of the LCHF diet in managing NCDs within South Africa. While the primary objective is to examine how healthcare professionals perceive the effectiveness of the EBSA program and its dietary recommendations, this study also investigates the barriers and facilitators that influence their willingness to support or integrate EBSA dietary guidance into their practice. Furthermore, it assesses how professional training, healthcare system constraints, and exposure to evolving nutrition science shape the attitudes of these healthcare professionals toward LCHF diets and nutrition education. It examines the alignment and potential conflicts between the nutritional advice given by professionals and the guidelines promoted by EBSA, identifying gaps in nutrition education and training. This article also explores the broader public health implications of integrating such dietary recommendations and seeks to provide insights for refining the EBSA program to better meet the health needs of South Africa's diverse population.

Materials and methods

This is a qualitative study using in-depth individual interviews (IDIs) with health professionals from health care facilities that would service current and future EBSA programs. The research team included experts in qualitative research and nutrition science.

Setting

Participants were selected from two tertiary institutions, namely False Bay Hospital (Fish Hoek, Cape Town) and Groote Schuur Hospital (Observatory, Cape Town), and a primary health clinic in Hout Bay (Hout Bay, Cape Town). False Bay Hospital is a public hospital that was opened in 1965 in Fish Hoek, a town approximately 30 kilometres south of Cape Town. The hospital has approximately 80 beds, and its facilities include a 24-Hour Emergency Unit. It is the only district hospital in the South Peninsula Health District of the Metro Region and serves many communities, including Simonstown, Masiphumelele, Ocean View, Clovelly, and many other surrounding areas. Groote Schuur Hospital is one of Cape Town's largest government-funded academic hospitals and was officially opened in 1938. The hospital has almost 900 beds and it is renowned as the hospital where the world's first human-to-human heart transplant was performed in 1967 by Christiaan Barnard and his team [43]. The Hout Bay health clinic non-profit organization works mostly in an informal settlement called Imizamo Yethu and provides nutrition and health education to the community. Hout Bay is a seaside town in the Western Cape Province situated in the Cape Peninsula, 20 kilometres south of Cape Town.

Participants

To be eligible for the study, participants had to be health professionals; work in and see patients from the communities where EBSA has conducted a nutrition education program or a community where EBSA is planning to run future programs; and be able to understand and speak English. Community health clinics were contacted by email or phone to identify potential participants. A researcher explained the study to interested participants, either via phone or at a scheduled meeting, and if they met the inclusion criteria, they were provided with a study information sheet and the informed consent form. An interview visit was then scheduled at a convenient time and place for the participants. The participants met individually with a researcher for approximately thirty minutes to an hour in a private venue within the

participating health facilities, ensuring both privacy and accessibility, where they signed the informed consent form and were interviewed in English. Although participants were selected from some facilities serving communities where EBSA had run programs, this did not guarantee their direct involvement in or awareness of the program itself. Additionally, including professionals from communities where EBSA was planning future programs allowed the study to capture prospective opinions on the program, offering insight into potential facilitators and barriers before implementation.

Procedures

The IDIs were conducted between 29th of November and 21st February 2020 and lasted, on average, 30 minutes. The number of participants is consistent with recommendations in the qualitative research literature, which suggests that meaningful insights can be gained from smaller, information-rich samples [44]. According to this literature, the first five to six IDIs generally produce the majority of new data, and the majority of concepts are identified within the first 10 interviews. As such, new participants were interviewed until saturation was reached [44]. However, as outlined by Braun and Clarke, we acknowledge that the concept of data saturation is debated, with an emphasis on depth of interpretation rather than a fixed threshold for thematic exhaustion [45]. Participants were interviewed on a one-on-one basis to explore their understanding and perceptions of chronic disease, nutrition, LCHF diets, and the EBSA program. A semi structured interview guide (see SI1 File) was used which included both open-ended and probing questions as a guide for the discussion, although questions were not necessarily asked verbatim. Probing questions were asked only if the subjects had not already been covered.

The interview guide was developed based on a review of the literature on healthcare professionals' perceptions of dietary interventions, particularly low-carbohydrate diets. It was piloted with two health professionals (not included in the final sample) to ensure the relevance,

clarity, and cultural appropriateness of the questions. The final guide followed a semi-structured format, allowing for follow-up questions and probing to explore emerging topics in depth. It covered the following key areas: healthcare providers' perceptions of their role in community health; major health concerns identified in their communities; views on NCDs such as obesity, diabetes, and cardiovascular disease; the type of dietary advice offered at their healthcare facilities; perspectives on the role of nutrition in chronic disease prevention and treatment; their understanding of local dietary patterns; and their awareness and impressions of the EBSA programme and the LCHF diet, including views on the feasibility of implementing such interventions in their communities.

All interviews were audio-recorded, with the permission of the participants. The interviews were conducted by GPB and saved in a password-protected folder. The recordings were transcribed by a professional transcription service (Cyber Transcription, South Africa) and the accuracy and editing of the transcriptions were double-checked by an external qualitative researcher for rigour and appropriateness. Each transcribed interview was compared with the corresponding audio to ensure that the transcription had been done in context and captured the interview as accurately as possible.

Data analysis

Braun and Clarke's approach to thematic analysis [46] was followed using NVivo 12 software (QSR International, Melbourne, Australia). The 2006 version of Braun and Clarke's thematic analysis was used. This version is characterised by its epistemological flexibility and openness to the use of codebooks, a feature that has since been abandoned by the authors in more recent iterations of this analytical method [45].

The analysis began with CCW and GPB reading the transcripts several times, summarising them, and making notes on ideas and potential codes. These two researchers then each inductively developed a codebook based on the summaries and notes generated by reading

the transcripts in the previous step. Then, they discussed their individual codebooks, merged them into one codebook, and independently coded three randomly selected interviews using separate NVivo projects. CCW and GPB then met again to compare their respective coding, and discuss and resolve disagreements by consensus. The content and structure of the final codebook was finalised through discussions involving ABF, SF, and KL, to ensure consistency and clarity in the coding framework. Once agreement on the codebook was reached among the members of the research team, the remaining interviews were divided into two groups of eight, with each group assigned to GPB or CCW. GPB and CCW held regular meetings to discuss their interpretations, resolve discrepancies, and critically engage with the data. These collaborative discussions allowed for the refinement of the coding framework and the development of sub-themes and themes, ensuring consistency in analytical decisions. The themes developed were then visually organised by GPB using thematic maps, which supported further analysis and interpretation. During and after the interviews, the research team systematically recorded observations and took field notes, including contextual insights and paraverbal cues such as tone, pauses, and emphasis. Although this data was not formally coded, it was used in the process of interpreting and understanding the participants' narratives and in the development of the themes.

The following step involved naming the themes and defining the main concepts of each theme, resulting in a total of four themes. These themes were shared with ABF, SF, KL and JS to confirm their relevance to the research questions. The fact that, as explained above, two researchers (CCW and GPB) were involved in the analysis of the data, and that the different steps of the analysis were reviewed by the other members of the research team, helped to ensure that all the themes were identified and that the data was interpreted in a consistent way, avoiding the possibility of a premature closure of the analysis, limiting the potential effect of bias in the adoption of a singular point of view, and promoting the overall quality of the

analysis. We did not use member checking for practical and methodological considerations. Logistically, time and resource constraints prevented us from following up with participants. Methodologically, we adopted an approach that recognises the dynamic and contextual nature of participants' perceptions, which may shift over time and are not necessarily fixed or unique. Participant feedback is shaped by the circumstances in which it is elicited and does not constitute a definitive test of validity [47].

Finally, the last step, carried out mainly by GPB, consisted of producing a written report of the study findings. Quotes from the qualitative data were selected to illustrate the themes identified in the data, ensuring that each captured the essence of the theme. The study adhered to the Consolidated Criteria for Reporting Qualitative Research (COREQ) guidelines to ensure that the methods and findings were reported in a transparent and rigorous manner [48].

Ethical considerations

The Human Research Ethics Committee (HREC REF 512/2019) of the University of Cape Town approved the study protocol and informed consent procedures. All procedures were performed in accordance with the 1964 Helsinki Declaration and its later amendments. Informed consent was obtained from all individual participants involved in the study. The health facilities of the study consented to the interviews being conducted at their practices.

Results

The participants recruited for the study were from a variety of general practices and socio-economic localities. A total of 16 healthcare professionals participated in the study, including 7 medical physicians, 7 nurses, and 2 dietitians. To ensure transparency and traceability in the reporting of quotes, each participant was assigned a code reflecting their profession and participant number (e.g., MPh.1 for Male Physician 1, FN.1 for Female Nurse 1, FD.1 for Female Dietitian 1). The average age of the participants was 37.1 ± 9.8 years. A

majority of participants (n = 13) worked only in the public sector, two participants practiced in both the public and private sectors, and one participant practiced only in the private sector. Four themes (Table 1) were developed from the analysis.

Table 1. Themes developed from IDIs with health professionals.

Theme	Sub-themes	Definition	Quotes
**Theme 1. Foundations of dietary understanding**	1.1. Knowledge of nutritional guidelines<br>1.2. Confidence in nutritional counselling	Refers to their understanding of dietary principles, nutritional requirements, and the role of food in promoting health and preventing disease among the population. This knowledge encompasses various aspects including nutritional guidelines, assessment, dietary counselling and nutrition education.	'Studies have shown that with diabetes even if you increase the medication or you optimize the medication, at the end of the day it's the diet that kind of makes or breaks the disease' (FPh.2).<br>'We basically follow the protocols of the National Health Department (…) but it's outdated and it's not up to speed with what's really happening' (MPh.4).<br>'I definitely don't say you need to cut it out completely [sugar] (…) I understand we all do enjoy sweet things here and there' (FD.1).
**Theme 2. LCHF: Controversies and beliefs**	2.1. Awareness of LCHF diets<br>2.2. Concerns over high-fat content	Understanding of health professionals around the recommendations of LCHF diets which depend on their training, experience and personal beliefs.	'I was on [a LCHF diet], but because I didn't have a lot of support (…) I just left it then, but it's actually a good diet (…) I didn't feel so tired' (FN.5).<br>'If you eat high fat things your cholesterol is going to go up.' (FPh.3).<br>'I don't advocate a LCHF diet but I'm supportive of what my patients want to try and then I monitor them closely. I watch their cholesterols, their triglycerides, their blood pressures.' (MPh.2).<br>'I wouldn't encourage it [LCHF diet], but if a patient really wanted to do it and ask for my advice, I would try to guide them on it yes, for sure' (FD.2).
**Theme 3. EBSA's role in community nutrition**	3.1. Perceptions of group support<br>3.2. Uncertainty about program sustainability	Health professionals' understanding and perceptions of the EBSA program promoting LCHF within under-resourced communities which encompass EBSA's group support approach, diet recommendations and program's sustainability.	'When you're in a group, then there's more support from one another.' (FN.3).<br>'I think it's a great initiative [EBSA], very necessary because if it's successful, it will definitely reduce the disease burden in our community and that impacts on everything' (FPh.3).<br>'If the people don't have the funding, they're not going to be able to buy it [EBSA's recommended food]' (FPh.7).

**Theme 4: Understanding socioeconomic determinants of health**	4.1. Affordability and food access<br>4.2. Cultural and gender norms shaping diet	Health professionals' understanding of the barriers that under-resourced patients face in accessing healthcare and maintaining good health. Their views on these barriers are informed by their direct interactions with patients, as well as their broader understanding of social determinants of health.	'"Beggars can't be choosers". While they [people from low-income communities] might know that what they're eating is not good for them, they don't have the means to put that into place' (MPh.1).<br>'It's a very big cultural thing to be big [body size]. There's a stigma around it if you're skinny and you're losing weight, you are becoming sick' (FPh.6).<br>'I think in certain cultures, women don't have a lot of say in what gets done, so if the man comes home and he says "he wants this sort of meal" that's what she must prepare.' (FPh.3).

LCHF: Low carbohydrate high fat; EBSA: Eat Better South Africa; M: Male; F: Female; Ph: Physician; N: Nurse; D: Dietitian.

Theme 1: Foundations of dietary understanding

Health professionals recognised that they are not that well-informed about nutrition and its role in disease prevention. They felt constrained by time, stating that while nutritional advice is important for disease prevention, they struggle to incorporate it into clinical consultation. However, they acknowledged that nutrition plays a crucial role in promoting healthy-diet behaviours and preventing diet-related chronic diseases. There was a general sense of confusion regarding nutrition, as participants described encountering contradictory dietary information from different sources, making it difficult to provide clear guidance to patients. As a result, they tend to use many "catchphrases" and their own statements of beliefs. This is effectively based on what has traditionally been promoted as healthy behaviour: small portions, reducing red meat, eating fruit and vegetables, drinking water, reducing dietary fats, avoiding unsaturated fats (for instance, removing fat and skin from chickens) and practising physical activity.

Nurses and physicians often lacked confidence in their own nutritional knowledge and reported feeling uncertain when discussing dietary concepts. Rather than advising patients

themselves, many preferred to refer them to dietitian, especially if they felt that the patient required specific nutritional advice. They cited contradictory information from science, media, and government policies as key sources of confusion. Health professionals highlighted this inconsistency as a challenge for both themselves and their patients, contributing to public uncertainty about nutrition. A few health professionals attributed their lack of confidence in providing nutrition advice to insufficient training in both their formal education and continued professional development. Few participants referenced scientific evidence when justifying dietary recommendations. Therefore, while science was often cited as a source of conflicting information, it was rarely directly referenced by participants. Their confusion appeared to be from inconsistent public health messaging, institutional guidelines, and media influence rather than direct interaction with scientific literature.

Despite not keeping up with new developments in nutrition science, participants agreed that more clinical trials are needed to reassess current dietary guidelines and challenge dietary stigmas, particularly regarding dietary fat and cholesterol levels. Dietary guidance often relied on simplified "catchphrases" (e.g., "balanced diet," "portion control", "sustainable diet") rather than evidence-based specifics. While dietitians emphasized moderation, physicians and nurses were more likely to label foods as "good" or "bad," focusing on reducing sugar, salt, and processed foods. Notably, healthcare professionals rarely referenced scientific literature directly, attributing confusion to conflicting public health messaging rather than engagement with primary research. These ‘catchphrases’ can be defined as statements that are repeated without any justification or attempt to explain them, as if they were obvious. Another notable pattern in participant responses was the use of absolutes when discussing dietary beliefs, often presenting certain nutritional claims as self-evident truths. Phrases such as "obviously," "we all know," and "we have known for years" indicated that health professionals regarded certain dietary messages as unquestionable.

There was also some consistent advice that was tailored to specific conditions. For instance, low salt for hypertension and portion control and exercise for obesity. The professionals agreed indirectly that healthy food improves immunity; therefore, human immunodeficiency virus (HIV) patients should be encouraged to eat healthy foods because their immune system is compromised. In general, the cohort thought that good advice would be to promote physical activity, cut out simple sugar, stop or reduce smoking, lower salt intake, reduce alcohol, not eat spicy food, remove the skin off chicken, remove fat from red meat, and zero sugar sodas instead of regular ones. Although there were differences in how dietitians, physicians, and nurses approached nutrition advice, there was a shared tendency to frame dietary guidance in practical rather than prescriptive terms. While dietitians emphasized moderation and portion control, physicians and nurses were more likely to label specific foods as problematic. Foods considered detrimental to health included those high in sugar and fat, convenience foods, large portions, starchy foods, sugary drinks, processed snacks, fast foods, and overly salty foods. Examples of such foods were described as: instant or convenience food, inexpensive and easy to access, served in large portions, high-starch content, sugary drinks, 'chippies and sweeties', junk food, high-calorie food, salty food, 'braai-ed' [cooked on a barbecue] meat and more. Healthy alternatives were described as vegetables, fruits, small portions, oats, three balanced meals a day, healthy snacks (such as apples or whole-wheat crackers), and avoiding processed foods and sauces. Some professionals also recommended low-fat options, choosing chicken over red meat and reducing carbohydrate intake. Some mentioned that they advised their patients to eat food in their natural state, not too overprocessed, in moderation and avoid sauces. In general, the advice on practicing physical activity seems to be non-specific and is used when explaining healthy living behaviours and promoting health.

Theme 2. LCHF controversies and beliefs

Almost all the health professionals had heard of the LCHF diet, though their understanding varied significantly. For those unfamiliar with it, ‘A low-carbohydrate high-fat diet is a way of eating that restricts the amount of carbohydrates and increases the number of healthy fats that you eat to optimize your health and nutrition’. Some professionals recognised LCHF diets as the Banting or Tim Noakes diet, as they said that these terms have been popularised over the past few years in South Africa, particularly through social media. While some participants correctly described LCHF as a diet promoting whole, unprocessed foods, others had limited knowledge about the specific foods recommended in the diet. When discussing their opinions on LCHF diets, health professionals frequently used vague, generalized statements such as “long-term health” (Female physician) or “not sustainable” (Female dietitian), indicating uncertainty or a lack of critical engagement with the diet. A significant number of participants had personal experience or first-hand knowledge of LCHF diets, with many describing positive outcomes such as needing to eat less, having increased energy levels, and having reduced cravings. However, some stopped following LCHF due to challenges such as a lack of family support or the perception that the diet was expensive. Those who had initially been sceptical of LCHF often noted that it contradicted their formal university nutrition education, particularly regarding the role of fruit, fibre, and starch in the diet.

Public controversy surrounding LCHF further reinforced scepticism among some health professionals, leading them to express concerns and share their reservations with patients considering or already following the diet. Their primary concern focused on the high fat content of LCHF diets and whether the diet was financially sustainable for patients from low-income communities. Many professionals believed that LCHF foods are expensive, making it difficult for patients to maintain a long-term diet. Some were willing to support patients who chose to follow LCHF, but their support was often conditional on closely monitoring patients for potential negative effects. While international consensus guidelines

recognize carbohydrate restriction as an effective approach for managing type 2 diabetes, scepticism remains among some health professionals, particularly regarding its long-term sustainability and applicability in under-resourced settings. Some health professionals questioned whether LCHF could effectively treat metabolic disorders, largely due to ongoing controversy surrounding the diet and the lack of full scientific agreement on its long-term effects. Their main concerns included the sustainability of the diet, its affordability, the high intake of meat, the restriction of carbohydrates, and, most notably, its fat content owing to the perceived link between fat intake and cholesterol levels. Some professionals acknowledged that their views on cholesterol and dietary fat might be outdated, attributing this to limited access to updated nutrition science.

Health professionals also expressed concerns about patients' ability to fully understand and follow LCHF correctly, fearing that misinformation and a lack of nutritional literacy could lead to poor adherence or unintended health risks. Some worried that patients would struggle to transition to a low-carb diet, particularly without professional guidance and ongoing monitoring. When asked whether they would introduce LCHF to treat or prevent metabolic conditions, most stated that they would support patients who chose it but would not actively advocate for the diet due to the concerns outlined above. This cautious stance led many professionals to adopt a “wait-and-see” approach, where they would monitor patients rather than actively encourage the diet.

Some alternative reasons for their reluctance included the belief that LCHF is too restrictive and that vegetables should not be excluded from a healthy diet. Many professionals also believed that patients lacked adequate knowledge about their own health before adopting LCHF, further reducing their willingness to endorse the diet. The two dietitians interviewed highlighted adherence as a key challenge, stating that while LCHF may be effective for some individuals, an individualized dietary approach may be more appropriate. As a result, they did

not advocate for LCHF as a universal dietary solution, emphasizing instead that dietary interventions should be tailored to each patient's needs.

Theme 3: EBSA’s role in community nutrition

Some of the interviewees were familiar with the EBSA program, but the majority were introduced to it for the first time during the interview. Those who were aware of EBSA had patients who had participated in the program. For clarity, the EBSA program was defined to all participants as: ‘Eat Better South Africa is a nutrition education program for people from under-resourced communities that promotes low-carbohydrate high-fat diet or Banting diet to prevent or treat metabolic conditions. It consists of groups of people that meet every week for an educational session for a period of six weeks’.

Many professionals viewed group support as essential for dietary interventions, stating that changing lifestyle behaviours is easier when done collectively rather than individually. Some participants saw EBSA as a positive initiative for reshaping their dietary mindset and recognised its potential to alleviate pressure on healthcare facilities by reducing the incidence of diet-related chronic diseases. However, concerns about the EBSA program often overlapped with broader concerns about LCHF diets, particularly in terms of sustainability, affordability, and accessibility in low-income communities. Health professionals also emphasized the need for structured nutrition education programs tailored for healthcare workers themselves. Many expressed that they lacked the necessary tools and training to provide effective dietary guidance and stated that a program similar to EBSA, designed for health professionals, would help them better address nutrition-related diseases in their practice. While the majority of participants stated that they would support patients participating in EBSA, they specified the limits of their support, indicating that they would monitor but not necessarily promote the program’s dietary approach.

Despite these reservations, group support was considered the most valuable aspect of the EBSA program. Many professionals agreed that individual behaviour change is difficult and that patients are more likely to succeed when they receive support from their peers. Some participants valued the educational aspect of EBSA, stating that if more at-risk individuals joined similar community-based nutrition programs, the burden of chronic diseases on hospitals could be reduced. Concerns about EBSA primarily mirrored existing concerns about LCHF diets, with professionals questioning whether the diet could be adapted to the financial and food accessibility challenges of under-resourced communities. Some interviewees stressed the importance of tailoring dietary recommendations to local food prices and availability, ensuring that any dietary intervention remains affordable and practical for long-term adherence. Several health professionals also provided suggestions on how EBSA could better address food affordability and sustainability, indicating a willingness to engage with the program if these issues were explicitly considered in its implementation.

Theme 4: Understanding socioeconomic determinants of health

Health professionals broadly acknowledged the challenges that people living in under-resourced communities face, demonstrating awareness of the socioeconomic barriers to healthy living. Many participants referred to specific communities such as Ocean View and Masiphumelele, which are often highlighted in the media for high crime rates, violence, and safety concerns. Some health professionals reside in the same communities as their patients do, providing them with firsthand insight into the rationale behind food choices and the structural challenges that shape dietary behaviours. This theme captures interconnected issues related to food accessibility, affordability, safety, and knowledge. Several health professionals noted that for many individuals, food is not a priority, and that they are in a survival mode. The cost of food was often cited as an issue, emphasizing that healthier foods tend to be more expensive than processed and unhealthy options. Meat was consistently described as expensive, though

some also noted that vegetables and other fresh produce were increasingly costly, further complicating efforts to promote healthier dietary choices.

The interviewees cited poverty and lack of resources due to socio-economic circumstances as a challenge to healthy lifestyle choices and changes. According to the information that the participants provided, communities such as Ocean View and Masiphumelele face high levels of unemployment, crime, inadequate housing, and limited access to healthcare and nutrition education. Unemployment appeared to be a major contributing factor to the high level of poverty in these communities. Health professionals also considered limited education a barrier to adopting healthy lifestyle choices, changes, or nutritional interventions. They recognised that nutrition education could have a strong influence on changing unhealthy habits and improving long-term health outcomes. Alcohol abuse was mentioned as a contributing factor to both crime and the reinforcement of unhealthy behaviours, which further complicated adherence to medical and lifestyle recommendations. Compliance with prescribed medication, treatment, and lifestyle advice was identified as a challenge for the patients from the health professionals' perspective.

Health professionals acknowledged that culture and tradition strongly influence dietary habits and perceptions of health, which are often shaped by family and community norms. Cultural stigmatization around body weight and physical appearance was a recurring theme, particularly regarding women. Some participants noted that being slim was sometimes associated with poverty and malnutrition, whereas larger body sizes were perceived as indicators of wealth and well-being. Gender roles within households were also highlighted, as women were often responsible for preparing meals yet had to navigate traditional expectations that limited their decision-making power regarding food choices, health, and financial autonomy. Another key theme was the diverse cultural backgrounds within these communities, which influenced dietary habits and nutritional choices. Some health professionals discussed

how migration from rural to urban areas impacted dietary behaviours. In rural settings, individuals often had access to fresh, homegrown food and engaged in more physical activity. However, upon relocating to urban environments, they tended to maintain a high-carbohydrate diet while adopting more sedentary lifestyles, contributing to diet-related health concerns. The transition from rural to urban living was perceived as a challenge, as individuals often lost access to traditional food sources such as home gardening or farming, increasing their reliance on processed and nutrient-poor foods.

Health professionals noted that despite receiving nutritional advice, many patients do not adhere to dietary recommendations, often due to aversion to unfamiliar foods or deeply ingrained eating habits. They identified a preference for ultra-processed foods, explaining that these foods are not only widely available but also enjoyed for their taste, making compliance with healthier dietary guidance more challenging. Some professionals linked this preference to food addiction and eating disorders, highlighting the difficulty in breaking habitual consumption patterns of processed, high-sugar foods. An important cultural aspect raised by professionals was the link between food and social status in under-resourced communities. People with limited financial resources may prioritize purchasing branded, ultra-processed foods as a way to signal wealth or social belonging, even if those foods are nutritionally poor. Professionals observed that certain food choices were not solely based on affordability but also on perceptions of prestige and aspiration, further complicating efforts to promote healthier eating habits.

Discussion

This study is the first to explore health professionals' perceptions of a community-based program promoting LCHF diets in under-resourced South African communities. The findings reveal a general alignment with conventional dietary guidelines among health professionals, who emphasize the importance of vegetables and fruits while expressing concerns about dietary

fats and meat [49]. While physicians and nurses recognized the value of nutritional education in disease prevention, their ability to provide effective dietary counselling was hindered by systemic barriers, including time constraints, competing clinical responsibilities, and inconsistent nutritional messaging. [50]. This reflects a broader issue of confusion and inconsistency in nutritional advice, which can undermine the effectiveness of health promotion efforts.

The study also found that different healthcare professionals approach nutrition advice differently. Physicians admitted to having some nutritional knowledge but lacked confidence in offering dietary guidance, while nurses, who are often the primary healthcare providers, demonstrated more openness to new information and a greater willingness to provide lifestyle counselling, particularly in managing non-communicable diseases (NCDs) [51-55]. However, their ability to provide effective dietary counselling was limited by time constraints, competing responsibilities, and a lack of structured training. An in-depth qualitative investigation with nurses delved into their encounters while counselling diabetes patients on diet, exercise, and smoking cessation [55]. Nurses highlighted challenges like patients' limited knowledge and motivation, as well as their own counselling skills and time constraints, hindering effective lifestyle counselling. Despite these obstacles, the study revealed nurses' readiness to offer weight management advice as part of their professional duties, aligning with previous research findings [54, 55]. Dietitians, though fewer in number, adhered more strictly to existing dietary guidelines and were cautious about endorsing LCHF diets due to concerns about their sustainability and potential health risks [56]. While health professionals expressed difficulty in staying updated with advancements in nutrition science, they also relied heavily on widely accepted dietary principles without necessarily questioning or critically analysing them. Furthermore, many professionals agreed that more clinical trials were needed to reassess current dietary guidelines, reflecting an awareness of the evolving nature of nutrition science.

This duality, acknowledging the need for stronger evidence while continuing to use conventional dietary rhetoric, highlights the complexity of how professionals navigate nutritional knowledge in practice.

Despite the cautious approach of most health professionals, there was an acknowledgment of the potential benefits of LCHF diets in clinical nutrition, especially if patients are closely monitored. However, concerns about practical challenges in implementing diets were prevalent, including the cost and variety of recommended foods, the feasibility of adherence in low-income settings, and the alignment with national dietary guidelines were prevalent [34, 57, 58]. Health professionals generally viewed these barriers as more pressing than the debate over the diet's health effects, suggesting that practical implementation challenges could be the primary concern. While most health professionals claimed that they would support patients, their approach often inadvertently discouraged them, resembling "backhanded support", explicitly noted in some instances. Their reluctance stemmed from concerns about the diet's alignment with conventional dietary guidelines, a lack of confidence in providing LCHF-specific advice, and uncertainty about its long-term health impacts. Rather than actively endorsing LCHF diets, professionals supported patients out of a desire to respect patient autonomy, a stance known as therapeutic pragmatism, where healthcare providers adopt a supportive role despite personal doubts [59]. This meant that while they outwardly agreed to assist patients following an LCHF approach, their hesitance and underlying scepticism often resulted in a lack of meaningful guidance, leaving patients to navigate dietary changes largely on their own. Alternatively, physicians and nurses sometimes directed patients to dietitians, who, as revealed in interviews, might dissuade them from LCHF diets in favour of a more balanced macronutrient approach.

Some study participants had personal experiences with or first-hand knowledge of LCHF diets from friends or family, which tended to be positive. Interestingly, despite their

cautiousness in endorsing or advocating for the diet, some had personally experimented with it and experienced favourable results, reflecting findings from other studies in which individuals reported improved well-being, weight loss, and reduced hunger in the diet [60-63]. When discussing the LCHF diet and introducing the EBSA program as a nutrition and health education initiative promoting this approach, health professionals showed interest in the concept of group support, suggesting potential backing for community-based programs that provide such support. The availability of group support within the program was seen as a catalyst for encouraging dietary behaviour changes. However, this interest did not necessarily translate into full endorsement of the LCHF diet in communities, as concerns persisted about various barriers to implementation, including food costs, accessibility, and conflicting advice from different health workers. While LCHF diets are widely recognized in South Africa, their popularity is largely driven by media visibility and public discourse rather than strong institutional or professional endorsement. Many healthcare professionals remained cautious about formally supporting the diet, reflecting a broader global trend where media-driven interest does not always translate into clinical adoption [60]. This suggests that scepticism about LCHF is not solely a matter of questioning its health implications but rather an uncertainty about how it can be practically adopted in resource-limited settings. Some professionals also noted that hesitation toward LCHF did not come solely from healthcare providers but was also present within the support groups themselves, which were composed of community members, including patients. This reluctance within the groups stemmed from conflicting advice received from different health workers, scepticism about non-traditional diets, and broader uncertainty about the feasibility of LCHF within low-income settings [61-63]. While some professionals acknowledged that the program might be effective in changing perceptions about diet, concerns about the LCHF approach were also raised.

There was a general recognition of the challenging environmental conditions in under-resourced South African communities, with food insecurity emerging as a significant concern due to issues of accessibility and affordability. Therefore, socioeconomic determinants emerged as critical barriers to dietary change. Additionally, although not explicitly highlighted, discussions also touched on women's safety concerns. Cultural influences and traditional dietary habits were frequently mentioned, echoing previous research [64-66]. These factors were seen as obstacles to both individual and community well-being. Health professionals often framed these challenges as significant barriers to dietary change rather than outright rejecting LCHF as a viable nutritional intervention. This rationalization of limited intervention in nutritional guidance aligns with previous observations [50]. The findings of this study highlight how culture, tradition, and socioeconomic status influence dietary behaviours and perceptions of health, often complicating the effectiveness of nutritional education and dietary interventions. The association between larger body sizes and wealth, as noted by health professionals, aligns with previous research showing that body image norms in some South African communities equate size with prosperity and well-being, while thinness is often linked to illness or poverty [67]. These perceptions, combined with gendered household roles, place women in a position where they are responsible for meal preparation but have limited autonomy in making food choices, reinforcing structural barriers to dietary change [68]. Additionally, the migration from rural to urban areas has disrupted traditional food systems, increasing reliance on processed and commercially available foods, which mirrors broader trends in the African nutrition transition [69].

Health professionals also expressed frustration over patients' low compliance with dietary recommendations, with many preferring familiar ultra-processed foods that are perceived as enjoyable and, in some cases, status symbols. This echoes previous research highlighting the role of food choices as social markers in economically constrained

environments, where individuals may prioritize brand-associated foods as symbols of affluence despite financial hardship [70]. These findings echo broader research on nutrition transitions in Africa, where urbanization and economic constraints drive reliance on processed foods [69]. Understanding these complex social and cultural influences is critical for designing effective, context-specific nutrition education programs that resonate with communities while addressing underlying barriers to dietary change. This perspective connects with the notion of "backhanded support" when it comes to assisting patients interested in following an LCHF diet. Health professionals acknowledged that unhealthy foods are often consumed because they are enjoyable [51,56,71-73], linking this to concepts of food addiction and predictors of problematic eating behaviours. However, many professionals believed that economic factors and social influences played a more decisive role than individual dietary preferences in shaping food choices. Notably, healthcare providers, particularly dietitians, appeared hesitant to challenge these preferences among participants.

This study has a temporal limitation, as data collection took place between November 2019 and February 2020, meaning that the findings capture a specific moment in time. Dietary discourse in South Africa may have evolved since then, particularly due to pandemic-related shifts in food access, nutrition policies, and public health priorities. While this study provides valuable insights into health professionals' perspectives, future research is needed to explore how these views may have changed with emerging dietary trends and transition initiatives. Based on the study findings, EBSA could improve their program by offering targeted training for healthcare providers, particularly nurses and physicians, to boost their confidence in recommending LCHF diets. This training should address misconceptions about dietary fats and provide strategies for monitoring patients effectively. Additionally, EBSA could develop resources that consider the socioeconomic challenges and cultural influences in under-resourced communities, offering practical and affordable dietary advice. Enhancing the group

support component could also strengthen community involvement and help sustain dietary changes, leading to better health outcomes. This study is a pioneering effort to explore health professionals' views on a community-based program promoting LCHF diets in underprivileged South African communities.

Qualitative interviews revealed that health professionals largely align with conventional dietary guidelines, emphasizing vegetables and fruits while expressing concerns about fats and meat. The diverse perspectives of physicians, nurses, and dietitians offer a comprehensive view, but the uneven sample size, limited facility access, the affiliation of one dietitian with an NGO, and the exclusion of non-English speakers may have limited the diversity of perspectives captured and introduced biases. Future studies should include non-English-speaking health professionals to capture broader cultural perspectives. Additionally, while dietitians are typically the primary providers of dietary counselling, in under-resourced primary care settings, nurses and physicians often take on this role due to staff shortages [52]. This reflects the real-world task-shifting that is common in South African healthcare, where non-specialist providers must frequently step in to deliver nutritional advice. While dietitians play a central role in dietary counselling, this study included physicians and nurses to capture how nutrition messages are communicated in primary care, aligning with community health models for under-resourced settings. Future research focusing solely on dietitians could provide more specialized insights into LCHF counselling and implementation.

By highlighting the crucial role of nurses in health promotion, this study underscores the need for further research to explore how task-shifting impacts dietary counselling and to address the broader systemic challenges influencing healthcare practices. Despite these challenges, the study offers valuable insights into health professionals' attitudes towards nutritional advice and LCHF diets, contributing to discussions on community healthcare and NCD management. Notably, some doctors privately believed in LCHF’s potential benefits—

often informed more by media than by formal training—but were reluctant to refer patients to dietitians, anticipating that dietitians would discourage LCHF in favour of conventional low-fat advice [13].

Conclusions

This study provides valuable insights into healthcare professionals' perspectives on a nutrition education program recommending LCHF diets, highlighting key challenges in their understanding and implementation within under-resourced South African communities. The professionals expressed uncertainty around LCHF, which was often shaped by conflicting guidelines, limited exposure to recent evidence, and scepticism about its long-term effects. The findings also reveal significant training gaps in nutrition education, with many professionals, mostly doctors and nurses, lacking confidence in providing dietary guidance beyond conventional recommendations. Additionally, socioeconomic barriers such as food affordability, cultural norms, and structural inequalities further complicate dietary behaviour changes in these communities. Despite these challenges, according to participants, EBSA presents a promising model for integrating nutrition education into healthcare strategies, particularly by offering structured support and practical guidance tailored to local contexts. Future efforts should focus on bridging knowledge gaps, strengthening professional training, and ensuring that dietary interventions are both evidence-based and accessible to those who need them most.

Acknowledgments

We acknowledge the health professionals who volunteered to participate in this study, and Cyber Transcription for transcriptions. Thank you to the hospitals and non-profit organizations for allowing the interviews to be conducted in their facilities.

Data availability

The full transcripts and raw data from this study cannot be shared publicly due to ethical and legal restrictions. The research involved interviews with healthcare professionals discussing potentially sensitive information related to their workplace, patients, and professional practices. The approved protocol, which was reviewed by the Human Research Ethics Committee of the University of Cape Town (HREC REF 512/2019), did not include provisions for public data sharing. Participants consented to participate in the study under the condition that their full transcripts and identifying information would remain confidential and would not be made publicly available. However, researchers who meet the criteria for access to confidential data may request access to de-identified datasets by contacting the Human Research Ethics Committee of the University of Cape Town (Hub-PhysiologicalSciences@uct.ac.za) or the P.I. Georgina Pujol-Busquets (georgipbg@uoc.edu).

Conflicts of interest

The authors declare no conflicts of interest. G.P.B. is a recipient of the Tim and Marilyn Noakes postdoctoral fellowship. The funders had no role in the design of the study; in the collection, analysis, or interpretation of data; in the writing of the manuscript; or in the decision to publish the results.

References

1. The Constitution and public health policy. Chapter 2. Health & Democracy. [Internet]. [cited 2024 Mar 17]. Available from: https://section27.org.za/wp-content/uploads/2010/04/Chapter2.pdf
2. Maphumulo WT, Bhengu BR. Challenges of quality improvement in the healthcare of South Africa post-apartheid: A critical review. Curationis. 2019;42(1):1–9. doi:10.4102/curationis.v42i1.1901.

3. Stuckler D, Basu S, Mckee M. Health care capacity and allocations among South Africa's provinces: Infrastructure-inequality traps after the end of apartheid. Am J Public Health. 2011;101(1):165–72. doi:10.2105/AJPH.2009.184895.
4. Baker A. What South Africa can teach us as worldwide inequality grows. Time. 2019;1. [Internet]. [cited 2024 Mar 17]. Available from: https://time.com/longform/south-africa-unequal-country/
5. Oladipo JA. Utilization of health care services in rural and urban areas: A determinant factor in planning and managing health care delivery systems. Afr Health Sci. 2014;14(2):322-33. doi:10.4314/ahs.v14i2.6.
6. Turok I. Urbanisation and development in South Africa: Economic imperatives, spatial distortions and strategic responses. Urbanisation and Emerging Population Issues: Working Paper; 8. London: International Institute for Environment and Development (IIED). [Internet]. [cited 2024 Mar 17]. Available from: http://hdl.handle.net/20.500.11910/3082.
7. Kon ZR, Lackan N. Ethnic disparities in access to care in post-apartheid South Africa. Am J Public Health. 2008;98(12):2272–7. doi: 10.2105/ AJPH.2007.127829.
8. Voget U. Professional nurses' lived experiences of moral distress at a district hospital. [Master's Thesis]. Stellenbosch University, South Africa; 2017. Available from: https://core.ac.uk/download/pdf/188221241.pdf.
9. Coovadia H, Jewkes R, Barron P, Sanders D, McIntyre D. The health and health system of South Africa: Historical roots of current public health challenges. Lancet. 2009;374(9692):817–34. doi:10.1016/S0140-6736(09)60951-X.
10. Kruger HS, Puoane T, Senekal M, van der Merwe M-T. Obesity in South Africa: Challenges for government and health professionals. Public Health Nutr. 2005;8(5):491–500. doi: 10.1079/phn2005785.

11. Mayosi BM, Lawn JE, Niekerk A van, Bradshaw D, Karim SSA, Coovadia HM, et al. Health in South Africa: Changes and challenges since 2009. Lancet. 2012;380(9858):5–19. doi: 10.1016/S0140-6736(12)61814-5.
12. Sanders D, Chopra M. Key challenges to achieving health for all in an inequitable society: The case of South Africa. Am J Public Health. 2006;96(1):73–8. doi: 10.2105/AJPH.2005.062679.
13. Crowley J, Ball L, Hiddink GJ. Nutrition in medical education: A systematic review. Lancet Planet Health. 2019;3(9). doi: 10.1016/S2542-5196(19)30171-8.
14. Gallegos D. Global challenges and opportunities for dietitians. Nutr Diet. 2020;77:179-81. doi: 10.1111/1747-0080.12614.
15. Kohlmeier M, Nowson CA, DiMaria-Ghalili RA, Ray S. Nutrition education for the health care professions. J Biomed Educ. 2015;2015:1–2. doi: 10.1155/2015/380917.
16. Köse S, Yildirim G. The effect of a nutrition education. Int J Caring Sci. 2020;13(6):573–82. [Internet]. [cited 2024 Mar 17]. Available from: https://www.internationaljournalofcaringsciences.org/docs/63_selmin_%20original_13_1.pdf
17. Riddell MA, Edwards N, Thompson SR, Bernabe-Ortiz A, Praveen D, Johnson C, et al. Developing consensus measures for global programs: Lessons from the Global Alliance for Chronic Diseases Hypertension research program. Global Health. 2017;13(1):1–12. doi:10.1186/s12992-017-0242-8.
18. Popkin BM. Global nutrition dynamics: The world is shifting rapidly toward a diet linked with noncommunicable diseases. Am J Clin Nutr. 2006;84(2):289-98. doi: 10.1093/ajcn/84.1.289.
19. Beckfield J, Olafsdottir S, Bakhtiari E. Health inequalities in global context. Am Behav Sci. 2013;57(8):1014-1039. doi: 10.1177/0002764213487343.

20. Perez-Escamilla R, Bermudez O, Buccini GS, Kumanyika S, Lutter CK, Monsivais P, et al. Nutrition disparities and the global burden of malnutrition. BMJ. 2018;361:k2252. doi: 10.1136/bmj.k2252.

21. Kris-Etherton PM, Akabas SR, Douglas P, et al. Nutrition competencies in health professionals' education and training: A new paradigm. Adv Nutr. 2015;6(1):83–87. doi:10.3945/an.114.006734.

22. Viswanath K, Bond K. Social determinants and nutrition: Reflections on the role of communication. J Nutr Educ Behav. 2007;39(2). doi: 10.1016/j.jneb.2006.07.008.

23. Burgess A. Nutrition education in public health programs—What have we learned? Am J Public Health. 1961;51(11):1715–26. doi: 10.2105/ajph.51.11.1715.

24. Baute V, Sampath-Kumar R, Nelson S, Basil B. Nutrition education for the health-care provider improves patient outcomes. Glob Adv Health Med. 2018;7:216495611879599.

25. Schultz JA. Community-based nutrition education: Documentation and evaluation of effectiveness [Master's Thesis]. Iowa State University; 2015. Available from: https://dr.lib.iastate.edu/handle/20.500.12876/28900/

26. Oldewage-Theron WH, Egal A. Impact of nutrition education on nutrition knowledge of public school educators in South Africa: A pilot study. Health SA Gesondheid. 2012;17(1). [Internet]. [cited 2024 Mar 17]. Available from: http://www.scielo.org.za/scielo.php?script=sci_arttext&pid=S2071-97362012000100011&lng=en&nrm=iso

27. Rustad C, Smith C. Nutrition knowledge and associated behaviour changes in a holistic, short-term nutrition education intervention with low-income women. J Nutr Educ Behav. 2013;45(6):490–8. doi: 10.1016/j.jneb.2013.06.009.

28. Walsh CM, Dannhauser A, Joubert G. Impact of a nutrition education programme on nutrition knowledge and dietary practices of lower socioeconomic communities in the Free

State and Northern Cape. S Afr J Clin Nutr. 2003;16(3):89–95. [Internet]. [cited 2024 Mar 17]. Available from: https://www.semanticscholar.org/paper/Impact-of-a-nutrition-education-programme-on-and-of-Walsh-Dannhauser/33731bd4dc0e3c542fbd87cf837f380cc802eada

29. Whitehead D, Wang Y, Wang J, Zhang J, Sun Z, Xie C. Health promotion and health education practice: Nurses' perceptions. J Adv Nurs. 2008;61(2):181–7. doi: 10.1111/j.1365-2648.2007.04479.x.
30. Martin L, Leveritt MD, Desbrow B, Ball LE. The self-perceived knowledge, skills and attitudes of Australian practice nurses in providing nutrition care to patients with chronic disease. Fam Pract. 2014;31(2):201-8. doi: 10.1093/fampra/cmt070.
31. Volek JS, Feinman RD. Carbohydrate restriction improves the features of Metabolic Syndrome. Nutr Metab. 2005;2:1–17. doi: 10.1186/1743-7075-2-31.
32. Vorster HH, Badham JB, Venter CS. An introduction to the revised South African food-based dietary guidelines. S Afr J Clin Nutr. 2013;26(sup1):S5-S12.
33. Feinman RD, Vernon MC, Westman EC. Low carbohydrate diets in family practice: What can we learn from an internet-based support group. Nutr J. 2006;5(1). doi: 10.1186/1475-2891-5-26.
34. Sboros S. The Noakes Trial, two years later. 2020. [Internet]. [cited 2024 Mar 17]. Available from: https://www.nmsociety.org/the-scene/noakestrial.
35. Murdoch C, Unwin D, Cavan D, Cucuzzella M, Patel M. Adapting diabetes medication for low carbohydrate management of type 2 diabetes: A practical guide. Br J Gen Pract. 2019;69(684):360-1. doi: 10.3399/bjgp19X704525.
36. Mozaffarian D. Dietary and policy priorities for cardiovascular disease, diabetes, and obesity. Circulation. 2016;133(2):187–225. doi: 10.1161/CIRCULATIONAHA.115.018585.

37. Banting WB. Letter on corpulence, addressed to the public: Third Edition. Obes. Res. 1987. [Internet]. [cited 2024 Mar 17]. Available from: https://onlinelibrary.wiley.com/doi/pdf/10.1002/j.1550-8528.1993.tb00605.x
38. Pujol-Busquets G, Smith J, Larmuth K, et al. Exploring the perceptions of women from under-resourced South African communities about participating in a low-carbohydrate high-fat nutrition and health education program: A qualitative focus group study. Nutrients. 2020;12(4):894. doi: 10.3390/nu12040894.
39. Pujol-Busquets G, Smith J, Fàbregues S, et al. Community assessment for a low-carbohydrate nutrition education program in South Africa. Nutrients. 2023;15(1):67. doi: 10.3390/nu15010067.
40. Pujol-Busquets G, Smith J, Fàbregues S, Bach-Faig A, Larmuth K. Mixed methods evaluation of a low-carbohydrate high-fat nutrition education program for women from underserved communities in South Africa. Appetite. 2025;204:107725. doi: 10.1016/j.appet.2024.107725.
41. Johnston BC, Kanters S, Bandayrel K, et al. Comparison of weight loss among named diet programs in overweight and obese adults: A meta-analysis. JAMA. 2014;312(9):923-33. doi: 10.1001/jama.2014.10397.
42. Franz MJ, VanWormer JJ, Crain AL, et al. Weight-loss outcomes: A systematic review and meta-analysis of weight-loss clinical trials with a minimum 1-year follow-up. J Am Diet Assoc. 2007;107(10):1755-67. doi: 10.1016/j.jada.2007.07.017.
43. Brink JG, Hassoulas J. The first human heart transplant and further advances in cardiac transplantation at Groote Schuur Hospital and the University of Cape Town. Cardiovasc J Afr. 2009;20(1):31-5. PMID: 19287813; PMCID: PMC4200566.

44. Adler P, Adler P. How many qualitative interviews is enough? Natl Cent Res Methods Rev Pap. 2012;1–42. [Internet]. [cited 2024 Mar 17]. Available from: http://eprints.ncrm.ac.uk/2273/.
45. Braun V, Clarke V. Thematic analysis: A practical guide. London: SAGE Publications; 2022.
46. Braun V, Clarke V. Using thematic analysis in psychology. Qual Res Psychol. 2006;3(2):77–101. doi: 10.1191/1478088706qp063oa.
47. Bloor M. Techniques of validation in qualitative research: a critical commentary. In: Miller G, Dingwall R, editors. Context and method in qualitative research. London: SAGE Publications; 1997. p. 37–50.
48. Tong A, Sainsbury P, Craig J. Consolidated criteria for reporting qualitative research (COREQ): a 32-item checklist for interviews and focus groups. Int J Qual Health Care. 2007;19(6):349-357. doi:10.1093/intqhc/mzm042.
49. Steyn N, Ochse R. Food-Based Dietary Guidelines for South Africa: "Enjoy a variety of foods": As a food-based dietary guideline. S Afr J Nutr. 2013;26(3):13–7. (Supplement):S1-S164. [Internet]. [cited 2024 Mar 17]. Available from: https://openknowledge.fao.org/server/api/core/bitstreams/2cf12134-bb2d-47b1-92b0-403b028c60ad/content
50. Hunter-Adams J, Battersby J. Health care providers' perspectives of diet-related non-communicable disease in South Africa. BMC Public Health. 2020;20(1). doi: 10.1186/s12889-020-8364-y.
51. McKinlay E, Plumridge L, McBain L, McLeod D, Pullon S, Brown S. "What sort of health promotion are you talking about?": A discourse analysis of the talk of general practitioners. Soc Sci Med. 2005;60(5):1099–106. doi: 10.1016/j.socscimed.2004.06.041.

52. Smolowitz J, Speakman E, Wojnar D, Whelan EM, Ulrich S, Hayes C, et al. Role of the registered nurse in primary health care: Meeting health care needs in the 21st century. Nurs Outlook. 2015;63(2):130–6. doi: 10.1016/j.outlook.2014.08.004.
53. Barton AJ, Skiba DJ, Sorensen L. Preparing the next generation of advanced practice nurses. Stud Health Technol Inform. 2004;107(Pt 2):860-4. PMID: 15360934.
54. Xu X, Parker D, Ferguson C, Hickman L. Where is the nurse in nutritional care? Contemp Nurse. 2017;53(3):267-70. doi: 10.1080/10376178.2017.1370782.
55. Jansink R, Braspenning J, van der Weijden T, Elwyn G, Grol R. Primary care nurses struggle with lifestyle counseling in diabetes care: A qualitative analysis. BMC Fam Pract. 2010;11. doi: 10.1186/1471-2296-11-41.
56. Marshall AP, Takefala T, Williams LT, Spencer A, Grealish L, Roberts S. Health professional practices and their influence on nutritional intake of hospitalised patients. Int J Nurs Sci. 2019;6(2):162–8. doi: 10.1016/j.ijnss.2019.03.008.
57. Holmberg C. Politicization of the Low-Carb High-Fat diet in Sweden, promoted on social media by non-conventional experts. Int J E-Polit. 2015;6(3):27–42. doi: 10.4018/ijep.2015070103.
58. Guidelines Advisory Committee D. Scientific Report of the Dietary Guidelines Advisory Committee Advisory Report to the Secretary of Agriculture and Secretary of Health and Human Services. 2020. [Internet]. [cited 2024 Mar 17]. Available from: https://www.dietaryguidelines.gov/2020-advisory-committee-report.
59. Pilnick A, Hindmarsh J, Gill VT, editors. Communication in healthcare settings: policy, participation and new technologies. Chichester: Wiley-Blackwell; 2010.
60. Webster CC, Murphy TE, Larmuth KM, Noakes TD, Smith JA. Diet, diabetes status, and personal experiences of individuals with type 2 diabetes who self-selected and followed a

Low Carbohydrate High Fat diet. Diabetes Metab Syndr Obes. 2019;12:2567–82. doi:10.2147/DMSO.S227090.

61. Jallinoja P, Niva M, Helakorpi S, Kahma N. Food choices, perceptions of healthiness, and eating motives of self-identified followers of a low-carbohydrate diet. Food Nutr Res. 2014;58:1–9. doi: 10.3402/fnr.v58.23552.
62. Clarke C, Best T. Low-carbohydrate, high-fat dieters: Characteristic food choice motivations, health perceptions and behaviours. Food Qual Prefer. 2017;62:162–71. doi: 10.1016/j.appet.2019.104324.
63. Arthur TH. Perceptions and acceptability of Low Carbohydrate, High Fat (LCHF) diets among Māori whānau in Dunedin. [Master's Thesis]. University of Otago; 2018. [Internet]. [cited 2024 Mar 17]. Available from: https://ourarchive.otago.ac.nz/esploro/
64. Aubel J. The role and influence of grandmothers on child nutrition: Culturally designated advisors and caregivers. Matern Child Nutr. 2012;8(1):19–35. doi: 10.1111/j.1740-8709.2011.00333.x.
65. Cassel J. Social and cultural implications of food and food habits. Am J Public Health. 1957;47:732–40. doi: 10.2105/ajph.47.6.732.
66. Hartanto A, Lau IYM, Yong JC. Culture moderates the link between perceived obligation and biological health risk: Evidence of culturally distinct pathways for positive health outcomes. Soc Sci Med. 2020;244. doi: 10.1016/j.socscimed.2019.112644.
67. Puoane T, Fourie JM, Shapiro M, Rosling L, Tshaka NC, Oelefse A. "Big is beautiful" - an exploration with urban black community health workers in a South African township. S Afr J Clin Nutr. 2005;18(1):6-15. doi: 10.1080/16070658.2005.11734033.
68. Ndinda C, Uzodike UO, Chimbwete C, Pool R. Gender relations in the context of HIV/AIDS in rural South Africa. AIDS Care. 2007;19(7):844-9. doi: 10.1080/09540120701203923.

69. Vorster HH, Kruger A, Margetts BM. The nutrition transition in Africa: Can it be steered into a more positive direction? Nutrients. 2011;3(4):429-41. doi: 10.3390/nu3040429.

70. Seekings J, Nattrass N. Class, race, and inequality in South Africa. Foreign Affairs. 2006;85(3):173. doi: 10.2307/20032025.

71. Rankin D, Backett-Milburn K, Platt S. Practitioner perspectives on tackling health inequalities: Findings from an evaluation of healthy living centres in Scotland. Soc Sci Med. 2009;68(5):925–32. doi: 10.1016/j.socscimed.2008.12.013.

72. Strang AB, Ager A. Psychosocial interventions: Some key issues facing practitioners. Intervention. 2003;1(3):2–12. [Internet]. [cited 2024 Mar 17]. Available from: https://citeseerx.ist.psu.edu/document?repid=rep1&type=pdf&doi=bd2177acc71bac53c13b6735ae7c918f85283aa8

73. Kok MO, Vaandrager L, Bal R, Schuit J. Practitioner opinions on health promotion interventions that work: Opening the “black box” of a linear evidence-based approach. Soc Sci Med. 2012;74(5):715–23. doi: 10.1016/j.socscimed.2011.11.021.

Click here to access/download
Supporting Information
IRB_Approval.pdf

Click here to access/download
Supporting Information
CRediT author statement.docx

Click here to access/download
Supporting Information
Supporting Information 1.docx

Click here to access/download
Supporting Information
Inclusivity-in-global-research-questionnaire.docx

Data Availability Statement

Click here to access/download
Supporting Information
Statement on Data Availability.docx

Between support and scepticism: Health professionals' perceptions of a nutrition education program promoting low-carbohydrate, high-fat diets in under-resourced South African communities

Georgina Pujol-Busquets[1,2,*], Kate Larmuth[1,3,4] , Christopher C Webster[1,3,4], James Smith[1,3,4], Ahtisham Younas[5,*], Sergi Fàbregues[2,6] and Anna Bach-Faig[2]

[1]Division of Physiological Sciences, Department of Human Biology, University of Cape Town, South Africa.

[2]NUTRALiSS Research Group, Faculty of Health Sciences, Universitat Oberta de Catalunya (UOC), Barcelona, Spain.

[3]International Federation of Sports Medicine (FIMS) Collaborative Centre of Sports Medicine, HPALS, University of Cape Town, South Africa.

[4]Research Center for Health through Physical Activity, Lifestyle and Sport, Division of Physiology, Department of Human Biology, Faculty of Health Sciences, University of Cape Town, Cape Town, South Africa.

[5]Faculty of Nursing, Memorial University of Newfoundland, St. John's, NL, Canada.

[6]Department of Psychology and Education, Universitat Oberta de Catalunya (UOC), Barcelona, Spain.

*Corresponding author: ay6133@mun.ca

Abstract

The burden of non-communicable diseases (NCDs) continues to rise, emphasizing the need for effective dietary interventions. Programs ~~like~~ such as *Eat Better South Africa (EBSA)* advocate for low-carbohydrate high-fat (LCHF) dietary choices, especially in disadvantaged communities. However, the adoption of such approaches among healthcare professionals remains contentious. This qualitative study explores healthcare professionals' perspectives on nutrition and the EBSA program, drawing on 16 in-depth individual interviews with physicians, nurses, and dietitians from False Bay Hospital, Groote Schuur Hospital, and a primary health clinic in Hout Bay, Western Cape, South Africa. Thematic analysis of the interviews revealed four main findings. Healthcare professionals lacked confidence in their nutritional knowledge, and while many were familiar with the LCHF diet, opinions varied regarding its sustainability and health implications. Concerns were raised about the high fat content and affordability of LCHF foods. Professionals acknowledged the value of group support in ~~behavior~~behaviour change, as promoted by EBSA, but expressed reservations about its strong emphasis on LCHF diets. Key challenges identified for patients included poverty, cultural beliefs, limited education, and access to nutritious foods. The findings highlight a reliance on traditional dietary advice, with uncertainties about the feasibility and affordability of LCHF diets~~, potentially conflicting with programs like~~ ~~such as~~ ~~EBSA~~. Th~~is study contributes~~ese findings offer novel insights into the complexities of implementing community-based dietary interventions in South Africa, with implications for policy and practice. ~~healthcare professionals' perspectives on LCHF diets and community-based interventions in South Africa, reflecting both cautious endorsement and critical concerns about their implications.~~

Formatted: Font: Italic

Keywords

perspectives on nutrition; healthcare professionals; low-carbohydrate, high-fat diet (LCHF); under-resourced communities; South Africa; thematic analysis; qualitative research. thematic analysis; low carbohydrate high fat; South Africa; under-resourced communities; healthcare professionals.

Introduction

South Africa's public health system faces major challenges, including long wait times, resource shortages, poor management, inadequate infection control, and workforce deficits—exacerbated by inequitable resource distribution, rapid urbanization, and the dual burden of infectious and non-communicable diseases (NCDs) increasing [1-9]. Within this strained system, In South Africa, all citizens, including undocumented immigrants, are constitutionally entitled to healthcare services, available through both public facilities and private sectors, often requiring payment. The District Health System, a central part of the public sector, is the primary access point for many [1]. However, public health institutions face significant challenges, including long wait times, poor hygiene, inadequate infection control, preventable errors leading to litigation, medicine and equipment shortages, and poor recordkeeping [2-4]. These issues stem from unequal resource distribution, rapid urbanization, rising disease rates, and slow healthcare system restructuring [5-7]. Additionally, a shortage of healthcare professionals, exacerbated by inadequate training, recruitment, retention, and mismanagement, further strains the system [8,9].

tThe healthcare workforce is crucial to advancing universal healthcare goals, particularly in addressing South Africa's complex disease challenges, both infectious and non-communicable [2,9-12]. Despite its critical role in health, nutrition remains poorly integrated into healthcare training, leading to inconsistent advice and under prepared professionals. Yet globally, nutrition is increasingly recognised as central to disease prevention and management, with dietary interventions often matching or exceeding pharmacotherapy in effectiveness and

Formatted: Font: (Default) Times New Roman, Not Bold

Formatted: Font: (Default) Times New Roman

Formatted: Font: (Default) Times New Roman, Not Bold

Formatted: Font: (Default) Times New Roman

Formatted: Font: (Default) Times New Roman, Not Bold

Formatted: Font: (Default) Times New Roman

Formatted: Font: (Default) Times New Roman, Not Bold

cost-efficiency Despite nutrition's the significant impact of nutrition on health, healthcare training programs often lack comprehensive integration of nutritional science, leading to conflicting advice and insufficient training to address nutrition-related conditions effectively [13-16]. This is a global concern, as nutrition profoundly influences health outcomes [17-20]. International efforts are bridging gaps in nutrition education, supported by evidence of the effectiveness of dietary and physical activity interventions, which often rival pharmacological treatments in efficacy, safety, and cost-effectiveness [13,16, 21- 24]. Studies consistently show that nutrition interventions reduce the incidence and severity of diet-related non-communicable disease (NCDs), improve patient outcomes, reduce healthcare costs, and enhance the well-being of healthcare professionals, especially in acute care settings [25–30].

Various nutritional education initiatives are active in low socio-economic areas of South Africa to address the burden of NCDs, with *Eat Better South Africa (EBSA)* being one prominent program [31]. EBSA promotes a low-carbohydrate high-fat (LCHF), dietary approach, focusingwith an emphasis, on nutrient-dense, minimally processed foods [31]. InThe LCHF approach contrasts with, the South African Food-Based Dietary Guidelines, , which emphasisze balanced diets, with staple whole grains, moderate fat intake, and a heavy focus on plant-based foods [32]. EBSA aims to empower individuals, particularly women, through dietary education, believing in the transformative power of education on health behaviours and overall well-being [31]. EBSA advocates for low-carbohydrate high-fat (LCHF) diets are supported , which someby some clinicians and professional societies support as an effective treatments for diabetes and obesity management [31 33]. However, this approach is contentious, with conflicting findings polarisizing expert opinion and potentially causing confusion among healthcare professionals and patients [34]. Despite these controversies, carbohydrate restriction for managing type 2 diabetes is supported by international consensus guidelines [35,36]. International consensus guidelines cautiously support carbohydrate

Formatted: Font: Italic

Formatted: English (United Kingdom)

Formatted: Font: Not Bold, English (United Kingdom)

Formatted: English (United Kingdom)

Formatted: English (United Kingdom)

Formatted: Font: Not Bold

restriction for type 2 diabetes [35,36], yet historical precedents—such as William Banting's 19th-century dietary polemics—highlight the enduring controversy surrounding this approach [37].Although this concept is not new, dating back to as early as 1869 with William Banting, even then Banting acknowledged and anticipated controversy and reputational risks associated with this diet [37].

In focus group discussions involving previous participants of EBSA programs, participants noted differences between the dietary guidance provided by healthcare professionals, which aligns with a Western Standard diet, in their communities, and the EBSA recommendations [38-40]. Some healthcare professionals who were initially skepticalsceptical of the EBSA LCHF diet have shifted towards supporting it, as they witnessed positive health outcomes in their patients. EBSA advocates for a lower-carbohydrate, higher-healthy-fat diet, aligning with many current dietary recommendations. TheA mixed -methods evaluation of the EBSA program demonstratedshowed significant improvements in metabolic health markers among participants after six months, including weight loss, reduced waist circumference, and improved glycemicglycaemic control, thoughalthough adherence challenges persistedremained a challenge due to socioeconomic factors [40]. Additionally, an earlier study reported that program participants felt empowered through nutritional education, but required ongoing support to sustain dietary changes [38]. A qualitative community assessment exploring women's willingness to participate in an EBSA program highlighted both interest in and concerns about the affordability of LCHF diets, as well asnd challenges related to dietary transitions [39]. These findings indicate that while EBSA has had measurable success in improving metabolic health, practical barriers to long-term adherence remain, necessitating better integration with healthcare professionals and community resources.

Formatted: Indent: First line: 0.5"

While some health professionals may support LCHF diets for certain individuals or specific health conditions, others may have reservations based on concerns about long-term health effects, individual variability, sustainability, adherence, and potential nutrient deficiencies [41,42]. Although the EBSA program has shown positive outcomes in improving dietary behaviours, and metabolic health among participants, healthcare professionals hold varying opinions on its dietary approach, highlighting the need to explore their perspectives in greater depth~~Understanding healthcare professionals' perceptions of the EBSA program, LCHF diets and community nutrition practices is crucial for program improvement~~. This article aims to explore healthcare professionals' perspectives on the EBSA program, particularly focusing on the effectiveness and sustainability of the LCHF diet in managing NCDs within South Africa. While the primary objective is to examine how healthcare professionals perceive the effectiveness of the EBSA program and its dietary recommendations, this~~e~~ study also investigates the barriers and facilitators that influence their willingness to support or integrate EBSA dietary guidance into their practice. Furthermore, it assesses how professional training, healthcare system constraints, and exposure to evolving nutrition science shape ~~their~~the attitudes of these healthcare professionals toward LCHF diets and nutrition education. It examines the alignment and potential conflicts between the nutritional advice given by professionals and the guidelines promoted by EBSA, identifying gaps in nutrition education and training. This~~e~~ article also explores the broader public health implications of integrating such dietary recommendations~~,~~ and seeks to provide insights for refining the EBSA program to better meet the health needs of South Africa's diverse population.

Formatted: Font: Not Bold

Formatted: Font: Not Bold

Materials and methods

This is a qualitative study using in-depth individual interviews (IDIs) with health professionals from health care facilities that would service ~~the ~~current and future EBSA

programs. The research team included experts in qualitative research and nutrition science. ~~the of qualitative research,whichsan of the phenomena under investigation~~

Setting

Formatted: Font: (Default) Times New Roman, 16 pt, Not Italic, Font color: Black

Formatted: Heading 5,2.1.1., Left, Indent: First line: 0"

Formatted: Font: 16 pt, Font color: Black

~~They~~ Participants were selected from two tertiary institutions, namely False Bay Hospital (Fish Hoek, Cape Town) and Groote Schuur Hospital (Observatory, Cape Town), and a primary health clinic in Hout Bay (Hout Bay, Cape Town). False Bay Hospital is a public hospital that was opened in 1965 in Fish Hoek, a town approximately 30 kilometres south of Cape Town. The hospital has approximately 80 beds, and its facilities include a 24-Hour Emergency Unit. It is the only district hospital in the South Peninsula Health District of the Metro Region and serves many communities, including Simonstown, Masiphumelele, Ocean View, Clovelly, and many ~~more~~ other surrounding areas. Groote Schuur Hospital is one of Cape Town's largest government-funded academic hospitals and was officially opened in 1938. The hospital has almost 900 beds and it is renowned as the hospital where the world's first human-to-human heart transplant was performed in 1967 by Christiaan Barnard and his team [~~43,~~43~~4~~]. The Hout Bay health clinic non-profit organization works mostly in ~~the~~ an informal settlement called Imizamo Yethu and provides nutrition and health education to the community. Hout Bay is a seaside town in the Western Cape P~~p~~rovince situated in the Cape Peninsula, 20 kilometres south of Cape Town.

Participants

Formatted: Font: Not Italic

To be eligible for the study, participants had to be health professionals; work in and see patients from the communities where EBSA has conducted a nutrition education program or a community where EBSA is planning to run future programs; and be able to understand and speak English. Community health clinics were contacted by email or phone ~~calls~~ to identify potential participants. A researcher explained the study to interested participants, either via phone or at a scheduled meeting, and~~,~~ if they met the inclusion criteria, they were provided

with a study information sheet and the informed consent form. An interview visit was then scheduled at a convenient time and place for the participants. The p~~P~~articipants met individually with a researcher for ~~about~~ approximately thirty minutes to an hour in a private venue within the participating health facilities, ensuring both privacy and accessibility, where they signed the informed consent form and were interviewed in English. Although participants were selected from some facilities serving communities where EBSA had run programs, this did not guarantee their direct involvement in or awareness of the program itself. Additionally, including professionals from communities where EBSA was~~is~~ planning future programs allowed the study to capture prospective opinions on the program, offering insight into potential facilitators and barriers before implementation.

Procedures

The IDIs were conducted between 29th of November and 21st February 2020 and lasted, on average, 30 minutes. The number of participants ~~aligns~~is consistent with ~~qualitative research~~ recommendations in the qualitative research literature, which suggests that meaningful insights can be ~~drawn~~gained from smaller, information-rich samples ~~The sample size is consistent with recommendations in the literature which ranges between 5 to 50 participants~~ [44~~5,46~~]. According to th~~is~~e literature, the first five to six IDIs generally produce the majority of ~~the~~ new data, and the majority of concepts are identified within the first 10 interviews. As such, new participants were interviewed until saturation was reached [44~~5,46~~]. However, as outlined by Braun and Clarke, we acknowledge that ~~as outlined by Braun and Clarke,~~ the concept of data saturation is debated, with an emphasis on depth of interpretation rather than a fixed threshold for thematic exhaustion [45]. Participants were interviewed on a one-on-one basis to explore their understanding and perceptions of chronic disease, nutrition, ~~low-carbohydrate high-fat~~ LCHF diets, and the EBSA program. A semi structured interview guide (see ~~Table 1~~~~Supplementary appendix~~SI1~~Supporting Information~~ File~~1~~) was used which

Formatted: Font: Not Italic

Formatted: Font: Not Bold

Formatted: Font: Not Bold

Formatted: Font: Not Bold

Formatted: Font: Not Bold

Formatted: Font: Not Bold

included both open-ended and probing questions as a guide for the discussion, although questions were not necessarily asked verbatim. Probing questions were asked only if the subjects had not already been covered.

The interview guide was developed based on a review of the literature on healthcare professionals' perceptions of dietary interventions, particularly low-carbohydrate diets. It was piloted with two health professionals (not included in the final sample) to ensure the relevance, clarity, and cultural appropriateness of the questions. The final guide followed a semi-structured format, allowing for follow-up questions and probing to explore emerging topics in depth. It covered ~~several~~the following key areas: healthcare providers' perceptions of their role in community health; major health concerns identified in their communities; views on NCDs such as obesity, diabetes, and cardiovascular disease; the type of dietary advice offered at their healthcare facilities; perspectives on the role of nutrition in chronic disease prevention and treatment; their understanding of local dietary patterns; and their awareness and impressions of the EBSA programme and the LCHF diet, including views on the feasibility of implementing such interventions in their communities.

~~The pinterview the ation of the on~~~~The q~~All interviews were audio-recorded, with the permission of the participants. The i~~I~~nterviews were conducted by GPB and saved in a password-protected folder. The r~~R~~ecordings were transcribed by a professional transcription service (Cyber Transcription, ~~(~~South Africa) ~~in the original language. T~~and the accuracy and editing of the transcriptions were double-checked by an external qualitative researcher for rigour and appropriateness. Each transcribed interview was compared with the corresponding audio~~,~~ to ensure that the transcription had been done in context and captured the interview as accurately as possible. ~~u~~

~~Table 1. Interview questions posed to participant.~~

Formatted: English (United Kingdom)

Formatted: English (United Kingdom)

**In-depht questions for healthcare professionals**
How would you describe your role as a health care provider in [community]?
What do you think are the major health problems/ diseases in [community]?
What are your thoughts about NCDs like obesity, diabetes, cardiovascular disease in [community]?
What lifestyle advice does the health care centre you work at offer to patients with chronic diseases, like obesity, diabetes and cardiovascular disease?
What role do you think nutrition plays in chronic diseases in [community]?<br>• Describe the foods/ nutrients/ diets you think lead to chronic diseases.<br>• Describe the foods/ nutrients/ diets that you think should be used to prevent/ treat chronic disease in [community].
How would you describe the diets of the patients that you see from [community]?<br>• Why do you think people from [community] eat this way?
Have you heard of the Eat Better South Africa nutrition education program?<br>• How and what did you hear about it?<br>• If you had patients taking part in the EBSA program, how was their experience?
Could you describe the diet that you think EBSA recommends?
What do you think about EBSA holding a program in [community]?
How did you / would you respond if one of your patients told you they were following an EBSA/ Banting/ low carbohydrate high fat diet?
Have you heard about “Banting” or low-carbohydrate high-fat diets?<br>• Could you describe what a Banting or LCHF diet would look like?

- What are your general impressions of this diet?
- How do you think this diet relates to chronic diseases? Do you think it might make it better or worse?

How do you think nutrition can be improved for people living in [community]?

Data analysis

Braun and Clarke’s approach to thematic analysis [47] was used to identify and analyse themes with NVivo 12 software (QSR Internationality Ltd., Melbourne, Australia). All the researchers participated in the analysis, which was conducted in a stepwise fashion. Braun and Clarke’s approach to thematic analysis [46] guidedwas followed the identification and development of themes using NVivo 12 software (QSR International, Melbourne, Australia). The 2006 version of Braun and Clarke’s thematic analysis was used. This version is characterised by its epistemological flexibility and openness to the use of codebooks, a feature that has since been abandoned by the authors in more recent iterations of this analytical method (Braun & Clarke, 2022[45]).

Two researchers (CCW and GPB) independently developed initial codebooks based on inductive readings of the data, which were then discussed and merged into a single codebook applied across the dataset. Coding was iterative and collaborative, with regular discussions to refine themes and ensure consistency. While informed by Braun and Clarke’s 2006 framework, the analysis reflects a codebook approach.

The analysis began withFirst, CCW and GPB reading the transcripts several times, and summarisszinged them, and making notes on ideas and potential codese transcripts. Second, These two researchers then each inductively developed a codebook two respective codebooks were developed inductivelybased on the summaries and notes generated by reading the transcripts in the previous step. Then, they by CCW and GPB, based on the previous readings.

Third, CCW and GPB discussed their individual codebooks, and merged them into one codebook, and . Finally, the two researchers independently coded three randomly selected interviews using separate NVivo projects. Thereafter, CCW and GPB then met again to revise compare their respective coding, and disagreements were discussed and resolved disagreements by consensus. The content and structure of the final codebook was finalised through consensus discussions involving ABF, SF, and KL, to ensure consistency and clarity in the coding framework.

Initially, a selection of transcripts was double-coded independently by GPB and CCW to establish a shared understanding of how the codes applied across the data. Once agreement on the codebook was reached among the members of the research team, the remaining interviews were divided into two groups of eight, with each group assigned to one analyst (GPB or CCW). Both sets were coded within the same NVivo project to facilitate coherence. GPB and CCW held regular meetings to discuss their interpretations, resolve discrepancies, and critically engage with the data. These collaborative discussions enabledallowed for the refinement of the coding framework and the, development of sub-themes and themes, and ensuringred consistency in analytical decisions. Emerging The ththemes developed were then visually organised by GPB using thematic maps, which supported further analysis and interpretation. During and after the interviews, the research team systematically recorded observations and took field notes, including contextual insights and paraverbal cues such as tone, pauses, and emphasis. Although this data was not formally coded, it was used in the process of interpreting and understanding the participants' narratives and in the development of the themes.The content and structure of the final codebook was subsequently finalised, with consensus from ABF, SF, and KL to check for consistency in the codes. Thereafter, the interviews were divided into two groups, each containing eight interviews, and one group each were was assigned to

Formatted: Indent: First line: 0"

~~GBP and CCW. Both coding schemes emerged in the same NVivo project, and GPB and CCW worked on developing the themes that were, subsequently, displayed by GPB as thematic maps.~~

The following step involved naming the themes and defining the main concepts of each theme, resulting in a total of four themes. ~~The last phase, which was mainly conducted by GPB, consisted of producing a report.~~ These ~~resulting~~ themes were shared with ABF, SF, KL and JS to confirm their relevance to the research questions. The fact that, ~~Although GPB conducted the interviews and participated in the analysis~~as explained above, two researchers ~~multiple coders~~ (CCW and GPB) were involved in the analysis of the data, and that the different steps of the analysis were reviewed by the other members of the research team, ~~used to~~ helped to ~~allow greater probability to~~ ensu~~r~~re that all the themes were identified and that the data ~~were~~ was ~~being consistently interpreted~~interpreted in a consistent way, avoiding the possibility of a premature closure of the analysis, ~~to~~ limiting the potential effect of bias in the ~~or~~ adoption of a singular point of view, and promoting the overall quality of the analysis. We did not use member checking for practical and methodological considerations. Logistically, time and resource constraints prevented us from following up with participants. Methodologically, we adopted an approach that recognises the dynamic and contextual nature of participants' perceptions, which may shift over time and are not necessarily fixed or unique. Participant feedback is shaped by the circumstances in which it is elicited and does not constitute a definitive test of validity [47].

Finally, the~~The~~ last step~~phase, which was mainly conducted~~carried out mainly by GPB, consisted of producing a written report of the study ~~t~~findings. ~~Member checking was not conducted in this study due to both practical and methodological considerations. Logistically, time and resource constraints prevented follow up with participants. Methodologically, we adopted a reflexive approach that recognises the dynamic and contextual nature of participants'~~

Formatted: English (United Kingdom)

~~perceptions, which may shift over time and are not necessarily fixed or singular. Participant feedback is shaped by the circumstances in which it is elicited and does not constitute a definitive test of validity [47]. Although field notes were not formally included in the coding framework, the research team systematically recorded observations during and after the interviews. These notes included contextual insights and paraverbal cues such as tone, pauses, and emphasis. While not coded as data, these elements were drawn upon during the interpretive process to help contextualize participants' narratives and to support a more nuanced, reflexive understanding of the data.~~

Formatted: Font: Font color: Custom Color(RGB(14,16,26)), English (South Africa)

~~Quotations~~ Quotes from the qualitative data were selected to illustrate the ~~emergent~~ themes identified in the data, ~~and~~ ensuring that each ~~these~~ captured the essence of the theme. ~~Square brackets indicate text added by the authors to provide clarity or context and an ellipsis indicates the omission of irrelevant parts of the quote. The health professionals' professions are included with the participant quotes with the gender provided in parentheses at the end of the quote.~~

The study adhered to the Consolidated Criteria for Reporting Qualitative Research (COREQ) guidelines to ensure that the methods and findings were reported in a transparent and rigorous manner [48].

Ethical considerations

The Human Research Ethics Committee (HREC REF 512/2019) of the University of Cape Town approved the study protocol and informed consent procedures. All procedures were performed ~~were~~ in accordance with the 1964 Helsinki Declaration and its later amendments. Informed consent was obtained from all individual participants involved in the study. The health facilities of the study consented to the interviews being conducted at their practices.

Results

The p~~P~~articipants recruited for the study were from a variety of general practices and socio-economic localities.~~–~~ A total of 16 healthcare professionals participated in the study, including 7 medical physicians, 7 nurses, and 2 dietitians. To ensure transparency and traceability in the reporting of quotes, each participant was assigned a code reflecting their profession and participant number (e.g., MPh.1 for Male Physician 1, FN.1 for Female Nurse 1, FD.1 for Female Dietitian 1). ~~Sixteen participants completed the study, eleven women and five men; seven were medical physicians, seven were nurses and two were dietitians.~~ The average age of the participants was 37.1 ± 9.8 years. A majority of participants (n = 13) worked only in the public sector, two participants practiced in both the public and private sectors, and one participant practiced only in the private sector. Four themes (Table 1~~2~~) were developed from the analysis.

Table 1~~2~~. Themes developed from IDIs with health professionals.

**Theme**	**Sub-themes**	**Definition**	**Quotes**
**Theme 1. Foundations of dietary understanding**	1.1. Knowledge of nutritional guidelines<br>1.2. Confidence in nutritional counselling	Refers to their understanding of dietary principles, nutritional requirements, and the role of food in promoting health and preventing disease among the population. This knowledge encompasses various aspects including nutritional guidelines, assessment, dietary counselling and nutrition education.	'Studies have shown that with diabetes even if you increase the medication or you optimize the medication, at the end of the day it's the diet that kind of makes or breaks the disease' (FPh.2).<br>'We basically follow the protocols of the National Health Department (…) but it's outdated and it's not up to speed with what's really happening' (MPh.4).<br>'I definitely don't say you need to cut it out completely [sugar] (…) I understand we all do enjoy sweet things here and there' (FD.1).
**Theme 2. LCHF: Controversies and beliefs**	2.1. Awareness of LCHF diets<br>2.2. Concerns over high-fat content	Understanding of health professionals around the recommendations of LCHF diets which depend on their training, experience and personal beliefs.	'I was on [a LCHF diet], but because I didn't have a lot of support (…) I just left it then, but it's actually a good diet (…) I didn't feel so tired' (FN.5).<br>'If you eat high fat things your cholesterol is going to go up,' (FPh.3).

Formatted: Font: Not Italic
Formatted: Font: Not Italic
Formatted: Font: Not Italic
Formatted: Font: Not Italic
Formatted: Font: Not Italic
Formatted: Font: Not Italic
Formatted: Font: Not Italic
Formatted: Font: Bold
Formatted: Font: 8 pt
Formatted Table
Formatted: Font: 8 pt
Formatted: Font: 8 pt
Formatted: Font: 8 pt
Formatted: Font: 8 pt, English (South Africa)

			'I don't advocate a LCHF diet but I'm supportive of what my patients want to try and then I monitor them closely. I watch their cholesterols, their triglycerides, their blood pressures.' (MPh.2).<br>'I wouldn't encourage it [LCHF diet], but if a patient really wanted to do it and ask for my advice, I would try to guide them on it yes, for sure' (FD.2).
**Theme 3. EBSA's role in community nutrition**	3.1. Perceptions of group support<br>3.2. Uncertainty about program sustainability	Health professionals' understanding and perceptions of the EBSA program promoting LCHF within under-resourced communities which encompass EBSA's group support approach, diet recommendations and program's sustainability.	'When you're in a group, then there's more support from one another.' (FN.3).<br>'I think it's a great initiative [EBSA], very necessary because if it's successful, it will definitely reduce the disease burden in our community and that impacts on everything' (FPh.3).<br>'If the people don't have the funding, they're not going to be able to buy it [EBSA's recommended food]' (FPh.7).
**Theme 4: Understanding socioeconomic determinants of health**	4.1. Affordability and food access<br>4.2. Cultural and gender norms shaping diet	Health professionals' understanding of the barriers that under-resourced patients face in accessing healthcare and maintaining good health. Their views on these barriers are informed by their direct interactions with patients, as well as their broader understanding of social determinants of health.	'"Beggars can't be choosers". While they [people from low-income communities] might know that what they're eating is not good for them, they don't have the means to put that into place' (MPh.1).<br>'It's a very big cultural thing to be big [body size]. There's a stigma around it if you're skinny and you're losing weight, you are becoming sick' (FPh.6).<br>'I think in certain cultures, women don't have a lot of say in what gets done, so if the man comes home and he says "he wants this sort of meal" that's what she must prepare.' (FPh.3).
~~**Theme**~~		~~**Definition**~~	~~**Quotes**~~
~~**Theme 1. Foundations of dietary understanding**~~		~~Refers to their understanding of dietary principles, nutritional requirements, and the role of food in promoting health and preventing disease among the population. This knowledge encompasses various aspects including nutritional guidelines, assessment, dietary counselling and nutrition education.~~	~~'The reason why I'm saying I'm confused is I remember 4 or 5 years ago there was a huge promotion of [foods containing] poly-unsaturated fatty acids. Two years ago, poly-unsaturated fatty acids were hailed as causing lots of cancer. So now, you're uncertain which to promote' (Male physician).~~<br>~~'Studies have shown that with diabetes even if you increase the medication or you optimize the medication, at the end of the day it's the diet that kind of makes or breaks the disease' (Female physician).~~<br>~~'We basically follow the protocols of the National Health Department and at the moment, there isn't really much with~~

Formatted: Font: 8 pt

Formatted: Font: 8 pt

Formatted: Font: 8 pt

Formatted: English (United Kingdom)

Formatted: List Paragraph, Outline numbered + Level: 2 + Numbering Style: 1, 2, 3, … + Start at: 1 + Alignment: Left + Aligned at: 0" + Indent at: 0.25"

			regards to prevention, they have information, but it's outdated and it's not up to speed with what's really happening' (Male physician).<br>'I definitely don't say you need to cut it out completely [sugar]. Because I don't think it is realistic. I understand we all do enjoy sweet things here and there' (Female dietitian).
**Theme 2. LCHF: Controversies and beliefs**		Understanding of health professionals around the recommendations of LCHF diets which depend on their training, experience and personal beliefs.	'I was on [a LCHF diet], but because I didn't have a lot of support (…) I just left it then, but it's actually a good diet, I found out, and I didn't feel so tired' (Female nurse).<br>'If you eat high fat things your cholesterol is going to go up (...) Our nutritional education is lacking because you're not taught about the good types of fats and the bad types of fats' (Female physician).<br>'I haven't read all the research about it [LCHF diet]. I know it's very controversial. I know that it's not completely accepted (...) I think it's almost too much of a mindset change for most people' (Male physician).<br>'I don't advocate a LCHF diet but I'm supportive of what my patients want to try and then I monitor them closely. I watch their cholesterols, I watch their triglycerides, I watch their blood pressures.' (Male physician).<br>'I wouldn't encourage it [LCHF diet], but if a patient really wanted to do it and ask for my advice, I would try to guide them on it yes, for sure' (Female dietitian).
**Theme 3. EBSA's role in community nutrition**		Health professionals' understanding and perceptions of the EBSA program promoting LCHF within under-resourced communities which encompass EBSA's group support approach, diet recommendations and program's sustainability.	'When you're in a group, then there's more support from one another. Then you don't feel like, no, I want to eat the chocolate, but now that support comes in' (Female nurse).<br>'I think it's a good thing, especially to educate yourself' (Female nurse).<br>'I think it's a great initiative, very needed, very necessary because it will probably, if it's successful, it will definitely reduce the disease burden in our community and that impacts on everything' (Female physician).<br>'If the people don't have the funding, they're not going to be able to buy it [EBSA's recommended food]' (Female physician).

**Theme 4: Understanding socioeconomic determinants of health**		Health professionals' understanding of the barriers that under-resourced patients face in accessing healthcare and maintaining good health. Their views on these barriers are informed by their direct interactions with patients, as well as their broader understanding of social determinants of health.	'When you're in that state of poverty with 3 to 4 children per household may be 50% of adults unemployed, only 20% of people are living in fixed accommodation as opposed to like" shacks". You kind of get to that expression that beggars can't be choosers, and so while they might know that what they're eating is not good for them, they don't have the means to put that into place' (Male physician).<br>'I think education is the most important thing. Most patients don't know that sweets are bad' (Female dietitian).<br>'It's a very big cultural thing to have, to be big [body size], so it's another thing, there's a stigma around it if you're skinny and you're losing weight, you are becoming sick' (Female physician).<br>'I think in certain cultures, women don't have a lot of say in what gets done, so if the man comes home and he says "he wants this sort of meal" that's what she must prepare. And a lot of the women also aren't educated about contraception, so they end up having lots of children and it's difficult to feed so many mouths with this expensive [LCHF] diet you know' (Female physician).<br>'Some people get addicted to cigarettes, and I wonder if some people get addicted to certain types of food, high fat food from outlet stores or a high carbohydrate diet. I wonder if that stimulates the same sort of mechanism, the same kind of pathway' (Male physician).

LCHF: Low carbohydrate high fat; EBSA: Eat Better South Africa; M: Male; F: Female; Ph: Physician; N: Nurse; D: Dietitian.

Formatted: Font: Not Italic

Formatted: Line spacing: Multiple 1.15 li

Formatted: Font: Not Italic

Theme 1: Foundations of dietary understanding

HThe health professionals recognised that they are not that well-informed regarding about nutritional advice and its role in disease prevention. They felt constrained by time, stating that while nutritional advice is important for disease prevention, they struggle to incorporate it into clinical consultation. Professionals felt that they barely have enough time to see patients for clinical advice and feel that nutritional advice, although important for disease prevention,

~~requires more time. They did,~~ H~~h~~owever, they acknowledged~~d~~ that nutrition plays a crucial role in ~~nutritional advice is ‘vital’ to~~ promoting~~te~~ healthy-diet behaviours ~~so~~ and preventing ~~that~~ diet-related chronic diseases ~~can be addressed~~. There was a general sense of ~~seems to be general~~ confusion regarding nutrition, as participants described encountering ~~in this sample as they mentioned that every second article one reads~~ contradictory dietary information from different sources, making it diffs~~s~~icult to provide clear guidance to patients~~s the previous one (male physician), there is not sufficient data, and they do not keep up with new studies. As a result, professionals find it difficult to deliver nutritional advice~~. As a result ~~it stands~~, they tend to use many “~~‘~~catchphrases”~~’~~ and their own statements of beliefs. This is effectively based on what has traditionally been promoted as healthy behaviour: small portions, reducing~~e~~ red meat, eating fruit and vegetables, drinking water, reducing~~e~~ dietary fats, avoiding unsaturated fats (for instance, removing~~e~~ fat and skin from chickens) and practising~~e~~ physical activity. ~~Some of them produced unsupported statements to explain this advice, and they repeated those statements to their patients.~~

Nurses and physicians often lacked confidence in their own nutritional knowledge and reported feeling uncertain when discussing dietary concepts. ~~frequently came across as lacking confidence in their own nutritional knowledge. Several professionals said they found nutrition confusing, and there was often uncertainty when talking about nutritional concepts.~~ Rather than advising patients themselves, many ~~professionals said they would refer patients to a~~preferred to refer them to dietitian, especially if they felt that the patient required specific nutritional advice. They cited contradictory information from science, media, and government policies as key sources of confusion. ~~They mentioned that a large amount of often contradictory information from science, the media, and the government were often the major sources of the confusion about diet-related diseases.~~ Health professionals highlighted this inconsistency as a challenge for both themselves and their patients, contributing to public uncertainty about

Formatted: Font: Not Italic

Formatted: Font: Not Bold

Formatted: Font: Not Bold

nutrition. ~~the contradictory information as a source of confusion for themselves, their patients and the general public.~~ A few health professionals ~~blamed~~ attributed their lack of ~~self~~ confidence ~~in~~ in providing nutrition advice to insufficient training in both their formal education and continued professional development. ~~this matter on the lack of nutrition education they received during their training and in continued education.~~ Few ~~health professionals~~ participants referenced scientific evidence when justifying dietary recommendations. Therefore, ~~made any reference to science when justifying or explaining their nutritional advice. In those few instances where the health professional did appeal to science, it was in a broad and vague manner.~~ while science was often cited as a source of conflicting information, it was rarely directly referenced by participants. Their confusion appeared to be from inconsistent public health messaging, institutional guidelines, and media influence rather than direct interaction with scientific literature.

Despite not keeping up with new developments in nutrition science, participants agreed that more clinical trials are needed to reassess current dietary guidelines and challenge dietary stigmas, particularly regarding dietary fat~~s~~ and cholesterol levels. Dietary guidance often relied on simplified "catchphrases" (e.g., "balanced diet," "portion control", "sustainable diet") rather than evidence-based specifics. While dietitians emphasized moderation, physicians and nurses were more likely to label foods as "good" or "bad," focusing on reducing sugar, salt, and processed foods. Notably, healthcare professionals rarely referenced scientific literature directly, attributing confusion to conflicting public health messaging rather than engagement with primary research. ~~Despite the fact that they are not able to remain current in the advances in nutrition science, participants have a communal understanding that there is a need for more clinical trials so that the current dietary guidelines may change. They agree that more clinical trials should be conducted to support new dietary trends or to break dietary stigmas, for instance, those regarding dietary fats and its effects on cholesterol levels. The health~~

Formatted: Font: Not Bold

Formatted: Font: Not Italic

Formatted: Font: Not Bold

Formatted: Font: Not Bold

Formatted: Font: Not Bold

Formatted: Font: Not Bold

~~professionals interviewed in this study used a range of jargon and diet statements that were stated as fact, such as 'balanced diet' 'sustainable diet'.~~ These 'catchphrases' can be defined as ~~phrases or~~ statements that are repeated without any justification or attempt to explain them, as if they were obvious. ~~Some of these 'catchphrases' appeared when they had to define a healthy or unhealthy diet. Moreover, several statements appeared when they were asked about the type of advice given to patients, especially from the two dietitians. 'Because it is low carbohydrate, there are certain food groups that they kind of remove and we know that we should be eating all types of vegetables. There shouldn't be one that you should cut out'.~~

Another notable pattern in participant responses was the use of absolutes when discussing dietary beliefs, often presenting certain nutritional claims as self-evident truths. Phrases such as "obviously," "we all know," and "we have known for years" indicated that health professionals regarded certain dietary messages as unquestionable. ~~Another aspect considered in this theme takes into consideration statements about nutrition that provide insight into the beliefs of the health professional. Phrases such as 'obviously', 'we all know', and 'we have known for years' demonstrate that the health professionals hold these nutritional beliefs to be true and beyond doubt. Some nutritional statements were unique, and sometimes quite specific advice was given about a specific food. Some also provided broad nutritional statements without any context, explanation, definition or understanding of the statement being demonstrated. The health professionals said that they have an idea of what are considered problematic foods based on their understanding and seemed to agree on the foods that they consider unhealthy or that are causing medical problems.~~

There was also some consistent advice that was tailored to specific conditions. For instance, low salt for hypertension and portion control and exercise for obesity. The professionals agreed indirectly that healthy food improves immunity;~~,~~ therefore, human immunodeficiency virus (HIV) patients should be encouraged to eat healthy foods because

Formatted: Font: Not Italic

Formatted: Font: Not Italic

Formatted: Font: Not Italic

Formatted: Font: Not Bold

Formatted: Font: Not Bold

Formatted: Font: Not Bold

Formatted: Font: Not Italic

Formatted: Font: Not Italic

Formatted: Font: Not Italic

their immune system is compromised. In general, the cohort thought that good advice would be to promote physical activity, cut out simple sugar, stop or reduce smoking, lower salt intake, reduce alcohol, not to eat spicy food, remove the skin off chicken, remove fat from red meat, and zero sugar sodas instead of regular ones. The above advice was consistent among the professions. Although there were differences in how dietitians, physicians, and nurses approached nutrition advice, there was a shared tendency to frame dietary guidance in practical rather than prescriptive terms. While dietitians emphasized moderation and portion control, physicians and nurses were more likely to label specific foods as problematic. Foods considered detrimental to health included those high in sugar and fat, convenience foods, large portions, starchy foods, sugary drinks, processed snacks, fast foods, and overly salty foods.There were, however, some differences in their approach to nutrition, based on profession. Although there were only two dietitians, the one approach was quite simple and general, namely ‘portion and moderation’. For example, when talking about sugar addiction, it was thought unrealistic to completely exclude sugar from their patients’ diet but to simply reduce it and adapt the diet to the patient's life and circumstances instead.

Physicians and nurses, when advising patients, tend to be more specific by defining some foods as problematic and causative of chronic disease. Of those foods, high sugar and fat content foods were the majority identified as problematic. Examples of such foods were described as: instant or convenience food, inexpensive and easy to access, served in large portions, high-starch content, sugary drinks, ‘chippies and sweeties’, junk food, high- -calorie food, salty food, ‘braai-ed’ [cooked on a barbecue] meat and more. Healthy alternatives were described as vegetables, fruits, small portions, oats, three balanced meals a day, healthy snacks (such as apples or whole-wheat crackers), and avoiding processed foods and sauces. Some professionals also recommended low-fat options, choosing chicken over red meat, and reducing carbohydrate intake. When advising on healthy food alternatives, health professionals

Formatted: Font: Not Bold

Formatted: Font: Not Bold

Formatted: Font: Not Bold

Formatted: Font: Not Bold

Formatted: Font: Not Bold

Formatted: Font: Not Bold

Formatted: Font: Not Italic

Formatted: Font: Not Italic

Formatted: Font: Not Italic

~~referred to the following: vegetables and fruits, small portions, oats, three meals a day, snacks in between (apples, whole-wheat bread with peanut butter and crackers biscuits). Some also mentioned low fat foods, eating chicken instead of red meat and less carbohydrates.~~ Some mentioned that they advised their patients to eat food in their natural state, not too over processed, in moderation and avoid sauces. In general, the advice on practicing physical activity seems to be non-specific and is used when explaining healthy living behaviours and ~~to~~ promot~~e~~inge health.

Theme 2. LCHF~~:~~ c~~C~~ontroversies and beliefs

Almost all the health professionals had heard of the LCHF diet, ~~.~~ though their understanding varied significantly. For those unfamiliar with it,~~For those who did not know about the diet and for clarity for the others, it was defined as:~~ 'A low-carbohydrate high-fat ~~(LCHF)~~ diet is a way of eating that restricts the amount of carbohydrates and increases the number of healthy fats that you eat to optimize your health and nutrition'. ~~The statements on this theme refer to the health professionals talking about their own knowledge of the diet.~~ Some professionals ~~knew~~ recogniszed LCHF diets as the Banting or Tim Noakes diet, as they said that these terms have been popularised over the past few years in South Africa, particularly~~.~~ ~~Most professionals learnt of the LCHF diet through~~ through social media. While some participants correctly described LCHF as a diet promoting whole, unprocessed foods, others had limited knowledge about the specific foods recommended ion the diet. ~~Mostly, they defined LCHF as a diet that promotes unprocessed foods. Some of the cohort had a clear understanding of the diet and the foods that are recommended but most did not.~~ When discussing their opinions on LCHF diets, health professionals frequently used vague, generalized statements such as "long-term health" (Female physician) or "not sustainable" (Female dietitian), indicating uncertainty or a lack of critical engagement with the diet. ~~Regarding their opinions on LCHF diets, many of them mentioned some catch phrases such~~

Formatted: Font: Not Bold

Formatted: Font: Not Bold

Formatted: Font: Not Italic

Formatted: Font: Not Italic

Formatted: Font: Not Italic

Formatted: Font: Not Bold

Formatted: Font: Not Bold

Formatted: Font: Not Bold

~~as: 'long term health' (Female physician) and 'not sustainable' (Female dietitian) as a general understanding of this diet.~~ A ~~relatively large~~ significant number of participants had ~~some~~ personal experience or first-hand knowledge of LCHF diets, with many describing~~.~~ ~~These accounts were predominantly~~ positive outcomes such as needing to eat less, having increased energy levels, and having reduced cravings. However, some stopped following LCHF due to challenges such as a lack of family support or the perception that the diet was expensive. Those who had initially been ~~skeptical~~sceptical of LCHF often noted that it contradicted their formal university nutrition education, particularly regarding the role of fruit, fibere, and starch in the diet.~~, but some explained why they stopped following a LCHF diet, given the potential challenges one may face on a LCHF diet. These include a lack of support from relatives and the perceived expense of it.~~

Public controversy surrounding LCHF further reinforced sckepticism among some health professionals, leading them to express concerns and share their reservations with patients considering or already following the diet. ~~Those who had personally followed the diet mentioned that they experienced that they needed to eat less, had increased energy levels and fewer cravings. These same individuals stated that they were initially sceptical of the LCHF diet, as it contrasted with their university nutrition education. Although some mentioned that according to them the diet limits one from eating what they considered essential foods - such as fruit or fibre -, that one should eat a complete diet, and that the body requires starch. The heightened public controversy surrounding the LCHF diet initially increased the health professionals' hesitancy. They expressed concerns about the diet and reported sharing these concerns with patients who were considering or already following an LCHF diet. Overall, the professionals were~~Their primary~~ily~~ ~~worried~~ concern focused on~~centred around~~ ~~about~~ the high fat content of LCHF diets and whether the diet was financially sustainable for patients from low-income communities. Many professionals believed that LCHF foods ~~were~~are expensive,

Formatted: Font: Not Italic

Formatted: Font: Not Bold

Formatted: Font: Not Bold

Formatted: Font: Not Bold

Formatted: Font: Not Bold

Formatted: Font: Not Bold

Formatted: Font: Not Bold

Formatted: Font: Not Bold

Formatted: Font: Not Bold

Formatted: Font: Not Bold

Formatted: Font: Not Bold

Formatted: Font: Not Bold

making it difficult for patients to maintain the dieta long-term diet. . Some also noted that sustaining the diet could be challenging due to the high cost of LCHF foods, leading to the common perception that the diet is expensive and therefore difficult for many patients to maintain. On average, theySome were prepared willing to support patients who chose to follow LCHF, but their support was often conditional on closely be supportive of the diets but would monitoring patients to see if there are any harmful for potential negative effects. While international consensus guidelines recognize carbohydrate restriction as an effective approach for managing type 2 diabetes, scepticism remains among some health professionals, particularly regarding its long-term sustainability and applicability in under-resourced settings. In Ssome of the health professionals questioned whether LCHF could effectively treat metabolic disorders, largely due to ongoing controversy surrounding the diet and the lack of there was a high degree of scepticism around the efficacy of LCHF to treat metabolic disorders, largely due to the controversy surrounding the LCHF diet and a lack of full scientific agreement on its long-term effects.scientific consensus. Their main concerns included the sustainability of the diet, its affordability, the high intake of meat, the restriction of carbohydrates, and, most notably, its fat content dueowing to the perceived link between fat intake and cholesterol levels. Some professionals acknowledged that their views on cholesterol and dietary fat might be outdated, attributing this to limited access to updated nutrition science.Specifically, some common factors that made them sceptical were the sustainability of the diet, the affordability, the amount of meat, the exclusion of an entire macronutrient (‘no carbohydrate at all’) and, mostly, the fat intake due to the apparent link to cholesterol levels. Although, some mentioned that they were aware that their perceptions regarding cholesterol levels and dietary fats might be outdated since they did not have access to enough nutrition information.

Formatted: Font: Not Bold

Formatted: Font: Not Bold

Formatted: Font: Not Bold

Formatted: Font: Not Bold

Formatted: Font: Not Bold

Formatted: Font: Not Bold

Formatted: Font: Not Bold

Formatted: Font: Not Bold

Health professionals also expressed concerns about patients' ability to fully understand and follow LCHF correctly, fearing that misinformation and a lack of nutritional literacy could lead to poor adherence or unintended health risks. Some worried that patients would struggle to transition to a low-carb diet, particularly without professional guidance and ongoing monitoring. When asked whether they would introduce LCHF to treat or prevent metabolic conditions, most stated that they would support patients who chose it but would not actively advocate for the diet due to the concerns outlined above. This cautious stance led many professionals to adopt a "wait-and-see" approach, where they would monitor patients rather than actively encourage the diet.

Some alternative reasons for their reluctance included the belief that LCHF is too restrictive and that vegetables should not be excluded from a healthy diet. Many professionals also believed that patients lacked adequate knowledge about their own health before adopting LCHF, further reducing their willingness to endorse the diet. The two dietitians interviewed highlighted adherence as a key challenge, stating that while LCHF may be effective for some individuals, an individualized dietary approach may be more appropriate. As a result, they did not advocate for LCHF as a universal dietary solution, emphasizing instead that dietary interventions should be tailored to each patient's needs.

Moreover, some were concerned that patients do not really understand the diet, that they lack information and that it would be difficult for patients to change their dietary behaviours to this diet and to monitor patients on this diet. When asked about introducing a LCHF diet to treat or prevent metabolic conditions, health professionals said that they would support it but not advocate it due to the above mentioned concerns. This led them to a cautious and vigilant attitude regarding their patients who followed a LCHF diet. Alternative reasons for this attitude include beliefs that the LCHF diet is a restrictive diet and that no vegetables should be cut out of a nutritional plan. The professionals said that they believed that their

Formatted: Font: Not Bold

Formatted: Font: Not Bold

Formatted: Font: Not Bold

Formatted: Font: Not Bold

Formatted: Font: Not Bold

Formatted: Font: Not Bold

Formatted: Font: Not Bold

Formatted: Font: Not Bold

Formatted: Font: Not Bold

Formatted: Font: Not Bold

Formatted: Font: Not Bold

Formatted: Font: Not Bold

Formatted: Font: Not Bold

Formatted: Font: Not Bold

Formatted: Font: Not Bold

Formatted: Font: Not Bold

Formatted: Font: Not Bold

~~patients do not have enough knowledge about their own health prior to embarking on a LCHF diet – further reducing their willingness to advocate. The two dietitians mentioned that adherence is a problem and that the most effective nutritional approach could be LCHF or a individualised one. Hence, they do not advocate LCHF diets as they do not consider it applicable to everyone.~~

Theme 3: EBSA's role in community nutrition

Some of the interviewees were familiar with the EBSA program, but the majority were introduced to it ~~to the program~~ for the first time during the interview. Those who ~~knew~~ were aware ~~abou~~of~~t~~ EBSA had patients who had participated in the program. For clarity, t~~T~~he EBSA program was defined to all participants as: 'Eat Better South Africa is a nutrition education program for people from under-resourced communities that promotes low-carbohydrate high-fat diet or Banting diet to prevent or treat metabolic conditions. It consists of groups of people that meet every week for an educational session for a period of six weeks'.

Many professionals viewed group support as essential for dietary interventions, stating that changing lifestyle behaviours is easier when done collectively rather than individually. Some participants saw EBSA as a positive initiative for reshaping their dietary mindset~~s~~ and recognis~~z~~ed its potential to alleviate pressure on healthcare facilities by reducing the incidence of diet-related chronic diseases. However, concerns about the EBSA program often overlapped with broader concerns about LCHF diets, particularly in terms of sustainability, affordability, and accessibility in low-income communities. Health professionals also emphasized the need for structured nutrition education programs tailored for healthcare workers themselves. Many expressed that they lacked the necessary tools and training to provide effective dietary guidance and stated that a program similar to EBSA, designed for health professionals, would help them better address nutrition-related diseases in their practice. While the majority of participants stated that they would support patients participating in EBSA, they specified the limits of their

Formatted: Font: Not Italic

Formatted: Font: Not Italic

Formatted: Font: Not Bold (×15)

support, indicating that they would monitor but not necessarily promote the program’s dietary approach.

Despite these reservations, group support was considered the most valuable aspect of the EBSA program. Many professionals agreed that individual behaviour change is difficult, and that patients are more likely to succeed when they receive support from their peers. Some participants valued the educational aspect of EBSA, stating that if more at-risk individuals joined similar community-based nutrition programs, the burden of chronic diseases on hospitals could be reduced. Concerns about EBSA primarily mirrored existing concerns about LCHF diets, with professionals questioning whether the diet could be adapted to the financial and food accessibility challenges of under-resourced communities. Some interviewees stressed the importance of tailoring dietary recommendations to local food prices and availability, ensuring that any dietary intervention remains affordable and practical for long-term adherence. Several health professionals also provided suggestions on how EBSA could better address food affordability and sustainability, indicating a willingness to engage with the program if these issues were explicitly considered in its implementation.

This theme involves the thoughts and opinions provided by the health professionals specific to the EBSA program. Interviewees mostly highlighted the importance of group support to successfully achieve a lifestyle behaviour change. Some believed it to be a good initiative to change the way people think, some concerns regarding the program are also related to concerns about LCHF diets. The concept of EBSA stimulated stress on the importance of support for the healthcare system both inside and outside of hospital facilities. They also stated that health professionals would appreciate having a similar nutrition education program to give them the tools to better address diet-related diseases. The majority of participants said they would support the patient but were specific about how they would offer this support:

Formatted: Font: Not Bold

Formatted: Font: Not Bold

Formatted: Font: Not Bold

Formatted: Font: Not Bold

Formatted: Font: Not Bold

Formatted: Font: Not Bold

Formatted: Font: Not Bold

Formatted: Font: Not Bold

Formatted: Font: Not Bold

Formatted: Font: Not Bold

Formatted: Font: Not Bold

Formatted: Font: Not Bold

Formatted: Font: Not Bold

~~Despite this attitude towards professional patient support, group support was considered the most valuable aspect of the EBSA program. In their opinion, behaviour change is not easy to achieve as an individual, and it is easier in a group. Some professionals valued the education aspect of the EBSA program. They indicated that, if more unhealthy people participated in support groups such as EBSA, there would be less pressure on hospital facilities from chronic diseases. EBSA was seen as a good initiative, and that it could change the way people think. The only concerns regarding the EBSA program were those related to the LCHF diet that were mentioned before. There were suggestions for community implementation and adherence to a LCHF diet – that it needs to be tailored to food prices, accessibility and sustainability. They stated that it is necessary to ask whether people can afford it and what the food options are in their communities and surroundings because it has to be sustainable. Some health professionals even provided suggestions that EBSA could incorporate, regarding the food prices and how to approach the sustainability of the programs in communities where poverty is a common challenge.~~

Theme 4: Understanding socioeconomic determinants of health

~~This theme emerged from questions pertaining to reasons why people from poor communities (patients) eat in the way they eat. The~~Health professionals broadly acknowledged the ~~spoke in general about the~~ challenges that people living in under-resourced communities face, demonstrating awareness of the socioeconomic barriers to healthy living.- ~~. Thus, they are quite aware of the community challenges to healthy living.~~ Many participants referred to specific communities such as Ocean View and Masiphumelele, which are often highlighted in the media for high crime rates, violence, and safety concerns. Some health professionals reside in the same communities as their patients do, providing them with firsthand insight into the rationale behind food choices and the structural challenges that shape dietary behaviours.~~They specifically mentioned those related to poverty and the social determinants of health found in~~

Formatted: Font: Not Bold

Formatted: Font: Not Bold

Formatted: Font: Not Bold

Formatted: Font: Not Bold

~~communities such as Ocean View or Masiphumelele. Some of the health professionals interviewed live in the same communities as their patients. They have great insight into the rationale behind food choices and the challenges that patients face. These communities are commonly known, usually appearing in the media because of crime, violence and safety concerns.~~ ~~Moreover, t~~This theme captures interconnected issues related to food ~~is also a combination of those codes related to~~ accessibility, affordability, safety, and knowledge. ~~understanding and safety regarding food and food resources.~~ S~~ome~~everal health professionals ~~acknowledged~~ noted that for many individuals, food is not a priority, and that they ~~that~~ '*Food* ~~*is not a priority*, (Male physician)' explaining that people from under-resourced communities~~ are in a survival mode. The ~~price~~ cost of food was often cited as an issue, emphasizing that healthier~~y~~ foods tend to be ~~are~~ more expensive than ~~unhealthy food~~processed and unhealthy options. Meat was consistently described as expensive, though some also noted that vegetables and other fresh produce were increasingly costly, further complicating efforts to promote healthier dietary choices.~~Specifically, that meat is extremely expensive, but others pointed out that vegetables are also expensive.~~

The interviewees cited poverty and~~or~~ lack of resources due to socio-economic circumstances as a challenge to healthy lifestyle choices and changes. According to the information that the participants provided, c~~C~~ommunities such as Ocean View and Masiphumelele face high levels of unemployment, crime, inadequate housing, and limited access to healthcare and nutrition education.~~are considered under-resourced communities with a high rate of unemployment, gangsterism, lack of resources, lack of housing and limited access to health and proper nutrition education [48,49].~~ Unemployment appeared to be a major contributing factor to the high level of poverty in these communities. Health p~~P~~rofessionals also considered limited education a barrier ~~a lack of education as an obstacle~~ to adopting

Formatted: Font: Not Italic

Formatted: Font: Not Italic

Formatted: Font: Not Bold

Formatted: Font: Not Bold

healthy lifestyle choices, changes, or nutritional interventions. They recognised that nutrition education could have a strong influence on changing unhealthy habits and improving long-term health outcomes~~a huge beneficial influence on unhealthy habits~~. Alcohol abuse was mentioned as a contributing factor to both crime and the reinforcement of unhealthy behaviours, which further complicated adherence to medical and lifestyle recommendations. ~~few times during the interviews and the health professionals said that it can be a problem for some people, as it leads to increased rates of crime in addition to perpetuating unhealthy habits and poor adherence.~~ Compliance with prescribed medication, treatment, and lifestyle advice was ~~to the medication/treatment/lifestyle advice was~~ identified as a challenge for the patients from the health professionals' perspective.

Health professionals acknowledged that culture and tradition strongly influence dietary habits and perceptions of health, which are often shaped by family and community norms. ~~culture and tradition around food and nutrition has a significant role in a patient's lifestyle choices and that this is heavily influenced by their upbringing.~~ Cultural stigmatization around body weight and physical appearance was a recurring theme, particularly regarding women.~~occurs around weight and physical appearance, especially for women.~~ Some participants noted that being s~~S~~lim was sometimes associated with poverty and malnutrition, whereas larger body sizes were perceived as indicators of wealth and well-being. ~~people are regarded as being poor, malnourished and unhealthy, yet large to obese people are seen as wealthy and healthy [50].~~ Gender roles within households were also highlighted, as women were often responsible for preparing meals yet had to navigate traditional expectations that limited their decision-making power regarding food choices, health, and financial autonomy. ~~The health professionals stated that there are gender differences in the communities living conditions. The women's roles in the community and household were also highlighted – women are usually responsible for cooking yet have to deal with gender role challenges [51]. Some women would have to manage~~

Formatted: Font: Not Bold

Formatted: Font: Not Bold

Formatted: Font: Not Bold

Formatted: Font: Not Bold

household duties while navigating traditional expectations that limit their decision-making power, particularly regarding health and economic autonomy [51]. Another key theme was the diverse cultural backgrounds within these communities, which influenced dietary habits and nutritional choices. Some health professionals discussed how migration from rural to urban areas impacted dietary behaviours. In rural settings, individuals often had access to fresh, homegrown food and engaged in more physical activity. However, upon relocating to urban environments, they tended to maintain a high-carbohydrate diet while adopting more sedentary lifestyles, contributing to diet-related health concerns. The transition from rural to urban living was perceived as a challenge, as individuals often lost access to traditional food sources such as home gardening or farming, increasing their reliance on processed and nutrient-poor foods. Another aspect that appeared to be important according to health professionals was that these communities are made up of different people from diverse races and cultures. Relocation from rural areas to cities for greater job opportunities was considered a potential problem regarding food habits. When people relocate, especially from rural to more urban environments, there is less opportunity to move and less physical work, yet they want to eat the same diet. This diet is usually high in carbohydrates, but in rural areas people tend to have larger and healthier sources of food through gardening or farming. When they come to the cities, they tend to eat more processed foods and poorly [52].

Health professionals noted that despite receiving nutritional advice, many patients do not adhere to dietary recommendations, often due to aversion to unfamiliar foods or deeply ingrained eating habits. They identified a preference for ultra-processed foods, explaining that these foods are not only widely available but also enjoyed for their taste, making compliance with healthier dietary guidance more challenging. Some professionals linked this preference to food addiction and eating disorders, highlighting the difficulty in breaking habitual

Formatted: Font: Not Bold

Formatted: Font: Not Bold

Formatted: Font: Not Bold

Formatted: Font: Not Bold

Formatted: English (South Africa)

Formatted: Font: Not Bold

Formatted: Font: Not Bold

Formatted: Font: Not Bold

Formatted: Font: Not Bold

Formatted: Font: Not Bold

Formatted: Font: Not Bold

consumption patterns of processed, high-sugar foods. An important cultural aspect raised by professionals was the link between food and social status in under-resourced communities. People with limited financial resources may prioritize purchasing branded, ultra-processed foods as a way to signal wealth or social belonging, even if those foods are nutritionally poor. Professionals observed that certain food choices were not solely based on affordability but also on perceptions of prestige and aspiration, further complicating efforts to promote healthier eating habits.

When talking about traditional ways of eating or upbringing, the health professionals found that, despite dispensing nutritional advice, patients do not follow the recommendations. They spoke about a lack of compliance from patients who were largely averse to different foods. The health professionals recognise that unhealthy ultra-processed foods are being eaten because they are enjoyable. This does not aid in compliance to dietary advice or education and ties in with food addiction and eating disorders as medical conditions. An interesting aspect of the culture in these communities mentioned by health professionals is one of food and status. According to them, people who lack resources desire to appear as if they can afford foods that they cannot, and there is a certain status attached to those ultra-processed foods from specific well-known brands.

Discussion

This study is the first to explore health professionals' perceptions of a community-based program promoting LCHF diets in under-resourced South African communities. The findings reveal a general alignment with conventional dietary guidelines among health professionals, who emphasize the importance of vegetables and fruits while expressing concerns about dietary fats and meat [4953]. While physicians and nurses recognized the value of nutritional education in disease prevention, their ability to provide effective dietary counselling was hindered by systemic barriers, including time constraints, competing clinical responsibilities, and

Formatted: Font: Not Bold

Formatted: Font: Not Bold

Formatted: Font: Not Bold

Formatted: Font: Not Bold

Formatted: Font: Not Bold

Formatted: Indent: Left: 0", Hanging: 0.5"

inconsistent nutritional messaging. Physicians and nurses acknowledged their limited expertise in nutrition but recognized the critical role of nutritional education in disease prevention. However, they also highlighted the challenges of staying updated with evolving nutritional science and expressed uncertainty in providing dietary guidance due to the constant influx of new information [5054]. This reflects a broader issue of confusion and inconsistency in nutritional advice, which can undermine the effectiveness of health promotion efforts.

The study also found that different healthcare professionalss approach nutrition advice differently. Physicians admitted to having some nutritional knowledge but lacked confidence in offering dietary guidance, while nurses, who are often the primary healthcare providers, demonstrated more openness to new information and a greater willingness to provide lifestyle counselling, particularly in managing non-communicable diseases (NCDs) [515-559]. However, their ability to provide effective dietary counselling was limited by time constraints, competing responsibilities, and a lack of structured training. An in-depth qualitative investigation with nurses delved into their encounters while counselling diabetes patients on diet, exercise, and smoking cessation [559]. Nurses highlighted challenges like patients' limited knowledge and motivation, as well as their own counselling skills and time constraints, hindering effective lifestyle counselling. Despite these obstacles, the study revealed nurses' readiness to offer weight management advice as part of their professional duties, aligning with previous research findings [548, 559]. Dietitians, though fewer in number, adhered more strictly to existing dietary guidelines and were cautious about endorsing LCHF diets due to concerns about their sustainability and potential health risks [560].

While health professionals expressed difficulty in staying updated with advancements in nutrition science, they also relied heavily on widely accepted dietary principles without necessarily questioning or critically analysyzing them. Furthermore, many professionals agreed that more clinical trials were needed to reassess current dietary guidelines, reflecting an

Formatted: Font: Not Bold

Formatted: Font: Not Bold

awareness of the evolving nature of nutrition science. This duality, acknowledging the need for stronger evidence while continuing to use conventional dietary rhetoric, highlights the complexity of how professionals navigate nutritional knowledge in practice.

Despite the cautious approach of most health professionals, there was an acknowledgment of the potential benefits of LCHF diets in clinical nutrition, especially if patients are closely monitored. However, concerns about practical challenges in implementing ~~the~~ diets were prevalent, including ~~controversies surrounding~~the cost and variety of ~~meat and dietary fat intake, the cost and variety of~~ recommended foods, the feasibility of adherence in low-income settings, and the alignment with national dietary guidelines were prevalent [34, 57~~61~~, 58~~62~~]. Health professionals generally viewed these barriers as more pressing than the debate over the diet's health effects, suggesting that practical implementation challenges could be the primary concern. ~~The concerns raised by most of the health professionals were linked to the national guideline recommendations that have been recently questioned in recent scientific reviews [62].~~ While most health professionals claimed that they would support patients, their approach often inadvertently discouraged them, resembling "backhanded support", explicitly noted in some instances. Their reluctance stemmed from concerns about the diet's alignment with conventional dietary guidelines, a lack of confidence in providing LCHF-specific advice, and uncertainty about its long-term health impacts. Rather than actively endorsing LCHF diets, professionals supported patients out of a desire to respect patient autonomy, a stance known as therapeutic pragmatism, where healthcare providers adopt a supportive role despite personal doubts [59]. This meant that while they outwardly agreed to assist patients following an LCHF approach, their hesitance and underlying scepticism often resulted in a lack of meaningful guidance, leaving patients to navigate dietary changes largely on their own. Alternatively, physicians and nurses sometimes directed patients to dietitians, who, as revealed in interviews,

Formatted: Font: Not Bold

Formatted: Font: Not Bold

Formatted: Font:

Formatted: Font: Not Bold

Formatted: Font: Not Bold

might dissuade them from LCHF diets in favour of a more balanced macronutrient approach~~, as revealed in interviews~~.

Some study participants had personal experiences with or first-hand knowledge of LCHF diets from friends or family, which tended to be positive. Interestingly, despite their cautiousness in endorsing or advocating for the diet, some had personally experimented with it and experienced favourable results, reflecting findings from other ~~literature where~~studies in which individuals reported improved well-being, weight loss, and reduced hunger i~~o~~n the diet [60~~3~~-63~~7~~]. When discussing the LCHF diet and introducing the EBSA program as a nutrition and health education initiative promoting this approach, health professionals showed interest in the concept of group support, suggesting potential backing for community-based programs that provide such support. The availability of group support within the program was seen as a catalyst for encouraging dietary behaviour changes. However, this interest did not necessarily translate into full endorsement of the LCHF diet in communities, as concerns persisted about various barriers to implementation, including food costs, accessibility, and conflicting advice from different health workers. While LCHF diets are widely recognized in South Africa, their popularity is largely driven by media visibility and public discourse rather than strong institutional or professional endorsement. Many healthcare professionals remained cautious about formally supporting the diet, reflecting a broader global trend where media-driven interest does not always translate into clinical adoption [60]. This suggests that scepticism about LCHF is not solely a matter of questioning its health implications but rather an uncertainty about how it can be practically adopted in resource-limited settings. ~~controversies, gaps in education, and socioeconomic challenges like poverty.~~ Some professionals also noted that hesitation toward LCHF did not come solely from healthcare providers but was also present within the support groups themselves, which were composed of community members, including patients. This reluctance within the groups stemmed from conflicting advice received

Formatted: Font: Not Bold

Formatted: Font: Not Bold

Formatted: Font: Not Bold

from different health workers, scepticism about non-traditional diets, and broader uncertainty about the feasibility of LCHF within low-income settings [61-63]. While some professionals acknowledged that the program might be effective in changing perceptions about diet, concerns about the LCHF approach were also raised.

There was a general recognition of the challenging environmental conditions in under-resourced South African communities, with food insecurity emerging as a significant concern due to issues of accessibility and affordability. Therefore, socioeconomic determinants emerged as critical barriers to dietary change. Additionally, although not explicitly highlighted, discussions also touched on women's safety concerns. Cultural influences and traditional dietary habits were frequently mentioned, echoing previous research [648-6670]. These factors were seen as obstacles to both individual and community well-being. Health professionals often framed these challenges as significant barriers to dietary change rather than outright rejecting LCHF as a viable nutritional intervention. ~~as justifications for patients' difficulties in adhering to medical advice, reflecting a tendency to downplay patients' aspirations for better health.~~ This rationalization of limited intervention in nutritional guidance aligns with previous observations [504]. The findings of this study highlight how culture, tradition, and socioeconomic status influence dietary behaviours and perceptions of health, often complicating the effectiveness of nutritional education and dietary interventions. The association between larger body sizes and wealth, as noted by health professionals, aligns with previous research showing that body image norms in some South African communities equate size with prosperity and well-being, while thinness is often linked to illness or poverty [67]. These perceptions, combined with gendered household roles, place women in a position where they are responsible for meal preparation but have limited autonomy in making food choices, reinforcing structural barriers to dietary change [68]. Additionally, the migration from rural to urban areas has disrupted traditional food systems, increasing reliance on processed and

Formatted: Font: Not Bold

commercially available foods, which mirrors broader trends in the African nutrition transition [69].

Health professionals also expressed frustration over patients' low compliance with dietary recommendations, with many preferring familiar ultra-processed foods that are perceived as enjoyable and, in some cases, status symbols. This echoes previous research highlighting the role of food choices as social markers in economically constrained environments, where individuals may prioritize brand-associated foods as symbols of affluence despite financial hardship [70]. These findings echo broader research on nutrition transitions in Africa, where urbanization and economic constraints drive reliance on processed foods [69]. Understanding these complex social and cultural influences is critical for designing effective, context-specific nutrition education programs that resonate with communities while addressing underlying barriers to dietary change. This perspective connects with the notion of "backhanded support" when it comes to assisting patients interested in following an LCHF diet. Health professionals acknowledged that unhealthy foods are often consumed because they are enjoyable [5~~1~~5,~~56~~60,71-73], linking this to concepts of food addiction and predictors of problematic eating behaviours. However, many professionals believed that economic factors and social influences played a more decisive role than individual dietary preferences in shaping food choices. Notably, healthcare providers, particularly dietitians, appeared hesitant to challenge these preferences among participants.

This study has a temporal limitation, as data collection took place between November 2019 and February 2020, meaning that the findings capture a specific moment in time. Dietary discourse in South Africa may have evolved since then, particularly due to pandemic-related shifts in food access, nutrition policies, and public health priorities. While thise study provides valuable insights into health professionals' perspectives, future research is needed to explore how these views may have changed with emerging dietary trends and transition initiatives.

Formatted: Font: Not Bold

Based on the study findings, EBSA could improve their program by offering targeted training for healthcare providers, particularly nurses and physicians, to boost their confidence in recommending LCHF diets. This training should address misconceptions about dietary fats and provide strategies for monitoring patients effectively. Additionally, EBSA could develop resources that consider the socioeconomic challenges and cultural influences in under-resourced communities, offering practical and affordable dietary advice. Enhancing the group support component could also strengthen community involvement and help sustain dietary changes, leading to better health outcomes. This study is a pioneering effort to explore health professionals' views on a community-based program promoting LCHF diets in underprivileged South African communities.

~~Through ~~Q~~q~~ualitative interviews~~, it~~ revealed~~s~~ that health professionals largely align with conventional dietary guidelines, emphasizing vegetables and fruits while expressing concerns about fats and meat. The diverse perspectives of physicians, nurses, and dietitians offer a comprehensive view, but the uneven sample size, limited facility access, ~~and ~~the affiliation of one dietitian with an NGO, and the exclusion of non-English speakers may have limited the diversity of perspectives captured and ~~may ~~introduced biases. Future studies should include non-English-speaking health professionals to capture broader cultural perspectives. Additionally, while dietitians are typically the primary providers of dietary counselling, in under-resourced primary care settings, nurses and physicians often take on this role due to staff shortages [52]. This reflects the real-world task-shifting that is common in South African healthcare, where non-specialist providers must frequently step in to deliver nutritional advice. While dietitians play a central role in dietary counselling, this study included physicians and nurses to capture how nutrition messages are communicated in primary care, aligning with community health models for under-resourced settings. Future research focusing solely on dietitians could provide more specialized insights into LCHF counselling and implementation.

By highlighting the crucial role of nurses in health promotion, thise study underscores the need for further research to explore how task-shifting impacts dietary counselling and to address the broader systemic challenges influencing healthcare practices. ~~While highlighting the crucial role of nurses in health promotion, the study suggests that further research is needed to address its limitations and deepen our understanding of the complex factors influencing healthcare practices.~~ Despite these challenges, the study offers valuable insights into health professionals' attitudes towards nutritional advice and LCHF diets, contributing to discussions on community healthcare and NCD management. Notably, some doctors privately believed in LCHF's potential benefits—often informed more by media than by formal training—but were reluctant to refer patients to dietitians, anticipating that dietitians would discourage LCHF in favour of conventional low-fat advice [13].

Conclusions

This study provides valuable insights into healthcare professionals' perspectives on a nutrition education program recommending LCHF diets, highlighting key challenges in their understanding and implementation within under-resourced South African communities. ~~P~~The professionals expressed uncertainty around LCHF, which was often shaped by conflicting guidelines, limited exposure to recent evidence, and scepticism about its long-term effects. The findings also reveal significant training gaps in nutrition education, with many professionals, mostly doctors and nurses, lacking confidence in providing dietary guidance beyond conventional recommendations. Additionally, socioeconomic barriers such as food affordability, cultural norms, and structural inequalities further complicate dietary behaviour changes in these communities. Despite these challenges, according to participants, EBSA presents a promising model for integrating nutrition education into healthcare strategies, particularly by offering structured support and practical guidance tailored to local contexts. Future efforts should focus on bridging knowledge gaps, strengthening professional training,

and ensuring that dietary interventions are both evidence-based and accessible to those who need them most.The scientific controversy surrounding LCHF diets reflects broader challenges in dietary interventions and the limitations of nutritional epidemiology. Health professionals generally exhibited a supportive but non-advocating stance towards LCHF diets, reflecting the ongoing debate and uncertainty in clinical practice regarding therapeutic nutritional interventions. Given the popularity of LCHF diets in South Africa, healthcare professionals are likely to encounter patients interested in or already following such diets. While international guidelines increasingly endorse LCHF diets, confusion and knowledge gaps persist, particularly concerning patients with complex health conditions. For these patients, including those on multiple medications or with comorbidities like renal disease, LCHF diets should be supported by experienced healthcare professionals to ensure optimal nutritional intake. Although long-term compliance and sustainability of carbohydrate restriction remain uncertain, existing evidence suggests that LCHF diets can be a legitimate and potentially effective option for managing diet-related chronic diseases.

Acknowledgments

We acknowledge the health professionals who volunteered to participate in this study, and Cyber Transcription for transcriptions. Thank you to the hospitals and non-profit organizations for allowing the interviews to be conducted in their facilities.

Data availability

Formatted: Not Highlight

The full transcripts and raw data from this study cannot be shared publicly due to ethical and legal restrictions. The research involved interviews with healthcare professionals discussing potentially sensitive information related to their workplace, patients, and professional practices. The approved protocol, which was reviewed by the Human Research

Ethics Committee of the University of Cape Town (HREC REF 512/2019), did not include provisions for public data sharing. Participants consented to participate in the study under the condition that their full transcripts and identifying information would remain confidential and would not be made publicly available. However, researchers who meet the criteria for access to confidential data may request access to de-identified datasets by contacting the Human Research Ethics Committee of the University of Cape Town (Hub-PhysiologicalSciences@uct.ac.za) or the P.I. Georgina Pujol-Busquets (georgipbg@uoc.edu). ~~Data cannot be shared publicly as participants did not give consent for their transcripts to be shared in this manner. Since the consent statement approved by the Human Research Ethics Committee of the University of Cape Town, and signed by the participants, did not include the provision that data would be made publicly available, we do not have participant consent to share this data. Requests for anonymized data can be made to the Principal Investigator of the study, Georgina Pujol-Busquets (georgipbg@uoc.edu).~~

Formatted: Not Highlight

Formatted: Not Highlight

Formatted: Not Highlight

Conflicts of i~~I~~nterest

The authors declare no conflicts of interest. G.P.B. is a recipient of the Tim and Marilyn Noakes postdoctoral fellowship. The funders had no role in the design of the study; in the collection, analysis, or interpretation of data; in the writing of the manuscript; or in the decision to publish the results.

References

1. The Constitution and public health policy. Chapter 2. Health & Democracy. [Internet]. [cited 2024 Mar 17]. Available from: https://section27.org.za/wp-content/uploads/2010/04/Chapter2.pdf

2. Maphumulo WT, Bhengu BR. Challenges of quality improvement in the healthcare of South Africa post-apartheid: A critical review. Curationis. 2019;42(1):1–9. doi:10.4102/curationis.v42i1.1901.
3. Stuckler D, Basu S, Mckee M. Health care capacity and allocations among South Africa's provinces: Infrastructure-inequality traps after the end of apartheid. Am J Public Health. 2011;101(1):165–72. doi:10.2105/AJPH.2009.184895.
4. Baker A. What South Africa can teach us as worldwide inequality grows. Time. 2019;1. [Internet]. [cited 2024 Mar 17]. Available from: https://time.com/longform/south-africa-unequal-country/
5. Oladipo JA. Utilization of health care services in rural and urban areas: A determinant factor in planning and managing health care delivery systems. Afr Health Sci. 2014;14(2):322-33. doi:10.4314/ahs.v14i2.6.
6. Turok I. Urbanisation and development in South Africa: Economic imperatives, spatial distortions and strategic responses. Urbanisation and Emerging Population Issues: Working Paper; 8. London: International Institute for Environment and Development (IIED). [Internet]. [cited 2024 Mar 17]. Available from: http://hdl.handle.net/20.500.11910/3082.
7. Kon ZR, Lackan N. Ethnic disparities in access to care in post-apartheid South Africa. Am J Public Health. 2008;98(12):2272–7. doi: 10.2105/AJPH.2007.127829.
8. Voget U. Professional nurses' lived experiences of moral distress at a district hospital. [Master's Thesis]. Stellenbosch University, South Africa; 2017. Available from: https://core.ac.uk/download/pdf/188221241.pdf.
9. Coovadia H, Jewkes R, Barron P, Sanders D, McIntyre D. The health and health system of South Africa: Historical roots of current public health challenges. Lancet. 2009;374(9692):817–34. doi:10.1016/S0140-6736(09)60951-X.

10. Kruger HS, Puoane T, Senekal M, van der Merwe M-T. Obesity in South Africa: Challenges for government and health professionals. Public Health Nutr. 2005;8(5):491–500. doi: 10.1079/phn2005785.
11. Mayosi BM, Lawn JE, Niekerk A van, Bradshaw D, Karim SSA, Coovadia HM, et al. Health in South Africa: Changes and challenges since 2009. Lancet. 2012;380(9858):5–19. doi: 10.1016/S0140-6736(12)61814-5.
12. Sanders D, Chopra M. Key challenges to achieving health for all in an inequitable society: The case of South Africa. Am J Public Health. 2006;96(1):73–8. doi: 10.2105/AJPH.2005.062679.
13. Crowley J, Ball L, Hiddink GJ. Nutrition in medical education: A systematic review. Lancet Planet Health. 2019;3(9). doi: 10.1016/S2542-5196(19)30171-8.
14. Gallegos D. Global challenges and opportunities for dietitians. Nutr Diet. 2020;77:179-81. doi: 10.1111/1747-0080.12614.
15. Kohlmeier M, Nowson CA, DiMaria-Ghalili RA, Ray S. Nutrition education for the health care professions. J Biomed Educ. 2015;2015:1–2. doi: 10.1155/2015/380917.
16. Köse S, Yildirim G. The effect of a nutrition education. Int J Caring Sci. 2020;13(6):573–82. [Internet]. [cited 2024 Mar 17]. Available from: https://www.internationaljournalofcaringsciences.org/docs/63_selmin_%20original_13_1.pdf
17. Riddell MA, Edwards N, Thompson SR, Bernabe-Ortiz A, Praveen D, Johnson C, et al. Developing consensus measures for global programs: Lessons from the Global Alliance for Chronic Diseases Hypertension research program. Global Health. 2017;13(1):1–12. doi:10.1186/s12992-017-0242-8.

18. Popkin BM. Global nutrition dynamics: The world is shifting rapidly toward a diet linked with noncommunicable diseases. Am J Clin Nutr. 2006;84(2):289-98. doi: 10.1093/ajcn/84.1.289.
19. Beckfield J, Olafsdottir S, Bakhtiari E. Health inequalities in global context. Am Behav Sci. 2013;57(8):1014-1039. doi: 10.1177/0002764213487343.
20. Perez-Escamilla R, Bermudez O, Buccini GS, Kumanyika S, Lutter CK, Monsivais P, et al. Nutrition disparities and the global burden of malnutrition. BMJ. 2018;361:k2252. doi: 10.1136/bmj.k2252.
21. Kris-Etherton PM, Akabas SR, Douglas P, et al. Nutrition competencies in health professionals' education and training: A new paradigm. Adv Nutr. 2015;6(1):83–87. doi:10.3945/an.114.006734.
22. Viswanath K, Bond K. Social determinants and nutrition: Reflections on the role of communication. J Nutr Educ Behav. 2007;39(2). doi: 10.1016/j.jneb.2006.07.008.
23. Burgess A. Nutrition education in public health programs—What have we learned? Am J Public Health. 1961;51(11):1715–26. doi: 10.2105/ajph.51.11.1715.
24. Baute V, Sampath-Kumar R, Nelson S, Basil B. Nutrition education for the health-care provider improves patient outcomes. Glob Adv Health Med. 2018;7:216495611879599.
25. Schultz JA. Community-based nutrition education: Documentation and evaluation of effectiveness [Master's Thesis]. Iowa State University; 2015. Available from: https://dr.lib.iastate.edu/handle/20.500.12876/28900/
26. Oldewage-Theron WH, Egal A. Impact of nutrition education on nutrition knowledge of public school educators in South Africa: A pilot study. Health SA Gesondheid. 2012;17(1). [Internet]. [cited 2024 Mar 17]. Available from: http://www.scielo.org.za/scielo.php?script=sci_arttext&pid=S2071-97362012000100011&lng=en&nrm=iso

27. Rustad C, Smith C. Nutrition knowledge and associated behaviour changes in a holistic, short-term nutrition education intervention with low-income women. J Nutr Educ Behav. 2013;45(6):490–8. doi: 10.1016/j.jneb.2013.06.009.
28. Walsh CM, Dannhauser A, Joubert G. Impact of a nutrition education programme on nutrition knowledge and dietary practices of lower socioeconomic communities in the Free State and Northern Cape. S Afr J Clin Nutr. 2003;16(3):89–95. [Internet]. [cited 2024 Mar 17]. Available from: https://www.semanticscholar.org/paper/Impact-of-a-nutrition-education-programme-on-and-of-Walsh-Dannhauser/33731bd4dc0e3c542fbd87cf837f380cc802eada
29. Whitehead D, Wang Y, Wang J, Zhang J, Sun Z, Xie C. Health promotion and health education practice: Nurses' perceptions. J Adv Nurs. 2008;61(2):181–7. doi: 10.1111/j.1365-2648.2007.04479.x.
30. Martin L, Leveritt MD, Desbrow B, Ball LE. The self-perceived knowledge, skills and attitudes of Australian practice nurses in providing nutrition care to patients with chronic disease. Fam Pract. 2014;31(2):201-8. doi: 10.1093/fampra/cmt070.
31. Volek JS, Feinman RD. Carbohydrate restriction improves the features of Metabolic Syndrome. Nutr Metab. 2005;2:1–17. doi: 10.1186/1743-7075-2-31.
32. Vorster HH, Badham JB, Venter CS. An introduction to the revised South African food-based dietary guidelines. S Afr J Clin Nutr. 2013;26(sup1):S5-S12.
33. Feinman RD, Vernon MC, Westman EC. Low carbohydrate diets in family practice: What can we learn from an internet-based support group. Nutr J. 2006;5(1). doi: 10.1186/1475-2891-5-26.
34. Sboros S. The Noakes Trial, two years later. 2020. [Internet]. [cited 2024 Mar 17]. Available from: https://www.nmsociety.org/the-scene/noakestrial.

35. Murdoch C, Unwin D, Cavan D, Cucuzzella M, Patel M. Adapting diabetes medication for low carbohydrate management of type 2 diabetes: A practical guide. Br J Gen Pract. 2019;69(684):360-1. doi: 10.3399/bjgp19X704525.
36. Mozaffarian D. Dietary and policy priorities for cardiovascular disease, diabetes, and obesity. Circulation. 2016;133(2):187–225. doi: 10.1161/CIRCULATIONAHA.115.018585.
37. Banting WB. Letter on corpulence, addressed to the public: Third Edition. Obes. Res. 1987. [Internet]. [cited 2024 Mar 17]. Available from: https://onlinelibrary.wiley.com/doi/pdf/10.1002/j.1550-8528.1993.tb00605.x
38. Pujol-Busquets G, Smith J, Larmuth K, et al. Exploring the perceptions of women from under-resourced South African communities about participating in a low-carbohydrate high-fat nutrition and health education program: A qualitative focus group study. Nutrients. 2020;12(4):894. doi: 10.3390/nu12040894.
39. Pujol-Busquets G, Smith J, Fàbregues S, et al. Community assessment for a low-carbohydrate nutrition education program in South Africa. Nutrients. 2023;15(1):67. doi: 10.3390/nu15010067.
40. Pujol-Busquets G, Smith J, Fàbregues S, Bach-Faig A, Larmuth K. Mixed methods evaluation of a low-carbohydrate high-fat nutrition education program for women from underserved communities in South Africa. Appetite. 2025;204:107725. doi: 10.1016/j.appet.2024.107725.
41. Johnston BC, Kanters S, Bandayrel K, et al. Comparison of weight loss among named diet programs in overweight and obese adults: A meta-analysis. JAMA. 2014;312(9):923-33. doi: 10.1001/jama.2014.10397.

42. Franz MJ, VanWormer JJ, Crain AL, et al. Weight-loss outcomes: A systematic review and meta-analysis of weight-loss clinical trials with a minimum 1-year follow-up. J Am Diet Assoc. 2007;107(10):1755-67. doi: 10.1016/j.jada.2007.07.017.

43. Brink JG, Hassoulas J. The first human heart transplant and further advances in cardiac transplantation at Groote Schuur Hospital and the University of Cape Town. Cardiovasc J Afr. 2009;20(1):31-5. PMID: 19287813; PMCID: PMC4200566.

44. Adler P, Adler P. How many qualitative interviews is enough? Natl Cent Res Methods Rev Pap. 2012;1–42. [Internet]. [cited 2024 Mar 17]. Available from: http://eprints.ncrm.ac.uk/2273/.

~~44.~~45. Braun V, Clarke V. Thematic analysis: A practical guide. London: SAGE Publications; 2022.

~~45. Dworkin SL. Sample Size Policy for Qualitative Studies Using In-Depth Interviews. Arch Sex Behav. 2012;41(6):1319–20. doi: 10.1007/s10508-012-0016-6.~~

46. Braun V, Clarke V. Using thematic analysis in psychology. Qual Res Psychol. 2006;3(2):77–101. doi: 10.1191/1478088706qp063oa.

47. Bloor M. Techniques of validation in qualitative research: a critical commentary. In: Miller G, Dingwall R, editors. Context and method in qualitative research. London: SAGE Publications; 1997. p. 37–50.

~~46.~~48. Tong A, Sainsbury P, Craig J. Consolidated criteria for reporting qualitative research (COREQ): a 32-item checklist for interviews and focus groups. Int J Qual Health Care. 2007;19(6):349-357. doi:10.1093/intqhc/mzm042.

~~47. Alaba O, Chola L. The social determinants of multimorbidity in South Africa. Int J Equity Health. 2013;12(1):1–10. doi: 10.1186/1475-9276-12-63.~~

~~48. Seekings J, Nattrass N. Class, Race, and Inequality in South Africa. Foreign Affairs. 2006;85(3):173. doi: 10.2307/20032025.~~

Formatted: Font: (Default) Times New Roman

Formatted: Not Highlight

Formatted: Font: (Default) Times New Roman, Not Highlight

Formatted: Font: (Default) Times New Roman, Not Italic, Not Highlight

Formatted: Font: (Default) Times New Roman, Not Highlight

Formatted: Font: (Default) Times New Roman

Formatted: Not Highlight

Formatted: Font: (Default) Times New Roman

Formatted: Font: (Default) Times New Roman, Not Italic

Formatted: Font: (Default) Times New Roman

Formatted: Font: (Default) Times New Roman, Not Highlight

Formatted: Not Highlight

Formatted: Font: Not Italic, Not Highlight

Formatted: Not Highlight

Formatted: Pattern: Clear (Yellow)

Formatted: Font: (Default) Times New Roman

~~49. Puoane T, Fourie JM, Shapiro M, Rosling L, Tshaka NC, Oelefse A. "Big is beautiful" – an exploration with urban black community health workers in a South African township. S Afr J Clin Nutr. 2005;18(1):6-15. doi: 10.1080/16070658.2005.11734033.~~

~~50. Ndinda C, Uzodike UO, Chimbwete C, Pool R. Gender relations in the context of HIV/AIDS in rural South Africa. AIDS Care. 2007;19(7):844-9. doi: 10.1080/09540120701203923.~~

~~51. Vorster HH, Kruger A, Margetts BM. The nutrition transition in Africa: Can it be steered into a more positive direction? Nutrients. 2011;3(4):429-41. doi: 10.3390/nu3040429.~~

~~52.~~49. Steyn N, Ochse R. Food-Based Dietary Guidelines for South Africa: "Enjoy a variety of foods": As a food-based dietary guideline. S Afr J Nutr. 2013;26(3):13–7. (Supplement):S1-S164. [Internet]. [cited 2024 Mar 17]. Available from: https://openknowledge.fao.org/server/api/core/bitstreams/2cf12134-bb2d-47b1-92b0-403b028c60ad/content

~~53.~~50. Hunter-Adams J, Battersby J. Health care providers' perspectives of diet-related non-communicable disease in South Africa. BMC Public Health. 2020;20(1). doi: 10.1186/s12889-020-8364-y.

~~54.~~51. McKinlay E, Plumridge L, McBain L, McLeod D, Pullon S, Brown S. "What sort of health promotion are you talking about?": A discourse analysis of the talk of general practitioners. Soc Sci Med. 2005;60(5):1099–106. doi: 10.1016/j.socscimed.2004.06.041.

~~55.~~52. Smolowitz J, Speakman E, Wojnar D, Whelan EM, Ulrich S, Hayes C, et al. Role of the registered nurse in primary health care: Meeting health care needs in the 21st century. Nurs Outlook. 2015;63(2):130–6. doi: 10.1016/j.outlook.2014.08.004.

~~56.~~53. Barton AJ, Skiba DJ, Sorensen L. Preparing the next generation of advanced practice nurses. Stud Health Technol Inform. 2004;107(Pt 2):860-4. PMID: 15360934.

Formatted: Font: (Default) Times New Roman

~~57.~~54. Xu X, Parker D, Ferguson C, Hickman L. Where is the nurse in nutritional care? Contemp Nurse. 2017;53(3):267-70. doi: 10.1080/10376178.2017.1370782.

~~58.~~55. Jansink R, Braspenning J, van der Weijden T, Elwyn G, Grol R. Primary care nurses struggle with lifestyle counseling in diabetes care: A qualitative analysis. BMC Fam Pract. 2010;11. doi: 10.1186/1471-2296-11-41.

~~59.~~56. Marshall AP, Takefala T, Williams LT, Spencer A, Grealish L, Roberts S. Health professional practices and their influence on nutritional intake of hospitalised patients. Int J Nurs Sci. 2019;6(2):162–8. doi: 10.1016/j.ijnss.2019.03.008.

~~60.~~57. Holmberg C. Politicization of the Low-Carb High-Fat diet in Sweden, promoted on social media by non-conventional experts. Int J E-Polit. 2015;6(3):27–42. doi: 10.4018/ijep.2015070103.

~~61.~~58. Guidelines Advisory Committee D. Scientific Report of the Dietary Guidelines Advisory Committee Advisory Report to the Secretary of Agriculture and Secretary of Health and Human Services. 2020. [Internet]. [cited 2024 Mar 17]. Available from: https://www.dietaryguidelines.gov/2020-advisory-committee-report.

~~62.~~59. ~~McPhee J. Exploring the Acceptability of, and Adherence to, a Carbohydrate-Restricted, Higher Fat Diet as an Instrument for Weight Loss in Women Aged 40–55 Years [Master's Thesis]. Auckland University of Technology; 2018. [Internet]. [cited 2024 Mar 17]. Available from: https://researchspace.auckland.ac.nz/docs/uoa-docs/rights.htm~~Pilnick A, Hindmarsh J, Gill VT, editors. Communication in healthcare settings: policy, participation and new technologies. Chichester: Wiley-Blackwell; 2010.

~~63.~~60. Webster CC, Murphy TE, Larmuth KM, Noakes TD, Smith JA. Diet, diabetes status, and personal experiences of individuals with type 2 diabetes who self-selected and followed a Low Carbohydrate High Fat diet. Diabetes Metab Syndr Obes. 2019;12:2567–82. doi:10.2147/DMSO.S227090.

Formatted: Font: Not Italic

~~64.~~61. Jallinoja P, Niva M, Helakorpi S, Kahma N. Food choices, perceptions of healthiness, and eating motives of self-identified followers of a low-carbohydrate diet. Food Nutr Res. 2014;58:1–9. doi: 10.3402/fnr.v58.23552.

~~65.~~62. Clarke C, Best T. Low-carbohydrate, high-fat dieters: Characteristic food choice motivations, health perceptions and behaviours. Food Qual Prefer. 2017;62:162–71. doi: 10.1016/j.appet.2019.104324.

~~66.~~63. Arthur TH. Perceptions and acceptability of Low Carbohydrate, High Fat (LCHF) diets among Māori whānau in Dunedin. [Master's Thesis]. University of Otago; 2018. [Internet]. [cited 2024 Mar 17]. Available from: https://ourarchive.otago.ac.nz/esploro/

~~67.~~64. Aubel J. The role and influence of grandmothers on child nutrition: Culturally designated advisors and caregivers. Matern Child Nutr. 2012;8(1):19–35. doi: 10.1111/j.1740-8709.2011.00333.x.

~~68.~~65. Cassel J. Social and cultural implications of food and food habits. Am J Public Health. 1957;47:732–40. doi: 10.2105/ajph.47.6.732.

66. Hartanto A, Lau IYM, Yong JC. Culture moderates the link between perceived obligation and biological health risk: Evidence of culturally distinct pathways for positive health outcomes. Soc Sci Med. 2020;244. doi: 10.1016/j.socscimed.2019.112644.

67. Puoane T, Fourie JM, Shapiro M, Rosling L, Tshaka NC, Oelefse A. "Big is beautiful" - an exploration with urban black community health workers in a South African township. S Afr J Clin Nutr. 2005;18(1):6-15. doi: 10.1080/16070658.2005.11734033.

68. Ndinda C, Uzodike UO, Chimbwete C, Pool R. Gender relations in the context of HIV/AIDS in rural South Africa. AIDS Care. 2007;19(7):844-9. doi: 10.1080/09540120701203923.

69. Vorster HH, Kruger A, Margetts BM. The nutrition transition in Africa: Can it be steered into a more positive direction? Nutrients. 2011;3(4):429-41. doi: 10.3390/nu3040429.

~~69.~~70. Seekings J, Nattrass N. Class, race, and inequality in South Africa. Foreign Affairs. 2006;85(3):173. doi: 10.2307/20032025.

~~70.~~71. Rankin D, Backett-Milburn K, Platt S. Practitioner perspectives on tackling health inequalities: Findings from an evaluation of healthy living centres in Scotland. Soc Sci Med. 2009;68(5):925–32. doi: 10.1016/j.socscimed.2008.12.013.

~~71.~~72. Strang AB, Ager A. Psychosocial interventions: Some key issues facing practitioners. Intervention. 2003;1(3):2–12. [Internet]. [cited 2024 Mar 17]. Available from: https://citeseerx.ist.psu.edu/document?repid=rep1&type=pdf&doi=bd2177acc71bac53c13b6735ae7c918f85283aa8

~~72.~~73. Kok MO, Vaandrager L, Bal R, Schuit J. Practitioner opinions on health promotion interventions that work: Opening the “black box” of a linear evidence-based approach. Soc Sci Med. 2012;74(5):715–23. doi: 10.1016/j.socscimed.2011.11.021.

Formatted: Font: (Default) Times New Roman

Response to Reviewers' comments

Manuscript Title: *Between support and scepticism: Health professionals' views on Low-Carbohydrate diets in under-resourced South African communities*.

New Tile: *Between support and scepticism: Health professionals' perceptions on a nutrition education program promoting Low-Carbohydrate, High-fat diets in under-resourced South African communities.*

Manuscript ID: PONE-D-24-55230

Dear Editor and Esteemed Reviewers,

On behalf of all co-authors, I would like to sincerely thank the reviewers for their thoughtful and constructive feedback on our manuscript. We greatly appreciate the time and expertise that went into reviewing our work, and we are confident that the suggested revisions have significantly improved the quality and clarity of our submission. We have carefully considered each comment and have responded in detail below, outlining the changes made to the manuscript and providing clarifications where necessary.

We trust that our revisions have addressed the reviewers' concerns, and we look forward to your further consideration of our manuscript for publication in *PLOS ONE*.

Sincerely,

Georgina Pujol-Busquets

Reviewers' comments:

Comments to the Author

1. Is the manuscript technically sound, and do the data support the conclusions?

The manuscript must describe a technically sound piece of scientific research with data that supports the conclusions. Experiments must have been conducted rigorously, with appropriate controls, replication, and sample sizes. The conclusions must be drawn appropriately based on the data presented.

Reviewer #1: Yes

Reviewer #2: No

Reviewer #3: Yes

Reviewer #4: Partly

2. Has the statistical analysis been performed appropriately and rigorously?

Reviewer #1: N/A

Reviewer #2: No

Reviewer #3: Yes

Reviewer #4: N/A

3. Have the authors made all data underlying the findings in their manuscript fully available?

The PLOS Data policy<http://track.editorialmanager.com/CL0/http:%2F%2Fwww.plosone.org%2Fstatic%2Fpolicies.action%23sharing/1/010f019547d21ab6-08e7bc50-c91e-4e43-a149-14affdf0e8f0-000000/uoBneyU2ESJ2opnWfelmiRHTzi2lhcmhawIbFSjISQo=200> requires authors to make all data underlying the findings described in their manuscript fully available without restriction, with rare exception (please refer to the Data Availability Statement in the manuscript PDF file). The data should be provided as part of the manuscript or its supporting information, or deposited to a public repository. For example, in addition to summary statistics, the data points behind means, medians and variance measures should be available. If there are restrictions on publicly sharing data—e.g. participant privacy or use of data from a third party—those must be specified.

Reviewer #1: No

Reviewer #2: No

Reviewer #3: No

Reviewer #4: No

4. Is the manuscript presented in an intelligible fashion and written in standard English?

PLOS ONE does not copyedit accepted manuscripts, so the language in submitted articles must be clear, correct, and unambiguous. Any typographical or grammatical errors should be corrected at revision, so please note any specific errors here.

Reviewer #1: Yes

Reviewer #2: Yes

Reviewer #3: Yes

Reviewer #4: Yes

5. Review Comments to the Author

Please use the space provided to explain your answers to the questions above. You may also include additional comments for the author, including concerns about dual publication, research ethics, or publication ethics. (Please upload your review as an attachment if it exceeds 20,000 characters)

<u>Responses to Reviewers' Questions</u>

Reviewer #1: This study explores healthcare professionals' perspectives on nutrition and the EBSA program, drawing on 16 in-depth individual interviews with physicians, nurses, and dietitians from False Bay Hospital, Groote Schuur Hospital, and a primary health clinic in Hout Bay, Western Cape, South Africa. Thematic analysis revealed four main theme- Foundations of dietary understanding, LCHF: Controversies and beliefs; EBSA's role in community nutrition; and understanding socioeconomic determinants of health. It is nicely written, and I have no major concerns. I offer the following suggestions for your consideration:

1. While I absolutely agree with the first paragraph, I felt that it didn't align well with the study performed. Can this be more focussed on the research question? Or, perhaps, just delete it and start with the sentence in line 70 ("Despite nutrition's…")

Response:

We agree that the first paragraph did not fully align well with the study focus. As suggested, the paragraph has been modified, and the Introduction now begins with:
"South Africa's public health system faces major challenges, including long wait times, resource shortages, poor management, inadequate infection control, and workforce deficits—exacerbated by inequitable resource distribution, rapid urbanization, and the dual burden of infectious and non-communicable diseases (NCDs) [1–9]."

2. What would be useful to a reader not intimately familiar with SA nutrition/health systems would be a brief (just brief, no need for >4 sentences) comparison of EBSA vs. standard dietary guidance in South Africa. Could this be added to provide this context?

Response:

We have added a brief comparison between EBSA and standard dietary guidance in South Africa in the Introduction. This contextual framing is now included to orient readers unfamiliar with South African dietary policy.

"EBSA promotes a low-carbohydrate high-fat (LCHF) dietary approach, with an emphasis on nutrient-dense, minimally processed foods [31]. The LCHF approach contrasts with the South

African Food-Based Dietary Guidelines, which emphasise balanced diets with staple whole grains, moderate fat intake, and a heavy focus on plant-based foods [32].”

Reference:
32. Vorster HH, Badham JB, Venter CS. An introduction to the revised South African food-based dietary guidelines. S Afr J Clin Nutr. 2013;26(Suppl 1):S5–S12.

3. Was only selecting English speaking participants a major bias?

Response:
We acknowledge this as a limitation and have now explicitly mentioned this in the limitations section at the of the Discussion, noting that the exclusion of non-English speakers may have limited the diversity of perspectives captured.

“The diverse perspectives of physicians, nurses, and dietitians offer a comprehensive view, but the uneven sample size, limited facility access, the affiliation of one dietitian with an NGO, and the exclusion of non-English speakers may have limited the diversity of perspectives captured and introduced biases. Future studies should include non-English-speaking health professionals to capture broader cultural perspectives.”

4. Why is “original language” stated in line 153? Were all interviews not conducted in English, as stated in line 137-38?

Response:
This has been corrected to explicitly state that all interviews were conducted in English. The term “original language” has been removed to avoid confusion.

5. Was member checking performed? If not, might be good to justify why it was not done.

Response:
Member checking was not performed in this study due to both logistical and methodological considerations. Practically, the time and resource constraints inherent in the research timeline made it infeasible to re-engage participants for feedback. However, beyond these constraints, there are also epistemological and methodological concerns associated with the use of member checking in qualitative research of this nature. One common critique, as discussed by Bloor (1997), is that member validation exercises are not neutral or definitive tests of

validity, but rather complex social encounters influenced by context, participant subjectivity, and the changing nature of perception. As Bloor demonstrates, members' reactions to findings are often shaped by the passage of time, evolving perspectives, and the dynamics of the feedback process itself. Participants might reject or revise earlier statements not because the initial data were invalid, but because their current viewpoints differ, highlighting the inherently dynamic and situated nature of perceptions. Furthermore, member checking has been critiqued for its roots in positivist assumptions about data validation—that there is a singular, fixed "truth" that participants can confirm. In interpretive qualitative paradigms, such as the one guiding this study, the researcher acknowledges that data are co-constructed and context-dependent, and that seeking verification from participants can sometimes undermine the complexity and multiplicity of meanings present in qualitative data (Bloor, 1997).

Therefore, the decision not to employ member checking aligns with both practical limitations and a reflexive approach that prioritizes depth of interpretation over post hoc validation by participants.

"We did not use member checking for practical and methodological considerations. Logistically, time and resource constraints prevented following up with participants. Methodologically, we adopted a reflexive approach that recognises the dynamic and contextual nature of participants' perceptions, which may shift over time and are not necessarily fixed or singular. Participant feedback is shaped by the circumstances in which it is elicited and does not constitute a definitive test of validity [47]."

Reference:

47. Bloor M. Techniques of validation in qualitative research: a critical commentary. In: Miller G, Dingwall R, editors. *Context and method in qualitative research*. London: SAGE Publications; 1997. p. 37–50.

6. Which profession usually counsels on nutrition in this setting? If it's dietitians, would having only 2/16 participants as dietitians be a limitation of perspective?

Response:

We have clarified in the limitations section in the Discussion that while dietitians are typically the lead providers of dietary counselling, nurses and physicians in under-resourced

primary care settings often deliver nutrition advice due to staff shortages. This sampling choice, therefore, reflects real-world task-shifting common in South African healthcare.

“Additionally, while dietitians are typically the primary providers of dietary counselling, in under-resourced primary care settings, nurses and physicians often take on this role due to staff shortages [52]. This reflects the real-world task-shifting that is common in South African healthcare, where non-specialist providers must frequently step in to deliver nutrition advice. While dietitians play a central role in dietary counselling, this study included physicians and nurses to capture how nutrition messages are communicated in primary care, aligning with community health models for under-resourced settings. Future research focusing solely on dietitians could provide more specialized insights into LCHF counselling and implementation.”

7. Discussion line 448- could you provide an example of what is meant by “backhanded support”?

Response:

This phrase has been clarified in the Discussion to mean that professionals reluctantly supported patients who chose LCHF diets, not because they endorsed the diet themselves, but because they wanted to encourage patient autonomy. This illustrates a phenomenon known as therapeutic pragmatism, where professionals adopt a supportive stance despite personal doubts.

“Their reluctance stemmed from concerns about the diet’s alignment with conventional dietary guidelines, a lack of confidence in providing LCHF-specific advice, and uncertainty about its long-term health impacts. Rather than actively endorsing LCHF diets, professionals supported patients out of a desire to respect patient autonomy, a stance known as therapeutic pragmatism, where healthcare providers adopt a supportive role despite personal doubts [59]. This meant that while they outwardly agreed to assist patients following an LCHF approach, their hesitance and underlying scepticism often resulted in a lack of meaningful guidance, leaving patients to navigate dietary changes largely on their own.”

Reference:

59. Pilnick A, Hindmarsh J, Gill VT, editors. Communication in healthcare settings: policy, participation and new technologies. Chichester: Wiley-Blackwell; 2010. 160 p. ISBN: 978-1-4051-9827-1.

8. Discussion line 448-450- this is an interesting and possibly loaded interpretation. Could you expand? Is it that the doctors believed in LCHF (despite admitting limited nutrition knowledge), and were reluctant ("sometimes") to send to dietitians because they feared that dietitians would not provide LCHF advice?

Response:

This section has been expanded at the end of the Discussion to explain that some doctors privately believed in LCHF's potential benefits (often informed by media rather than training), but were reluctant to refer to dietitians because they expected dietitians to discourage LCHF in favor of conventional low-fat advice.

"Despite these challenges, the study offers valuable insights into health professionals' attitudes towards nutritional advice and LCHF diets, contributing to discussions on community healthcare and NCD management. Notably, some doctors privately believed in LCHF's potential benefits—often informed more by media than formal training—but were reluctant to refer patients to dietitians, anticipating that dietitians would discourage LCHF in favor of conventional low-fat advice [13]."

9. Discussion lines 459-464; could you clarify- was the failure to endorse LCHF in communities one of the group, in that the support group itself (was this composed of patients) was not endorsing LCHF? It's not clear

Response:

This has been revised to clarify in the Discussion that some professionals felt community-based support groups themselves were hesitant to fully endorse LCHF diets. This stemmed from uncertainty, conflicting advice from different health workers, and broader scepticism about non-traditional diets within these groups.

"Many healthcare professionals remained cautious about formally supporting the diet, reflecting a broader global trend where media-driven interest does not always translate into clinical adoption [60]. This suggests that scepticism about LCHF is not solely a matter of questioning its health implications but rather an uncertainty about how it can be practically adopted in resource-limited settings. Some professionals also noted that hesitation toward LCHF did not come solely from healthcare providers but was also present within the support groups themselves, which were composed of community members, including patients. This

reluctance within the groups stemmed from conflicting advice received from different health workers, scepticism about non-traditional diets, and broader uncertainty about the feasibility of LCHF within low-income settings [61-63]. While some professionals acknowledged that the program might be effective in changing perceptions about diet, concerns about the LCHF approach were also raised."

Reviewer #2:

1. May please add some of the observations in the tabulated form.

Response:

Thank you for your suggestion. Table 2 (now Table 1) was developed initially to provide deeper insights into the themes, offering a structured view of health professionals' perspectives with definitions and direct quotes to support the qualitative analysis. By summarizing key findings while maintaining participant perspectives, we think that Table 1 effectively presents observations in a clear, accessible format. We believe it meets the request but welcome further clarification if needed.

2. It would be better if you could convert the qualitative discussion output as quantitative values, which can be statistically analyzed.

Response:

Thank you for the comment. While some qualitative studies may include elements of quantification, we do not consider it appropriate in the context of this particular study. The approach employed is interpretive, where the aim is to explore meaning and complexity rather than to measure frequency.

3. Sentences may be edited for clarity.

Response:

The manuscript has undergone thorough language editing to improve clarity and readability.

4. The presented conclusion could be part of the discussion, and a new conclusion based on the study conducted may be developed.

Response:

Thank you for your comment. The Conclusion has been rewritten to focus explicitly on the study’s contributions, see the revised section:

“This study provides valuable insights into healthcare professionals’ perspectives on a nutrition education program recommending LCHF diets, highlighting key challenges in their understanding and implementation within under-resourced South African communities. Professionals expressed uncertainty around LCHF, often shaped by conflicting guidelines, limited exposure to recent evidence, and scepticism about its long-term effects. The findings also reveal significant training gaps in nutrition education, with many professionals, mostly doctors and nurses, lacking confidence in providing dietary guidance beyond conventional recommendations. Additionally, socioeconomic barriers such as food affordability, cultural norms, and structural inequalities further complicate dietary behaviour change in these communities. Despite these challenges, according to participants, EBSA presents a promising model for integrating nutrition education into healthcare strategies, particularly by offering structured support and practical guidance tailored to local contexts. Future efforts should focus on bridging knowledge gaps, strengthening professional training, and ensuring that dietary interventions are both evidence-based and accessible to those who need them most.”

5. Was there any control group?

Response:
As an exploratory qualitative study, no control group was used, as this approach aligns with the principles of qualitative research, which prioritise an in-depth understanding of the phenomena under investigation over comparative analysis (Yin 2011).

Reference:
Yin, R. K. (2011). *Qualitative research from start to finish.* Guilford Press.

Reviewer #3: Review comments
Title: Between support and scepticism :Health professional's views on low carbohydrate diest in under-resourced South African communities.
The paper is written well except the following gaps;
1.Content or focus: much of professional/expert skepticism is raised against the applicability or practicability of EBSA's LCHF program ,not on its health implications. All the issues under theme 4 are arguments questioning practicability.Thus, it would have been better the research was framed along that direction.

Response:

We appreciate the reviewer's insightful feedback and agree that much of the skepticism raised by professionals focused on the practical applicability of EBSA's LCHF program rather than purely on concerns about health implications. However, we believe that both aspects—uncertainty about the diet's feasibility in under-resourced settings and concerns about its long-term health effects—were present in the data. To align with the reviewer's suggestion, we have revised the Discussion section to ensure that implementation challenges—such as affordability, accessibility, and food security—are clearly emphasized as primary concerns among professionals. We have clarified that practical barriers, rather than outright rejection of LCHF as a nutritional intervention, were central to skepticism. However, we have also retained references to health-related concerns, as some professionals questioned the sustainability and long-term impact of LCHF on metabolic health, particularly in patients with comorbidities. We appreciate this valuable feedback and believe these refinements strengthen the clarity of our findings. We hope this revision adequately addresses the concern, but we welcome any further suggestions for improvement. Please see the track changes version of the manuscript to see all the changes in the Discussion.

2.Your findings go beyound the scope of your title. Your title is restricted only to Low Carbohydrate Diets, but your findings and discussions are focusing more on the controvercies over High Fat Diets. Look at the folowing on the sources of skepticism; line 35, 303, 311-312, and 420. These all are stressing on expert caution on High Fat diets than Low Carbohydrates.

Response:

We appreciate this observation and have revised the title to better reflect the scope of the study, incorporating both *low carbohydrate* and *high fat* aspects to align with the term "LCHF" (Low-Carbohydrate, High-Fat). This dual emphasis is critical as it captures the full nature of the dietary recommendations under review, consistent with international definitions of LCHF diets. Please see the Title Page document for the revised version.

Revised Title: "*Between support and scepticism: Health professionals' perceptions on a nutrition education program promoting Low-Carbohydrate, High-fat diets in under-resourced South African communities.*"

3.Unsupported and contradictory statements. For example,

A)health professional opinions on the role of nutritional education, (line 2002-2003 contradicts with (line 421_422).

Response:

Thank you for pointing out this inconsistency regarding health professionals' views on nutritional education. We recognize that the statements in line 202-203 and line 421-422 could be interpreted as contradictory. To clarify, professionals acknowledged the importance of nutritional education in disease prevention but simultaneously expressed challenges in integrating it into their routine practice due to time constraints and limited expertise. Their recognition of its importance does not necessarily translate into their ability or confidence to provide such education effectively.

To resolve this contradiction, we have revised the Discussion.

"While physicians and nurses recognized the value of nutritional education in disease prevention, their ability to provide effective dietary counselling was hindered by systemic barriers, including time constraints, competing clinical responsibilities, and inconsistent nutritional messaging. [50]."

B)information access of professionals,(line 229 contradicts with line 430).

Response:

We appreciate the reviewer's observation regarding the apparent contradiction in how professionals discuss access to nutritional information. We acknowledge that while professionals reported struggles in keeping up with evolving nutrition science, they simultaneously relied on established dietary concepts that were not always critically examined. To address this, we have revised the Discussion section to clarify that health professionals acknowledged the need for more clinical trials to strengthen evidence-based dietary guidelines but continued to use established dietary terminology without necessarily scrutinizing its scientific basis. This revision ensures that both aspects, their difficulty in staying updated with nutrition science and their reliance on conventional dietary messaging, are accurately represented. To resolve this contradiction, we have revised the Discussion.
"While health professionals expressed difficulty in staying updated with advancements in nutrition science, they also relied heavily on widely accepted dietary principles without necessarily questioning or critically analyzing them. Furthermore, many professionals agreed that more clinical trials were needed to reassess current dietary guidelines, reflecting an

awareness of the evolving nature of nutrition science. This duality, acknowledging the need for stronger evidence while continuing to use conventional dietary rhetoric, highlights the complexity of how professionals navigate nutritional knowledge in practice."

C)popularity of LCHF diets in south africa (504) contradicts with lack of support and stopping to follow those diets mentioned in the manuscript

Response:

We appreciate the reviewer's observation regarding the apparent contradiction between the reported popularity of LCHF diets in South Africa and the lack of professional support for and continued adherence to these diets. To clarify, we have revised the paragraph to distinguish between public/media-driven popularity and actual adoption by healthcare professionals and their patients. This revision ensures that the distinction between public enthusiasm and professional skepticism is made explicit, addressing the concern raised by the reviewer.

"While LCHF diets are widely recognized in South Africa, their popularity is largely driven by media visibility and public discourse rather than strong institutional or professional endorsement. Many healthcare professionals remained cautious about formally supporting the diet, reflecting a broader global trend where media-driven interest does not always translate into clinical adoption [60]. This suggests that scepticism about LCHF is not solely a matter of questioning its health implications but rather an uncertainty about how it can be practically adopted in resource-limited settings. Some professionals also noted that hesitation toward LCHF did not come solely from healthcare providers but was also present within the support groups themselves, which were composed of community members, including patients. This reluctance within the groups stemmed from conflicting advice received from different health workers, scepticism about non-traditional diets, and broader uncertainty about the feasibility of LCHF within low-income settings [61-63]."

D.effectiveness of LCHF, consensus guidelines on LCHF (line 74)Vs lack of scientific consensu on it (309)

Response:

We appreciate the reviewer's observation regarding the apparent contradiction between the existence of international consensus guidelines on LCHF (line 74) and the mention of a lack

of scientific consensus on it (line 309). To clarify, we have revised the paragraph to distinguish between the growing international support for carbohydrate restriction in diabetes management and the remaining concerns among healthcare professionals about its long-term effects, feasibility, and sustainability. This revision ensures that both the established guidelines and the persisting concerns among practitioners are accurately represented.

"Their primarily concern centred around the high fat content of LCHF diets and whether the diet was financially sustainable for patients from low-income communities. Many professionals believed that LCHF foods were expensive, making it difficult for patients to maintain the diet long-term. Some were willing to support patients who chose to follow LCHF, but their support was often condition on closely monitoring patients for potential negative effects. While international consensus guidelines recognize carbohydrate restriction as an effective approach for managing type 2 diabetes, scepticism remains among some health professionals, particularly regarding its long-term sustainability and applicability in under-resourced settings. Some health professionals questioned whether LCHF could effectively treat metabolic disorders, largely due to ongoing controversy surrounding the diet and the lack of full scientific agreement on its long-term effects."

E. Venue of meeting interviewee: private venue(line 137) Vs in the health facilities(519)

F.Duration of contact with interviewees: one hour(137) Vs thirty minutes(141)

Response:

This has been corrected for consistency. Most interviews were conducted at private venues within the participating health facilities, ensuring both privacy and accessibility and they were scheduled for 30-60 minutes allowing for flexibility depending on participant availability and depth of discussion. Please see the Participants section for the revision.

"The participants met individually with a researcher for approximately thirty minutes to an hour in a private venue within the participating health facilities, ensuring both privacy and accessibility, where they signed the informed consent form and were interviewed in English."

G.selection of interviwee was done from facilities that run EBSA program nutrition education (113). That means they are expected to be knowledgabe about the program.However, you have written (line 329-330) that "majority were introduced to (EBSA) program for the first time during the interview"

Response:

We clarified that although participants were drawn from facilities serving communities where EBSA had run programs, this did not guarantee their direct involvement in or awareness of the program itself. This is now explained explicitly in the Participant section.

“Although participants were selected from some facilities serving communities where EBSA had run programs, this did not guarantee their direct involvement in or awareness of the program itself. Additionally, including professionals from communities where EBSA was planning future programs allowed the study to capture prospective opinions on the program, offering insight into potential facilitators and barriers before implementation.”

4) lack of clarity on the role of science: line 309 and 221 which similarly state that "confusing information comes from science". These two contradict with line 226 "few health professjonals made any reference to science". Does science resolve doubts or produces and ignites them? It also begs the question , if most of the experts are not refering science, why and how do they can be confused with scientific evidences on the topic?

Response:

We appreciate the reviewer’s insightful observation regarding the apparent contradiction between the claim that science causes confusion (lines 309 and 221) and the statement that few professionals referenced science (line 226). To clarify, we have revised the paragraph to reflect that while science is often perceived as a source of contradictory information, most professionals were not directly engaging with primary scientific literature. Instead, their confusion appears to stem from institutional dietary guidelines, media narratives, and public health messaging rather than direct interpretation of research.

“Nurses and physicians often lacked confidence in their own nutritional knowledge and reported feeling uncertain when discussing dietary concepts. Rather than advising patients themselves, many preferred to refer them to dietitian, especially if they felt the patient required specific nutritional advice. They cited contradictory information from science, media, and government policies as key sources of confusion. Health professionals highlighted this inconsistency as a challenge for both themselves and their patients, contributing to public uncertainty about nutrition. A few health professionals attributed their lack of confidence in providing nutrition advice to insufficient training in both their formal education and continued professional development. Few participants referenced scientific evidence when

justifying dietary recommendations. Therefore, while science was often cited as a source of conflicting information, it was rarely directly referenced by participants. Their confusion appeared to be from inconsistent public health messaging, institutional guidelines, and media influence rather than direct interaction with scientific literature."

5). Tha dat collecton (for this research) conducted in nov 2019 and the corresponding findings might not be of high relevance to adress the new dietary trends, including dietary transition initiatives.

Response:

We acknowledge the reviewer's concern regarding the relevance of findings given that data collection occurred in 2019-2020. To address this, we have added a statement in the Limitations section clarifying that the findings reflect a specific period and that dietary discourse in South Africa may have evolved, particularly in response to pandemic-related changes in food access and health priorities. This temporal framing ensures that readers interpret the findings within the appropriate context.

"This study has a temporal limitation, as data collection took place between November 2019 and February 2020, meaning the findings capture a specific moment in time. Dietary discourse in South Africa may have evolved since then, particularly due to pandemic-related shifts in food access, nutrition policies, and public health priorities. While the study provides valuable insights into health professionals' perspectives, future research is needed to explore how these views may have changed with emerging dietary trends and transition initiatives."

6).inaccurate targeting of the sample population. The right targets should be nutritionists/dieticians. Not only that, they also should not be from private facilities. Generally, the paper has methodological and content gaps that need to be corrected.

Response:

We acknowledge the reviewer's concern regarding the sample selection and the inclusion of healthcare professionals beyond dietitians. While dietitians are indeed key informants in dietary counselling, our study intentionally included physicians and nurses to capture a broader perspective on how nutrition messages are conveyed within primary care. This approach aligns with community health models, which emphasize the role of generalist providers in under-resourced settings where dietitians are not always available. To address

this, we have clarified this rationale we expanded the limitations section to acknowledge the potential for further specialized insights from a dietitian-focused study. This ensures that readers understand the study’s scope while recognizing opportunities for future research.

“While dietitians play a central role in dietary counselling, this study included physicians and nurses to capture how nutrition messages are communicated in primary care, aligning with community health models for under-resourced settings. Future research focusing solely on dietitians could provide more specialized insights into LCHF counselling and implementation.”

Reviewer #4: I am grateful for the opportunity to review this study, which already has the merit of bringing to light a little-known reality and featuring authors whose backgrounds make the work international.
The study is interesting, but I notice that the interviews were conducted more than 5 years ago, and the bibliography is a lot but very dated. I suggest motivating why it took so long to publish this work and what justifies using interviews from 5 years ago. Health issues and contexts evolve very quickly.
I wonder what is new about a paper that analyses data from 5 years earlier. I would therefore suggest specifying well what this study brings that is new.

Response:
We appreciate the reviewer’s concern regarding the timing of data collection and the relevance of findings given the evolving nature of health issues. The study was conducted in 2019-2020, and while there has been a time gap in publication, the findings remain relevant as they provide critical insights into healthcare professionals' perspectives on dietary counseling and LCHF diets in under-resourced settings—issues that continue to be debated globally. To address this, we have added a justification in the Discussion section noting that while dietary discourse and healthcare priorities may have shifted since data collection, the core challenges identified—such as conflicting dietary guidance, limited nutrition training, and practical barriers to implementing dietary interventions—remain persistent in South Africa’s primary care landscape. Additionally, we have acknowledged this as a study limitation while emphasizing that the findings offer a valuable historical perspective on professional attitudes toward LCHF diets at a critical moment in public health discourse.

Furthermore, to ensure that the study remains current, we have reviewed and updated the bibliography to include more recent references where applicable. We also clarify that this

study brings new contributions by being one of the first to explore how a multidisciplinary group of healthcare providers, beyond just dietitians, perceive LCHF diets and community-based nutrition programs in South Africa. These insights help inform future strategies for improving dietary counseling in primary care settings, particularly in under-resourced communities.

Furthermore, the bibliography should be updated to the last 10 years, preferably 5 years. Braun and Clarke 2006 can also be updated.

Response:

Thank you for your suggestion regarding the bibliography. We have reviewed and updated several references to ensure the inclusion of recent and relevant literature. Regarding Braun and Clarke (2006), we have intentionally retained this citation as it remains the foundational reference for the type of thematic analysis conducted in our study. Although more recent work by the authors exists, including their 2022 publication, the methodological approach we used aligns specifically with the principles outlined in their 2006 paper, rather than with reflexive thematic analysis as defined in later works. We have clarified this in the manuscript to maintain consistency and methodological rigour. However, we have included Braun and Clarke (2022) in the reference list to acknowledge and address the ongoing debate around the concept of data saturation in qualitative research, as discussed in our section on sampling and data sufficiency.

We have added the following statement to clarify our approach:

"The 2006 version of Braun and Clarke's thematic analysis was used. This version is characterised by its epistemological flexibility and openness to the use of codebooks, a feature that has since been abandoned by the authors in more recent iterations of this analytical method [45]."

Here are some more suggestions:

Title: Specify in the title the type of research: a qualitative research

Response:

We appreciate the reviewer's suggestion to specify the research type in the title. However, we believe that the current title, "Between support and skepticism: Health professionals' views on a nutrition education program promoting Low-Carbohydrate, High-fat diets in under-

resourced South African communities" already implies a qualitative approach. The term "views" naturally indicates that the study explores perceptions and experiences, which aligns with qualitative research methodologies. Additionally, the abstract and methods section clearly define the study as a qualitative investigation, including details on in-depth interviews and thematic analysis. Given this, we feel that explicitly stating "qualitative research" in the title is unnecessary. That said, if the editorial team or reviewers strongly prefer including a methodological descriptor, we are open to refining the title to ensure clarity while maintaining its impact.

Keywords: Redefine keywords by adding for example perceptions, diet... and changing the order of the words: 6. thematic analysis; 3 low-carbohydrate, high-fat diet; 5 South Africa; 4 under-resourced communities; 2. health professionals. 1. perspectives on nutrition

Response:

We appreciate the reviewer's suggestion to refine the keywords for better alignment with the study's focus. These adjustments improve clarity by emphasizing perspectives and perceptions, restructuring the order for better readability, and ensuring the keywords accurately reflect the study's scope. We appreciate this valuable suggestion.

Based on this feedback, we have revised the keywords as follows:

Revised Keywords:
perspectives on nutrition; healthcare professionals; low-carbohydrate, high-fat diet (LCHF); under-resourced communities; South Africa; thematic analysis; qualitative research.

Background:

The EBSA programme is not adequately described and the relevant literature does not support the use of this diet with solid evidence. I suggest that it should be better explained and recent and specific literature on this programme should be included. If it is a new programme, it should be explained in detail.

I would also explain in more detail studies 38-39-40 that are already specifically evaluating this programme.

Response:

We have added detail on EBSA's approach and placed it within the context of broader South African dietary guidelines in the Introduction.

"Various nutritional education initiatives are active in low socio-economic areas of South Africa to address the burden of NCDs, with Eat Better South Africa (EBSA) being one prominent program [31]. EBSA promotes a low-carbohydrate high-fat (LCHF) dietary approach, focusing on nutrient-dense, minimally processed foods [31]. In contrast, the South African Food-Based Dietary Guidelines (FBDG) emphasize balanced diets, with staple whole grains, moderate fat intake, and a heavy focus on plant-based foods [32]."

We appreciate the request for further elaboration on previous evaluations of the EBSA program. We have now incorporated findings in the Introduction section from three studies that assessed the program's impact on participants' perceptions, metabolic health, and adherence challenges. These studies provide important context, demonstrating both the benefits and the practical difficulties of implementing LCHF diets in under-resourced communities. The revisions ensure that our discussion aligns with existing evidence and highlights the evolving understanding of EBSA's role in community nutrition interventions.

"A mixed methods evaluation of the EBSA program showed significant improvements in metabolic health markers among participants after six months, including weight loss, reduced waist circumference, and improved glycemic control, although adherence remained a challenge due to socioeconomic factors [40]. Additionally, an earlier study reported that program participants felt empowered through nutritional education, but required ongoing support to sustain dietary changes [38]. A qualitative community assessment exploring women's willingness to participate in an EBSA program highlighted both interest in and concerns about the affordability of LCHF diets, as well as challenges related to dietary transition [39]. These findings indicate that while EBSA has had measurable success in improving metabolic health, practical barriers to long-term adherence remain, necessitating better integration with healthcare professionals and community resources."

In this case, perhaps the research problem could be specified before the objective. For example, it could be: The results on the population of this dietetic education are positive (?), but the professionals still have different opinions. What then are the opinions of the professionals on the ESBA education programme?

The objectives of the study are different and do not facilitate consistency between

objective, data collection instrument (interview) and analysis. I would propose to select only one objective, the first one, at least as a macro-objective. The others could become sub-objectives.

Response:

We appreciate the reviewer's suggestion to refine the research problem and objectives for greater clarity and consistency. We have clarified the primary objective while integrating the other objectives within the same framework to ensure alignment between the study's aim, data collection, and analysis. These revisions strengthen the connection between the research problem, objectives, and methodological approach, ensuring a clearer focus on how healthcare professionals perceive and engage with EBSA's dietary recommendations.

"While some health professionals may support LCHF diets for certain individuals or specific health conditions, others may have reservations based on concerns about long-term health effects, individual variability, sustainability, adherence, and potential nutrient deficiencies [41,42]. Although the EBSA program has shown positive outcomes in improving dietary behaviours and metabolic health among participants, healthcare professionals hold varying opinions on its dietary approach, highlighting the need to explore their perspectives in greater depth. This article aims to explore healthcare professionals' perspectives on the EBSA program, particularly focusing on the effectiveness and sustainability of the LCHF diet in managing NCDs within South Africa. While the primary objective is to examine how healthcare professionals perceive the effectiveness of the EBSA program and its dietary recommendations, this study also investigates the barriers and facilitators that influence their willingness to support or integrate EBSA dietary guidance into their practice. Furthermore, it assesses how professional training, healthcare system constraints, and exposure to evolving nutrition science shape the attitudes of these healthcare professionals toward LCHF diets and nutrition education. It examines the alignment and potential conflicts between the nutritional advice given by professionals and the guidelines promoted by EBSA, identifying gaps in nutrition education and training. This article also explores the broader public health implications of integrating such dietary recommendations and seeks to provide insights for refining the EBSA program to better meet the health needs of South Africa's diverse population."

Material and methods

Since the setting is very detailed, insert a sub-paragraph with 'setting'. Line 116

Response:

We have added a sub-paragraph titled *Setting*, which describes the context of the healthcare facilities involved in the study. This helps to better frame the environment in which data collection took place, as follows:

Participants. As a rule, participants in the study are also given a consent form for the use of the data. With this consent, anonymised and aggregated data can be disseminated. Has a particular choice been made in this respect?

Response:

We appreciate the reviewer's concern regarding participant consent for data sharing. As outlined in the Data Availability section, participants did not specifically provide consent for their transcripts to be shared publicly. The consent statement, approved by the Human Research Ethics Committee of the University of Cape Town, did not include a provision for making the data publicly available. Therefore, we do not have participant consent to share these data openly. However, requests for anonymized data can be made to the Principal Investigator, Georgina Pujol-Busquets (georgipbg@uoc.edu), in accordance with ethical guidelines. This ensures that data confidentiality and participant agreements are upheld.

Inclusion criteria: 'a community where EBSA is planning to run future programmes'. Why?

Response:

The inclusion criterion regarding "communities where EBSA plans to run future programs" was included to explore prospective opinions of healthcare professionals who may soon work with the EBSA program. This forward-looking approach provides valuable insight into how the program could be received, adapted, or challenged in the future. This has been added in the Participants section.

"Additionally, including professionals from communities where EBSA was planning future programs allowed the study to capture prospective opinions on the program, offering insight into potential facilitators and barriers before implementation."

Procedures

Line 142: There is the problem of the year of the interviews, but it has already been addressed in the introduction.

Response:

We have clarified in the text that the interviews were conducted between November 2019 and February 2020, and that this timing is acknowledged as a limitation in understanding any shifts in dietary discourse.

"This study has a temporal limitation, as data collection took place between November 2019 and February 2020, meaning the findings capture a specific moment in time. Dietary discourse in South Africa may have evolved since then, particularly due to pandemic-related shifts in food access, nutrition policies, and public health priorities. While the study provides valuable insights into health professionals' perspectives, future research is needed to explore how these views may have changed with emerging dietary trends and transition initiatives."

Line 144: I have some concerns about the sample and saturation. I prefer to talk about participants, rather than sampling, which is more typical of quantitative research. With respect to data saturation, opinions are still very diverse today. While I do not agree with what has been written, I would not change it as you have cited the literature. (I would add, however, that using thematic analysis by Braun and Clarke, these authors have written several articles on data saturation, always in a reflexive manner).

Response:

We appreciate the reviewer's concern regarding the terminology of sampling in qualitative research and the debate around data saturation in reflexive thematic analysis. In response, we have adjusted the wording in the Procedures section to emphasize that our study follows qualitative research principles that prioritize depth of insight. Additionally, we have acknowledged Braun and Clarke's position on data saturation, which views thematic development as an interpretive process rather than a point of theoretical exhaustion. This ensures that the discussion of saturation is aligned with the methodological framework used in the study.

"The IDIs were conducted between 29th of November and 21st February 2020 and lasted, on average, 30 minutes. The number of participants aligns with qualitative research recommendations, which suggest that meaningful insights can be drawn from smaller, information-rich samples [44]. According to the literature, the first five to six IDIs generally

produce the majority of new data, and the majority of concepts are identified within the first 10 interviews. As such, new participants were interviewed until saturation was reached [44]. However, we acknowledge that as outlined by Braun and Clarke, the concept of data saturation is debated, with an emphasis on depth of interpretation rather than a fixed threshold for thematic exhaustion [45].

Line 149. It would be important to explain how the interview was constructed.

Response:

We appreciate the reviewer's concern and a new paragraph has been added to the Procedures section:

"The interview guide was developed based on a review of the literature on healthcare professionals' perceptions of dietary interventions, particularly low-carbohydrate diets. It was piloted with two health professionals (not included in the final sample) to ensure the relevance, clarity, and cultural appropriateness of the questions. The final guide followed a semi-structured format, allowing for follow-up questions and probing to explore emerging topics in depth. It covered several key areas: healthcare providers' perceptions of their role in community health; major health concerns identified in their communities; views on non-NCDs such as obesity, diabetes, and cardiovascular disease; the type of dietary advice offered at their healthcare facilities; perspectives on the role of nutrition in chronic disease prevention and treatment; their understanding of local dietary patterns; and their awareness and impressions of the EBSA program and the LCHF diet, including views on the feasibility of implementing such interventions in their communities."

Line 154. Ok the deregistration with software, but as you have reviewed the transcripts, I would ask you if you have pointed out the researchers' field notes or if you have retained part of the participants' paraverbal communication, useful elements for the analysis.

Response:

Thank you for this insightful observation. We have now clarified in the Data Analysis section that although field notes were not formally incorporated into the coding framework, researchers actively documented observations during and immediately after each interview. These notes captured contextual and paraverbal elements—such as tone of voice, significant

pauses, and emphasis—that, while not coded directly, played an important role in informing and enriching our interpretative process. This documentation supported a deeper understanding of participants' responses and contributed to the overall reflexivity of the analysis.

"During and after the interviews, the research team systematically recorded observations and took field notes, including contextual insights and paraverbal cues such as tone, pauses, and emphasis. Although this data was not formally coded, it was used in the process of interpreting and understanding the participants' narratives and in the development of the themes".

Line 159: Table 1: I would suggest moving the detailed, very thorough interview to the annex. In the text insert a summary of the interview, reworked with main subject areas and, for each area, one or two sample questions.
Response:

Thank you for this suggestion. The full interview guide has now been moved to a supplementary appendix, and the text in the Procedures section includes a summarized version highlighting key topic areas.

"The interview guide was developed based on a review of the literature on healthcare professionals' perceptions of dietary interventions, particularly low-carbohydrate diets. It was piloted with two health professionals (not included in the final sample) to ensure the relevance, clarity, and cultural appropriateness of the questions. The final guide followed a semi-structured format, allowing for follow-up questions and probing to explore emerging topics in depth. It covered several key areas: healthcare providers' perceptions of their role in community health; major health concerns identified in their communities; views on NCDs such as obesity, diabetes, and cardiovascular disease; the type of dietary advice offered at their healthcare facilities; perspectives on the role of nutrition in chronic disease prevention and treatment; their understanding of local dietary patterns; and their awareness and impressions of the EBSA program and the LCHF diet, including views on the feasibility of implementing such interventions in their communities."

Before analysing the data, you should include which instrument was used for reporting the qualitative research. On the basis of the chosen instrument, a paragraph on the research team could be inserted, specifying the expertise in qualitative research.

Response:

We appreciate the reviewer's suggestion to specify the qualitative reporting instrument and provide information on the research team's expertise. In response, we have stated in the Data Analysis section that the study followed the COREQ guidelines to ensure comprehensive and transparent qualitative reporting. Additionally, in the Materials and Methods section, we have included a sentence about the research team's expertise in qualitative research and analysis, highlighting how the involvement of multiple researchers strengthened the findings.

"The study adhered to the Consolidated Criteria for Reporting Qualitative Research (COREQ) guidelines to ensure that the methods and findings were reported in a transparent and rigorous manner [48]."

Reference

48. Tong A, Sainsbury P, Craig J. Consolidated criteria for reporting qualitative research (COREQ): a 32-item checklist for interviews and focus groups. *Int J Qual Health Care.* 2007;19(6):349-357. doi:10.1093/intqhc/mzm042.

Data Analysis

Line 165. You cite Braun and Clarke (2006) and it is correct, but these authors have also written extensively on thematic analysis in very recent years. One could update the literature.

Response:

Thank you for your suggestion. As we have explained in an earlier response, we followed the foundational guidance provided by Braun and Clarke (2006) in our analysis, which is more aligned with the procedures implemented in this study than their recent contributions (i.e., use of a collaboratively developed codebook and independent double coding), while still being a widely cited and relevant approach for conducting thematic analysis. We have clarified this in the manuscript to ensure methodological consistency and transparency.

"The 2006 version of Braun and Clarke's thematic analysis was used. This version is characterised by its epistemological flexibility and openness to the use of codebooks, a feature that has since been abandoned by the authors in more recent iterations of this analytical method [45]."

Line 169. The provision of codebooks for qualitative research is very good. I would suggest placing the basic data from which the analysis is developed in a repository linked to the article.

Response:

Thank you for this helpful suggestion. As noted in the Data Availability section, participant consent did not include permission to share transcripts or raw data publicly. In accordance with the confidentiality agreements approved by the Human Research Ethics Committee of the University of Cape Town, we are therefore unable to make the basic data available in a public repository.

Regarding the use of a codebook approach, while we appreciate that its inclusion supports transparency, we note that Braun and Clarke (2022) have more recently distanced themselves from the use of codebooks in reflexive thematic analysis. They argue that the use of rigid codebooks can be at odds with the flexible, evolving nature of coding in reflexive approaches. That said, for the sake of transparency and clarity, we are open to providing a descriptive and anonymized version of the codes developed during analysis upon reasonable request.

Line 179. Thematic analysis (by Braun &Clarke) is developed into themes and sub-themes. The transition to concepts is more titpical of grounded theory. I would relate the description of the analysis to your chosen model (themes and sub-themes)
The description of the analysis is very detailed and offers many insights into the rigour of the research. However, I would suggest pointing out a reference author with a citation for rigour (by including it as a citation in the analysis) or make a separate paragraph pointing out which dimensions of rigour you specifically considered (with reference author).

Response:

Thank you for your thoughtful feedback. We have revised the Methods section substantially to clarify our analytical approach and ensure alignment with the model we used. Specifically, we have described our process as a codebook approach to thematic analysis guided by Braun and Clarke's original 2006 framework, acknowledging that our approach does not follow the reflexive thematic analysis approach outlined in their recent publications. Accordingly, we

now refer consistently to themes and sub-themes rather than "concepts," to remain methodologically consistent with thematic analysis rather than grounded theory.

In addition, we have expanded the description of how rigour was ensured throughout the study. Rather than referring to inductive analysis as a marker of rigour, we have added appropriate citations to support this section. We have clarified that we used the COREQ checklist [48] to strengthen reporting transparency and added references accordingly—see references 45, 47, and 48.

References:

45. Braun V, Clarke V. Thematic analysis: A practical guide. London: SAGE Publications; 2022.

47. Bloor M. Techniques of validation in qualitative research: a critical commentary. In: Miller G, Dingwall R, editors. Context and method in qualitative research. London: SAGE Publications; 1997. p. 37–50.

48. Tong A, Sainsbury P, Craig J. Consolidated criteria for reporting qualitative research (COREQ): a 32-item checklist for interviews and focus groups. Int J Qual Health Care. 2007;19(6):349-357. doi:10.1093/intqhc/mzm042.

Results

Line 199. I would enter the total number of respondents in the introduction of the results. I would then imagine that a code, e.g. numeric, was assigned to each participant, while maintaining the difference in profession (Cod. Ph. 1 to indicate the first code, e.g. Physician)

Response:

We agree with this suggestion and have revised the Results introduction. This coding system is now used consistently throughout the Results section to label quotations.

"A total of 16 healthcare professionals participated in the study, including 7 medical physicians, 7 nurses, and 2 dietitians. To ensure transparency and traceability in the reporting of quotes, each participant was assigned a code reflecting their profession and participant number (e.g., MPh.1 for Male Physician 1, FN.1 for Female Nurse 1, FD.1 for Female Dietitian 1)."

I would rework the table of themes, keeping the 4 main themes, but also inserting the

sub-themes (which can be derived from the definitions) for each theme. The quotes are too many and too long (they should be an example), so I suggest selecting them and reducing them in length. For example.
Themes Sub-themes quotations
1. Foundations of dietary understanding
1.1. knowledge of nutritional guidelines, assessment.. (e.g.) 'We basically follow the protocols of the National Health Department and (..) there isn't really much with regards to prevention, they have information, but it's outdated (..) (Male physician).
1.2. dietary counselling and nutrition education
Response:

We have revised the Table 1 of themes to include sub-themes derived directly from the analysis. We have also carefully reviewed the quotes used in the Table 1 of the Results section, and have selected only the most illustrative and impactful examples for each theme. Each quote is short excerpt that conveys the core insight, while ensuring the participant's profession and code are attached.

Text of Results
The text of the results could also be reworked with themes and sub-themes. It would make comprehension more immediate, in my opinion.
I really appreciate it when the codes are included in the text in full or as parts of the quotes (e.g. lines 247-249, 252..). I would however add the code.

Response:
We appreciate the reviewer's suggestion to present the results with themes and sub-themes more explicitly for clarity. To address this, we have Table 1 that clearly outlines themes, sub-themes, definitions and illustrative quotes, allowing for a structured presentation of findings. However, in the Results section, we have chosen to present the findings at the theme level to maintain a coherent narrative flow and avoid excessive fragmentation of the discussion. This approach balances structured thematic presentation with a readable and engaging results section, ensuring that key findings remain accessible.

Line 287. 'For those who did not know about the diet..' to remedy this, the fact of knowing about this diet could be included in the inclusion criteria.
Response:

We appreciate the reviewer’s suggestion to include prior knowledge of the LCHF diet as part of the inclusion criteria. However, our inclusion criteria focused on selecting healthcare professionals working in communities where EBSA had implemented or planned to implement its nutrition education program, rather than requiring familiarity with the LCHF diet itself. This approach allowed us to capture a diverse range of perspectives, including those of professionals unfamiliar with LCHF, providing insights into both supportive and skeptical viewpoints. Additionally, as noted in the Participants section, while participants were selected from facilities in relevant communities, this did not guarantee their direct involvement in or awareness of the EBSA program or its dietary recommendations. Including professionals from communities where EBSA is planning future programs also allowed us to explore prospective opinions on the program’s feasibility and acceptance. This variation in awareness and familiarity with LCHF is an important part of our findings, highlighting the challenges in knowledge dissemination and the need for further professional education on different dietary approaches. Therefore, we believe maintaining the existing inclusion criteria was appropriate for the study’s objectives.

Line 389. I would not include citations in the results

Response:

Thank you for your valuable feedback. We have carefully reviewed the Results section and have rewritten Theme 4 to ensure that it is solely based on participant insights, removing references 49-52. Any necessary references from this section have been relocated to the Discussion where they provide additional context to our findings. This revision enhances the clarity and integrity of the results by ensuring that all statements are directly derived from the qualitative data. We appreciate this insightful suggestion and believe it strengthens the manuscript.

The results, which are very extensive, go far beyond the professionals' view of the ESBA education programme, which is little or not at all known to the professionals interviewed. The various problems highlighted in the results concern the ESBA programme only in part, because they then range over the whole aspect of nutrition education, in poor populations for whom the food problem is not the priority, the professionals‘ lack of knowledge, their need for training, and above all the professionals’ doubts as to whether or not this programme is effective and how to do

effective nutrition education, and the role of women in African communities. Even the title, with these results, is no longer explanatory. I therefore suggest that we seek consistency between the title, objectives and results, selecting results that express clarity on what is achieved with this study. It seems to me that the main underlining feature at the moment is the confusion of the professionals as to which programme to focus on and the request to be informed about nutrition education so as to be of help to the population. The conclusions follow from what has been said for the results.

Response:

Thank you for your thoughtful feedback. In response to your comments and another reviewers' ones, we have revised the title and rewritten the objectives to ensure greater consistency between the study's aims and its findings. The updated objectives now better reflect the broader themes that emerged from the interviews, including health professionals' confusion about nutrition education, their need for further training, and their uncertainty about the effectiveness of the EBSA program within the healthcare system.

Additionally, we have refined a lot of the Results section to ensure that it remains focused on the study's key objectives while still addressing the broader context of nutrition education in under-resourced settings. By clarifying the primary research aim, exploring healthcare professionals' perspectives on EBSA and their knowledge gaps in nutrition education, we ensure that the findings align with the study's overall purpose.

We appreciate this insightful recommendation, as it has helped us strengthen the manuscript's coherence and focus.

The revised title is:

"Between support and skepticism: Health professionals' perceptions on a nutrition education program promoting Low-Carbohydrate, High-fat diets in under-resourced South African communities."

The Conclusion has also been rewritten to focus explicitly on the study's contributions, see the revised section:

"This study provides valuable insights into healthcare professionals' perspectives on a nutrition education program recommending LCHF diets, highlighting key challenges in their understanding and implementation within under-resourced South African communities. Professionals expressed uncertainty around LCHF, often shaped by conflicting guidelines, limited exposure to recent evidence, and scepticism about its long-term effects. The findings

also reveal significant training gaps in nutrition education, with many professionals, mostly doctors and nurses, lacking confidence in providing dietary guidance beyond conventional recommendations. Additionally, socioeconomic barriers such as food affordability, cultural norms, and structural inequalities further complicate dietary behaviour change in these communities. Despite these challenges, according to participants, EBSA presents a promising model for integrating nutrition education into healthcare strategies, particularly by offering structured support and practical guidance tailored to local contexts. Future efforts should focus on bridging knowledge gaps, strengthening professional training, and ensuring that dietary interventions are both evidence-based and accessible to those who need them most."

6. PLOS authors have the option to publish the peer review history of their article (what does this mean?<https://track.editorialmanager.com/CL0/https:%2F%2Fjournals.plos.org%2Fplosone%2Fs%2Feditorial-and-peer-review-process%23loc-peer-review-history/1/010f019547d21ab6-08e7bc50-c91e-4e43-a149-14affdf0e8f0-000000/XI4asZMiNT-0EPVAjO5moDp19tutEvwUtzZofPwF3tU=200>). If published, this will include your full peer review and any attached files.
If you choose "no", your identity will remain anonymous but your review may still be made public.
Do you want your identity to be public for this peer review? For information about this choice, including consent withdrawal, please see our Privacy Policy<https://track.editorialmanager.com/CL0/https:%2F%2Fwww.plos.org%2Fprivacy-policy/1/010f019547d21ab6-08e7bc50-c91e-4e43-a149-14affdf0e8f0-000000/RHicORkQCDHbpzx7TsW5gop56rrq6WXp5tKo5ZFwpc0=200>.

Reviewer #1: No

Reviewer #2: No

Reviewer #3: Yes: Abdelah Alifnur Mohammed

Reviewer #4: No

[NOTE: If reviewer comments were submitted as an attachment file, they will be

attached to this email and accessible via the submission site. Please log into your account, locate the manuscript record, and check for the action link "View Attachments". If this link does not appear, there are no attachment files.]
While revising your submission, please upload your figure files to the Preflight Analysis and Conversion Engine (PACE) digital diagnostic tool, https://pacev2.apexcovantage.com/<https://track.editorialmanager.com/CL0/https:%2F%2Fpacev2.apexcovantage.com%2F/1/010f019547d21ab6-08e7bc50-c91e-4e43-a149-14affdf0e8f0-000000/qIu5THaYeTAR8UHYhmKIhKp-Eqwi3kurqp3yQi67OPM=200>. PACE helps ensure that figures meet PLOS requirements. To use PACE, you must first register as a user. Registration is free. Then, login and navigate to the UPLOAD tab, where you will find detailed instructions on how to use the tool. If you encounter any issues or have any questions when using PACE, please email PLOS at figures@plos.org<mailto:figures@plos.org>. Please note that Supporting Information files do not need this step.
In compliance with data protection regulations, you may request that we remove your personal registration details at any time. (Remove my information/details)<https://track.editorialmanager.com/CL0/https:%2F%2Fwww.editorialmanager.com%2Fpone%2Flogin.asp%3Fa=r/1/010f019547d21ab6-08e7bc50-c91e-4e43-a149-14affdf0e8f0-000000/uRQRV5v6A1j4LvS-xuljxaCPXWqS-wEDnsuXRxkgpYo=200>. Please contact the publication office if you have any questions.[http://track.editorialmanager.com/CI0/010f019547d21ab6-08e7bc50-c91e-4e43-a149-14affdf0e8f0-000000/wujjvCxXJxP7JYvFv3lar32WD_R8z_Q0gRxkc0axlZk=200]
