# Supplementary material for: Between support and scepticism: Health professionals’ perceptions of a nutrition education program promoting low-carbohydrate, high-fat diets in under-resourced South African communities
Source: PLoS One. 2025 Jun 23;20(6):e0325179. doi: 10.1371/journal.pone.0325179 (PMC12184999; doi:10.1371/journal.pone.0325179)
Supplement: Attachment — Submitted filename: Review comments 1.doc [file pone.0325179.s002.doc]

Review comments

Title: Between support and skepticism :Health professional's views on low carbohydrate diest in under-resourced South African communities.

The paper is written well except the following gaps;

1.Content or focus: much of professional/expert skepticism is raised against the applicability or practicability of EBSA's LCHF program ,not on its health implications. All the issues under theme 4 are arguments questioning practicability.Thus, it would have been better the research was framed along that direction.

2.Your findings go beyound the scope of your title. Your title is restricted only to Low Carbohydrate Diets, but your findings and discussions are focusing more on the controvercies over High Fat Diets. Look at the folowing on the sources of skepticism;

line 35, 303, 311-312, and 420. These all are stressing on expert caution on High Fat diets than Low Carbohydrates.

3.Unsupported and contradictory statements. For example,

A)health professional opinions on the role of nutritional education, (line 2002-2003 contradicts with (line 421_422).

B)information access of professionals,(line 229 contradicts with line 430).

C)popularity of LCHF diets in south africa (504) contradicts with lack of support and stopping to follow those diets mentioned in the manuscript

D.effectiveness of LCHF, consensus guidelines on LCHF (line 74)Vs lack of scientific consensu on it (309)

E. Venue of meeting interviewee: private venue(line 137) Vs in the health facilities(519)

F.Duration of contact with interviewees: one hour(137) Vs thirty minutes(141)

G.selection of interviwee was done from facilities that run EBSA program nutrition education (113). That means they are expected to be knowledgabe about the program.However, you have written ( line 329-330) that "majority were introduced to (EBSA) program for the first time during the interview"

4) lack of clarity on the role of science: line 309 and 221 which similarly state that "confusing information comes from science". These two contradict with line 226 "few health professjonals made any reference to science". Does science resolve doubts or produces and ignites them? It also begs the question , if most of the experts are not refering science, why and how do they can be confused with scientific evidences on the topic?

5). Tha dat collecton (for this research) conducted in nov 2019 and the corresponding findings might not be of high relevance to adress the new dietary trends, including dietary transition initiatives.

6).inaccurate targeting of the sample population. The right targets should be nutritionists/dieticians. Not only that, they also should not be from private facilities.

Generally, the paper has methodological and content gaps that need to be corrected.
