# Supplementary material for: Between support and scepticism: Health professionals’ perceptions of a nutrition education program promoting low-carbohydrate, high-fat diets in under-resourced South African communities
Source: PLoS One. 2025 Jun 23;20(6):e0325179. doi: 10.1371/journal.pone.0325179 (PMC12184999; doi:10.1371/journal.pone.0325179)
Supplement: Attachment — Submitted filename: Response to Reviewers.docx [file pone.0325179.s004.docx]

The authors have showed a concerned engagement with the comments and tried to answer questions and address the comments. But I still have the following queries;

1. The issue of data availability; I did not see a plausible justification from the authors
2. The EBSA program vs professional views still needs clarification
3. I also did not feel that all researchers have equally engaged and contributed in the original research work
