# Supplementary material for: Between support and scepticism: Health professionals’ perceptions of a nutrition education program promoting low-carbohydrate, high-fat diets in under-resourced South African communities
Source: PLoS One. 2025 Jun 23;20(6):e0325179. doi: 10.1371/journal.pone.0325179 (PMC12184999; doi:10.1371/journal.pone.0325179)
Supplement: Attachment — Submitted filename: plos review.docx [file pone.0325179.s005.docx]

**Response to Reviewers’ comments**

Manuscript Title: *Between support and scepticism: Health professionals’ views on Low-Carbohydrate diets in under-resourced South African communities*.

New Tile: *Between support and scepticism: Health professionals’ perceptions on a nutrition education program promoting Low-Carbohydrate, High-fat diets in under-resourced South African communities.*

Manuscript ID: PONE-D-24-55230

Dear Editor and Esteemed Reviewers,

On behalf of all co-authors, I would like to sincerely thank the reviewers for their thoughtful and constructive feedback on our manuscript. We greatly appreciate the time and expertise that went into reviewing our work, and we are confident that the suggested revisions have significantly improved the quality and clarity of our submission. We have carefully considered each comment and have responded in detail below, outlining the changes made to the manuscript and providing clarifications where necessary.

We trust that our revisions have addressed the reviewers' concerns, and we look forward to your further consideration of our manuscript for publication in *PLOS ONE*.

Sincerely,

Georgina Pujol-Busquets

**Reviewers' comments:**

**Comments to the Author**

**1. Is the manuscript technically sound, and do the data support the conclusions?

The manuscript must describe a technically sound piece of scientific research with data that supports the conclusions. Experiments must have been conducted rigorously, with appropriate controls, replication, and sample sizes. The conclusions must be drawn appropriately based on the data presented.

Reviewer #1: Yes

Reviewer #2: No

Reviewer #3: Yes

Reviewer #4: Partly

2. Has the statistical analysis been performed appropriately and rigorously?

Reviewer #1: N/A

Reviewer #2: No

Reviewer #3: Yes

Reviewer #4: N/A

3. Have the authors made all data underlying the findings in their manuscript fully available?

The PLOS Data policy<**[**http://track.editorialmanager.com/CL0/http:%2F%2Fwww.plosone.org%2Fstatic%2Fpolicies.action%23sharing/1/010f019547d21ab6-08e7bc50-c91e-4e43-a149-14affdf0e8f0-000000/uoBneyU2ESJ2opnWfelmiRHTzi2lhcmhawIbFSjISQo=200**](http://track.editorialmanager.com/CL0/http:%2F%2Fwww.plosone.org%2Fstatic%2Fpolicies.action%23sharing/1/010f019547d21ab6-08e7bc50-c91e-4e43-a149-14affdf0e8f0-000000/uoBneyU2ESJ2opnWfelmiRHTzi2lhcmhawIbFSjISQo=200)**> requires authors to make all data underlying the findings described in their manuscript fully available without restriction, with rare exception (please refer to the Data Availability Statement in the manuscript PDF file). The data should be provided as part of the manuscript or its supporting information, or deposited to a public repository. For example, in addition to summary statistics, the data points behind means, medians and variance measures should be available. If there are restrictions on publicly sharing data—e.g. participant privacy or use of data from a third party—those must be specified.

Reviewer #1: No

Reviewer #2: No

Reviewer #3: No

Reviewer #4: No

4. Is the manuscript presented in an intelligible fashion and written in standard English?

PLOS ONE does not copyedit accepted manuscripts, so the language in submitted articles must be clear, correct, and unambiguous. Any typographical or grammatical errors should be corrected at revision, so please note any specific errors here.

Reviewer #1: Yes

Reviewer #2: Yes

Reviewer #3: Yes

Reviewer #4: Yes

5. Review Comments to the Author**

**Please use the space provided to explain your answers to the questions above. You may also include additional comments for the author, including concerns about dual publication, research ethics, or publication ethics. (Please upload your review as an attachment if it exceeds 20,000 characters)**

**Responses to Reviewers’ Questions**

**Reviewer #1: This study explores healthcare professionals' perspectives on nutrition and the EBSA program, drawing on 16 in-depth individual interviews with physicians, nurses, and dietitians from False Bay Hospital, Groote Schuur Hospital, and a primary health clinic in Hout Bay, Western Cape, South Africa. Thematic analysis revealed four main theme- Foundations of dietary understanding, LCHF: Controversies and beliefs; EBSA’s role in community nutrition; and understanding socioeconomic determinants of health. It is nicely written, and I have no major concerns. I offer the following suggestions for your consideration:**
**1. While I absolutely agree with the first paragraph, I felt that it didn’t align well with the study performed. Can this be more focussed on the research question? Or, perhaps, just delete it and start with the sentence in line 70 (“Despite nutrition’s…”)**

**Response:**
We agree that the first paragraph did not fully align well with the study focus. As suggested, the paragraph has been modified, and the Introduction now begins with:
"South Africa's public health system faces major challenges, including long wait times, resource shortages, poor management, inadequate infection control, and workforce deficits—exacerbated by inequitable resource distribution, rapid urbanization, and the dual burden of infectious and non-communicable diseases (NCDs) [1–9].”

**2. What would be useful to a reader not intimately familiar with SA nutrition/health systems would be a brief (just brief, no need for >4 sentences) comparison of EBSA vs. standard dietary guidance in South Africa. Could this be added to provide this context?**

**Response:**
We have added a brief comparison between EBSA and standard dietary guidance in South Africa in the Introduction. This contextual framing is now included to orient readers unfamiliar with South African dietary policy.

“EBSA promotes a low-carbohydrate high-fat (LCHF) dietary approach, with an emphasis on nutrient-dense, minimally processed foods [31]. The LCHF approach contrasts with the South African Food-Based Dietary Guidelines, which emphasise balanced diets with staple whole grains, moderate fat intake, and a heavy focus on plant-based foods [32].”

Reference:
32. Vorster HH, Badham JB, Venter CS. An introduction to the revised South African food-based dietary guidelines. S Afr J Clin Nutr. 2013;26(Suppl 1):S5–S12.

**3. Was only selecting English speaking participants a major bias?**

**Response:**
We acknowledge this as a limitation and have now explicitly mentioned this in the limitations section at the of the Discussion, noting that the exclusion of non-English speakers may have limited the diversity of perspectives captured.

“The diverse perspectives of physicians, nurses, and dietitians offer a comprehensive view, but the uneven sample size, limited facility access, the affiliation of one dietitian with an NGO, and the exclusion of non-English speakers may have limited the diversity of perspectives captured and introduced biases. Future studies should include non-English-speaking health professionals to capture broader cultural perspectives.”

**4. Why is “original language” stated in line 153? Were all interviews not conducted in English, as stated in line 137-38?**

**Response:**
This has been corrected to explicitly state that all interviews were conducted in English. The term “original language” has been removed to avoid confusion.

**5. Was member checking performed? If not, might be good to justify why it was not done.**

**Response:**
Member checking was not performed in this study due to both logistical and methodological considerations. Practically, the time and resource constraints inherent in the research timeline made it infeasible to re-engage participants for feedback. However, beyond these constraints, there are also epistemological and methodological concerns associated with the use of member checking in qualitative research of this nature. One common critique, as discussed by Bloor (1997), is that member validation exercises are not neutral or definitive tests of validity, but rather complex social encounters influenced by context, participant subjectivity, and the changing nature of perception. As Bloor demonstrates, members’ reactions to findings are often shaped by the passage of time, evolving perspectives, and the dynamics of the feedback process itself. Participants might reject or revise earlier statements not because the initial data were invalid, but because their current viewpoints differ, highlighting the inherently dynamic and situated nature of perceptions. Furthermore, member checking has been critiqued for its roots in positivist assumptions about data validation—that there is a singular, fixed “truth” that participants can confirm. In interpretive qualitative paradigms, such as the one guiding this study, the researcher acknowledges that data are co-constructed and context-dependent, and that seeking verification from participants can sometimes undermine the complexity and multiplicity of meanings present in qualitative data (Bloor, 1997).

Therefore, the decision not to employ member checking aligns with both practical limitations and a reflexive approach that prioritizes depth of interpretation over post hoc validation by participants.

“We did not use member checking for practical and methodological considerations. Logistically, time and resource constraints prevented following up with participants. Methodologically, we adopted a reflexive approach that recognises the dynamic and contextual nature of participants’ perceptions, which may shift over time and are not necessarily fixed or singular. Participant feedback is shaped by the circumstances in which it is elicited and does not constitute a definitive test of validity [47].”

Reference:
47. Bloor M. Techniques of validation in qualitative research: a critical commentary. In: Miller G, Dingwall R, editors. *Context and method in qualitative research*. London: SAGE Publications; 1997. p. 37–50.

**6. Which profession usually counsels on nutrition in this setting? If it’s dietitians, would having only 2/16 participants as dietitians be a limitation of perspective?**

**Response:**
We have clarified in the limitations section in the Discussion that while dietitians are typically the lead providers of dietary counselling, nurses and physicians in under-resourced primary care settings often deliver nutrition advice due to staff shortages. This sampling choice, therefore, reflects real-world task-shifting common in South African healthcare.

“Additionally, while dietitians are typically the primary providers of dietary counselling, in under-resourced primary care settings, nurses and physicians often take on this role due to staff shortages [52]. This reflects the real-world task-shifting that is common in South African healthcare, where non-specialist providers must frequently step in to deliver nutrition advice. While dietitians play a central role in dietary counselling, this study included physicians and nurses to capture how nutrition messages are communicated in primary care, aligning with community health models for under-resourced settings. Future research focusing solely on dietitians could provide more specialized insights into LCHF counselling and implementation.”

**7. Discussion line 448- could you provide an example of what is meant by “backhanded support”?**

**Response:**
This phrase has been clarified in the Discussion to mean that professionals reluctantly supported patients who chose LCHF diets, not because they endorsed the diet themselves, but because they wanted to encourage patient autonomy. This illustrates a phenomenon known as therapeutic pragmatism, where professionals adopt a supportive stance despite personal doubts.

“Their reluctance stemmed from concerns about the diet’s alignment with conventional dietary guidelines, a lack of confidence in providing LCHF-specific advice, and uncertainty about its long-term health impacts. Rather than actively endorsing LCHF diets, professionals supported patients out of a desire to respect patient autonomy, a stance known as therapeutic pragmatism, where healthcare providers adopt a supportive role despite personal doubts [59]. This meant that while they outwardly agreed to assist patients following an LCHF approach, their hesitance and underlying scepticism often resulted in a lack of meaningful guidance, leaving patients to navigate dietary changes largely on their own.”

Reference:
59. Pilnick A, Hindmarsh J, Gill VT, editors. Communication in healthcare settings: policy, participation and new technologies. Chichester: Wiley-Blackwell; 2010. 160 p. ISBN: 978-1-4051-9827-1.

**8. Discussion line 448-450- this is an interesting and possibly loaded interpretation. Could you expand? Is it that the doctors believed in LCHF (despite admitting limited nutrition knowledge), and were reluctant (“sometimes”) to send to dietitians because they feared that dietitians would not provide LCHF advice?**

**Response:**
This section has been expanded at the end of the Discussion to explain that some doctors privately believed in LCHF’s potential benefits (often informed by media rather than training), but were reluctant to refer to dietitians because they expected dietitians to discourage LCHF in favor of conventional low-fat advice.

“Despite these challenges, the study offers valuable insights into health professionals' attitudes towards nutritional advice and LCHF diets, contributing to discussions on community healthcare and NCD management. Notably, some doctors privately believed in LCHF’s potential benefits—often informed more by media than formal training—but were reluctant to refer patients to dietitians, anticipating that dietitians would discourage LCHF in favor of conventional low-fat advice [13].”

**9. Discussion lines 459-464; could you clarify- was the failure to endorse LCHF in communities one of the group, in that the support group itself (was this composed of patients) was not endorsing LCHF? It’s not clear**

**Response:**
This has been revised to clarify in the Discussion that some professionals felt community-based support groups themselves were hesitant to fully endorse LCHF diets. This stemmed from uncertainty, conflicting advice from different health workers, and broader scepticism about non-traditional diets within these groups.

“Many healthcare professionals remained cautious about formally supporting the diet, reflecting a broader global trend where media-driven interest does not always translate into clinical adoption [60]. This suggests that scepticism about LCHF is not solely a matter of questioning its health implications but rather an uncertainty about how it can be practically adopted in resource-limited settings. Some professionals also noted that hesitation toward LCHF did not come solely from healthcare providers but was also present within the support groups themselves, which were composed of community members, including patients. This reluctance within the groups stemmed from conflicting advice received from different health workers, scepticism about non-traditional diets, and broader uncertainty about the feasibility of LCHF within low-income settings [61-63]. While some professionals acknowledged that the program might be effective in changing perceptions about diet, concerns about the LCHF approach were also raised.”

**Reviewer #2:**

**1. May please add some of the observations in the tabulated form.**

**Response:**
Thank you for your suggestion. Table 2 (now Table 1) was developed initially to provide deeper insights into the themes, offering a structured view of health professionals' perspectives with definitions and direct quotes to support the qualitative analysis. By summarizing key findings while maintaining participant perspectives, we think that Table 1 effectively presents observations in a clear, accessible format. We believe it meets the request but welcome further clarification if needed.

**2. It would be better if you could convert the qualitative discussion output as quantitative values, which can be statistically analyzed.**

**Response:**
Thank you for the comment. While some qualitative studies may include elements of quantification, we do not consider it appropriate in the context of this particular study. The approach employed is interpretive, where the aim is to explore meaning and complexity rather than to measure frequency.

**3. Sentences may be edited for clarity.**

**Response:**
The manuscript has undergone thorough language editing to improve clarity and readability.

**4. The presented conclusion could be part of the discussion, and a new conclusion based on the study conducted may be developed.**

**Response:**

Thank you for your comment. The Conclusion has been rewritten to focus explicitly on the study’s contributions, see the revised section:

“This study provides valuable insights into healthcare professionals’ perspectives on a nutrition education program recommending LCHF diets, highlighting key challenges in their understanding and implementation within under-resourced South African communities. Professionals expressed uncertainty around LCHF, often shaped by conflicting guidelines, limited exposure to recent evidence, and scepticism about its long-term effects. The findings also reveal significant training gaps in nutrition education, with many professionals, mostly doctors and nurses, lacking confidence in providing dietary guidance beyond conventional recommendations. Additionally, socioeconomic barriers such as food affordability, cultural norms, and structural inequalities further complicate dietary behaviour change in these communities. Despite these challenges, according to participants, EBSA presents a promising model for integrating nutrition education into healthcare strategies, particularly by offering structured support and practical guidance tailored to local contexts. Future efforts should focus on bridging knowledge gaps, strengthening professional training, and ensuring that dietary interventions are both evidence-based and accessible to those who need them most.”

**5. Was there any control group?**

**Response:**
As an exploratory qualitative study, no control group was used, as this approach aligns with the principles of qualitative research, which prioritise an in-depth understanding of the phenomena under investigation over comparative analysis (Yin 2011).

Reference:
Yin, R. K. (2011). *Qualitative research from start to finish.* Guilford Press.

**Reviewer #3: Review comments
Title: Between support and scepticism :Health professional's views on low carbohydrate diest in under-resourced South African communities.
The paper is written well except the following gaps;**
**1.Content or focus: much of professional/expert skepticism is raised against the applicability or practicability of EBSA's LCHF program ,not on its health implications. All the issues under theme 4 are arguments questioning practicability.Thus, it would have been better the research was framed along that direction.**

**Response:**

We appreciate the reviewer’s insightful feedback and agree that much of the skepticism raised by professionals focused on the practical applicability of EBSA’s LCHF program rather than purely on concerns about health implications. However, we believe that both aspects—uncertainty about the diet’s feasibility in under-resourced settings and concerns about its long-term health effects—were present in the data. To align with the reviewer’s suggestion, we have revised the Discussion section to ensure that implementation challenges—such as affordability, accessibility, and food security—are clearly emphasized as primary concerns among professionals. We have clarified that practical barriers, rather than outright rejection of LCHF as a nutritional intervention, were central to skepticism. However, we have also retained references to health-related concerns, as some professionals questioned the sustainability and long-term impact of LCHF on metabolic health, particularly in patients with comorbidities. We appreciate this valuable feedback and believe these refinements strengthen the clarity of our findings. We hope this revision adequately addresses the concern, but we welcome any further suggestions for improvement. Please see the track changes version of the manuscript to see all the changes in the Discussion.

**2.Your findings go beyound the scope of your title. Your title is restricted only to Low Carbohydrate Diets, but your findings and discussions are focusing more on the controvercies over High Fat Diets. Look at the folowing on the sources of skepticism;
line 35, 303, 311-312, and 420. These all are stressing on expert caution on High Fat diets than Low Carbohydrates.**

**Response:**
We appreciate this observation and have revised the title to better reflect the scope of the study, incorporating both *low carbohydrate* and *high fat* aspects to align with the term “LCHF” (Low-Carbohydrate, High-Fat). This dual emphasis is critical as it captures the full nature of the dietary recommendations under review, consistent with international definitions of LCHF diets. Please see the Title Page document for the revised version.

Revised Title: “*Between support and scepticism: Health professionals’ perceptions on a nutrition education program promoting Low-Carbohydrate, High-fat diets in under-resourced South African communities.”*
 **3.Unsupported and contradictory statements. For example,
A)health professional opinions on the role of nutritional education, (line 2002-2003 contradicts with (line 421_422).**

**Response:**
Thank you for pointing out this inconsistency regarding health professionals' views on nutritional education. We recognize that the statements in line 202-203 and line 421-422 could be interpreted as contradictory. To clarify, professionals acknowledged the importance of nutritional education in disease prevention but simultaneously expressed challenges in integrating it into their routine practice due to time constraints and limited expertise. Their recognition of its importance does not necessarily translate into their ability or confidence to provide such education effectively.

To resolve this contradiction, we have revised the Discussion.

“While physicians and nurses recognized the value of nutritional education in disease prevention, their ability to provide effective dietary counselling was hindered by systemic barriers, including time constraints, competing clinical responsibilities, and inconsistent nutritional messaging. [50].”

**B)information access of professionals,(line 229 contradicts with line 430).**

**Response:**

We appreciate the reviewer’s observation regarding the apparent contradiction in how professionals discuss access to nutritional information. We acknowledge that while professionals reported struggles in keeping up with evolving nutrition science, they simultaneously relied on established dietary concepts that were not always critically examined. To address this, we have revised the Discussion section to clarify that health professionals acknowledged the need for more clinical trials to strengthen evidence-based dietary guidelines but continued to use established dietary terminology without necessarily scrutinizing its scientific basis. This revision ensures that both aspects, their difficulty in staying updated with nutrition science and their reliance on conventional dietary messaging, are accurately represented. To resolve this contradiction, we have revised the Discussion.
“While health professionals expressed difficulty in staying updated with advancements in nutrition science, they also relied heavily on widely accepted dietary principles without necessarily questioning or critically analyzing them. Furthermore, many professionals agreed that more clinical trials were needed to reassess current dietary guidelines, reflecting an awareness of the evolving nature of nutrition science. This duality, acknowledging the need for stronger evidence while continuing to use conventional dietary rhetoric, highlights the complexity of how professionals navigate nutritional knowledge in practice.”

**C)popularity of LCHF diets in south africa (504) contradicts with lack of support and stopping to follow those diets mentioned in the manuscript**

**Response:**
We appreciate the reviewer’s observation regarding the apparent contradiction between the reported popularity of LCHF diets in South Africa and the lack of professional support for and continued adherence to these diets. To clarify, we have revised the paragraph to distinguish between public/media-driven popularity and actual adoption by healthcare professionals and their patients. This revision ensures that the distinction between public enthusiasm and professional skepticism is made explicit, addressing the concern raised by the reviewer.

“While LCHF diets are widely recognized in South Africa, their popularity is largely driven by media visibility and public discourse rather than strong institutional or professional endorsement. Many healthcare professionals remained cautious about formally supporting the diet, reflecting a broader global trend where media-driven interest does not always translate into clinical adoption [60]. This suggests that scepticism about LCHF is not solely a matter of questioning its health implications but rather an uncertainty about how it can be practically adopted in resource-limited settings. Some professionals also noted that hesitation toward LCHF did not come solely from healthcare providers but was also present within the support groups themselves, which were composed of community members, including patients. This reluctance within the groups stemmed from conflicting advice received from different health workers, scepticism about non-traditional diets, and broader uncertainty about the feasibility of LCHF within low-income settings [61-63].”

**D.effectiveness of LCHF, consensus guidelines on LCHF (line 74)Vs lack of scientific consensu on it (309)**

**Response:**
We appreciate the reviewer’s observation regarding the apparent contradiction between the existence of international consensus guidelines on LCHF (line 74) and the mention of a lack of scientific consensus on it (line 309). To clarify, we have revised the paragraph to distinguish between the growing international support for carbohydrate restriction in diabetes management and the remaining concerns among healthcare professionals about its long-term effects, feasibility, and sustainability. This revision ensures that both the established guidelines and the persisting concerns among practitioners are accurately represented.

“Their primarily concern centred around the high fat content of LCHF diets and whether the diet was financially sustainable for patients from low-income communities. Many professionals believed that LCHF foods were expensive, making it difficult for patients to maintain the diet long-term. Some were willing to support patients who chose to follow LCHF, but their support was often condition on closely monitoring patients for potential negative effects. While international consensus guidelines recognize carbohydrate restriction as an effective approach for managing type 2 diabetes, scepticism remains among some health professionals, particularly regarding its long-term sustainability and applicability in under-resourced settings. Some health professionals questioned whether LCHF could effectively treat metabolic disorders, largely due to ongoing controversy surrounding the diet and the lack of full scientific agreement on its long-term effects.”

**E. Venue of meeting interviewee: private venue(line 137) Vs in the health facilities(519)**

**F.Duration of contact with interviewees: one hour(137) Vs thirty minutes(141)**

**Response:**
This has been corrected for consistency. Most interviews were conducted at private venues within the participating health facilities, ensuring both privacy and accessibility and they were scheduled for 30-60 minutes allowing for flexibility depending on participant availability and depth of discussion. Please see the Participants section for the revision.

“The participants met individually with a researcher for approximately thirty minutes to an hour in a private venue within the participating health facilities, ensuring both privacy and accessibility, where they signed the informed consent form and were interviewed in English.”

**G.selection of interviwee was done from facilities that run EBSA program nutrition education (113). That means they are expected to be knowledgabe about the program.However, you have written ( line 329-330) that "majority were introduced to (EBSA) program for the first time during the interview"**

**Response:**
We clarified that although participants were drawn from facilities serving communities where EBSA had run programs, this did not guarantee their direct involvement in or awareness of the program itself. This is now explained explicitly in the Participant section.

“Although participants were selected from some facilities serving communities where EBSA had run programs, this did not guarantee their direct involvement in or awareness of the program itself. Additionally, including professionals from communities where EBSA was planning future programs allowed the study to capture prospective opinions on the program, offering insight into potential facilitators and barriers before implementation.”

**4) lack of clarity on the role of science: line 309 and 221 which similarly state that "confusing information comes from science". These two contradict with line 226 "few health professjonals made any reference to science". Does science resolve doubts or produces and ignites them? It also begs the question , if most of the experts are not refering science, why and how do they can be confused with scientific evidences on the topic?**

**Response:**
We appreciate the reviewer’s insightful observation regarding the apparent contradiction between the claim that science causes confusion (lines 309 and 221) and the statement that few professionals referenced science (line 226). To clarify, we have revised the paragraph to reflect that while science is often perceived as a source of contradictory information, most professionals were not directly engaging with primary scientific literature. Instead, their confusion appears to stem from institutional dietary guidelines, media narratives, and public health messaging rather than direct interpretation of research.

“Nurses and physicians often lacked confidence in their own nutritional knowledge and reported feeling uncertain when discussing dietary concepts. Rather than advising patients themselves, many preferred to refer them to dietitian, especially if they felt the patient required specific nutritional advice. They cited contradictory information from science, media, and government policies as key sources of confusion. Health professionals highlighted this inconsistency as a challenge for both themselves and their patients, contributing to public uncertainty about nutrition. A few health professionals attributed their lack of confidence in providing nutrition advice to insufficient training in both their formal education and continued professional development. Few participants referenced scientific evidence when justifying dietary recommendations. Therefore, while science was often cited as a source of conflicting information, it was rarely directly referenced by participants. Their confusion appeared to be from inconsistent public health messaging, institutional guidelines, and media influence rather than direct interaction with scientific literature.”

**5). Tha dat collecton (for this research) conducted in nov 2019 and the corresponding findings might not be of high relevance to adress the new dietary trends, including dietary transition initiatives.**

**Response:**
We acknowledge the reviewer’s concern regarding the relevance of findings given that data collection occurred in 2019-2020. To address this, we have added a statement in the Limitations section clarifying that the findings reflect a specific period and that dietary discourse in South Africa may have evolved, particularly in response to pandemic-related changes in food access and health priorities. This temporal framing ensures that readers interpret the findings within the appropriate context.

“This study has a temporal limitation, as data collection took place between November 2019 and February 2020, meaning the findings capture a specific moment in time. Dietary discourse in South Africa may have evolved since then, particularly due to pandemic-related shifts in food access, nutrition policies, and public health priorities. While the study provides valuable insights into health professionals' perspectives, future research is needed to explore how these views may have changed with emerging dietary trends and transition initiatives.”

**6).inaccurate targeting of the sample population. The right targets should be nutritionists/dieticians. Not only that, they also should not be from private facilities.
Generally, the paper has methodological and content gaps that need to be corrected.**

**Response:**
We acknowledge the reviewer’s concern regarding the sample selection and the inclusion of healthcare professionals beyond dietitians. While dietitians are indeed key informants in dietary counselling, our study intentionally included physicians and nurses to capture a broader perspective on how nutrition messages are conveyed within primary care. This approach aligns with community health models, which emphasize the role of generalist providers in under-resourced settings where dietitians are not always available. To address this, we have clarified this rationale we expanded the limitations section to acknowledge the potential for further specialized insights from a dietitian-focused study. This ensures that readers understand the study’s scope while recognizing opportunities for future research.

“While dietitians play a central role in dietary counselling, this study included physicians and nurses to capture how nutrition messages are communicated in primary care, aligning with community health models for under-resourced settings. Future research focusing solely on dietitians could provide more specialized insights into LCHF counselling and implementation.”

**Reviewer #4: I am grateful for the opportunity to review this study, which already has the merit of bringing to light a little-known reality and featuring authors whose backgrounds make the work international.
The study is interesting, but I notice that the interviews were conducted more than 5 years ago, and the bibliography is a lot but very dated. I suggest motivating why it took so long to publish this work and what justifies using interviews from 5 years ago. Health issues and contexts evolve very quickly.
I wonder what is new about a paper that analyses data from 5 years earlier. I would therefore suggest specifying well what this study brings that is new.**

**Response:**
We appreciate the reviewer’s concern regarding the timing of data collection and the relevance of findings given the evolving nature of health issues. The study was conducted in 2019-2020, and while there has been a time gap in publication, the findings remain relevant as they provide critical insights into healthcare professionals' perspectives on dietary counseling and LCHF diets in under-resourced settings—issues that continue to be debated globally. To address this, we have added a justification in the Discussion section noting that while dietary discourse and healthcare priorities may have shifted since data collection, the core challenges identified—such as conflicting dietary guidance, limited nutrition training, and practical barriers to implementing dietary interventions—remain persistent in South Africa’s primary care landscape. Additionally, we have acknowledged this as a study limitation while emphasizing that the findings offer a valuable historical perspective on professional attitudes toward LCHF diets at a critical moment in public health discourse.

Furthermore, to ensure that the study remains current, we have reviewed and updated the bibliography to include more recent references where applicable. We also clarify that this study brings new contributions by being one of the first to explore how a multidisciplinary group of healthcare providers, beyond just dietitians, perceive LCHF diets and community-based nutrition programs in South Africa. These insights help inform future strategies for improving dietary counseling in primary care settings, particularly in under-resourced communities.

**Furthermore, the bibliography should be updated to the last 10 years, preferably 5 years. Braun and Clarke 2006 can also be updated.**

**Response:**
Thank you for your suggestion regarding the bibliography. We have reviewed and updated several references to ensure the inclusion of recent and relevant literature. Regarding Braun and Clarke (2006), we have intentionally retained this citation as it remains the foundational reference for the type of thematic analysis conducted in our study. Although more recent work by the authors exists, including their 2022 publication, the methodological approach we used aligns specifically with the principles outlined in their 2006 paper, rather than with reflexive thematic analysis as defined in later works. We have clarified this in the manuscript to maintain consistency and methodological rigour. However, we have included Braun and Clarke (2022) in the reference list to acknowledge and address the ongoing debate around the concept of data saturation in qualitative research, as discussed in our section on sampling and data sufficiency.

We have added the following statement to clarify our approach:

“The 2006 version of Braun and Clarke’s thematic analysis was used. This version is characterised by its epistemological flexibility and openness to the use of codebooks, a feature that has since been abandoned by the authors in more recent iterations of this analytical method [45].”

**Here are some more suggestions:
Title: Specify in the title the type of research: a qualitative research**

**Response:**
We appreciate the reviewer’s suggestion to specify the research type in the title. However, we believe that the current title, “Between support and skepticism: Health professionals’ views on a nutrition education program promoting Low-Carbohydrate, High-fat diets in under-resourced South African communities” already implies a qualitative approach. The term “views” naturally indicates that the study explores perceptions and experiences, which aligns with qualitative research methodologies. Additionally, the abstract and methods section clearly define the study as a qualitative investigation, including details on in-depth interviews and thematic analysis. Given this, we feel that explicitly stating "qualitative research" in the title is unnecessary. That said, if the editorial team or reviewers strongly prefer including a methodological descriptor, we are open to refining the title to ensure clarity while maintaining its impact.

**Keywords: Redefine keywords by adding for example perceptions, diet... and changing the order of the words: 6. thematic analysis; 3 low-carbohydrate, high-fat diet; 5 South Africa; 4 under-resourced communities; 2. health professionals. 1. perspectives on nutrition**

**Response:**
We appreciate the reviewer’s suggestion to refine the keywords for better alignment with the study's focus. These adjustments improve clarity by emphasizing perspectives and perceptions, restructuring the order for better readability, and ensuring the keywords accurately reflect the study's scope. We appreciate this valuable suggestion.

Based on this feedback, we have revised the keywords as follows:

Revised Keywords:
perspectives on nutrition; healthcare professionals; low-carbohydrate, high-fat diet (LCHF); under-resourced communities; South Africa; thematic analysis; qualitative research.

**Background:
The EBSA programme is not adequately described and the relevant literature does not support the use of this diet with solid evidence. I suggest that it should be better explained and recent and specific literature on this programme should be included. If it is a new programme, it should be explained in detail.
I would also explain in more detail studies 38-39-40 that are already specifically evaluating this programme.**

**Response:**
We have added detail on EBSA’s approach and placed it within the context of broader South African dietary guidelines in the Introduction.

“Various nutritional education initiatives are active in low socio-economic areas of South Africa to address the burden of NCDs, with Eat Better South Africa (EBSA) being one prominent program [31]. EBSA promotes a low-carbohydrate high-fat (LCHF) dietary approach, focusing on nutrient-dense, minimally processed foods [31]. In contrast, the South African Food-Based Dietary Guidelines (FBDG) emphasize balanced diets, with staple whole grains, moderate fat intake, and a heavy focus on plant-based foods [32].”

We appreciate the request for further elaboration on previous evaluations of the EBSA program. We have now incorporated findings in the Introduction section from three studies that assessed the program's impact on participants' perceptions, metabolic health, and adherence challenges. These studies provide important context, demonstrating both the benefits and the practical difficulties of implementing LCHF diets in under-resourced communities. The revisions ensure that our discussion aligns with existing evidence and highlights the evolving understanding of EBSA’s role in community nutrition interventions.

“A mixed methods evaluation of the EBSA program showed significant improvements in metabolic health markers among participants after six months, including weight loss, reduced waist circumference, and improved glycemic control, although adherence remained a challenge due to socioeconomic factors [40]. Additionally, an earlier study reported that program participants felt empowered through nutritional education, but required ongoing support to sustain dietary changes [38]. A qualitative community assessment exploring women’s willingness to participate in an EBSA program highlighted both interest in and concerns about the affordability of LCHF diets, as well as challenges related to dietary transition [39]. These findings indicate that while EBSA has had measurable success in improving metabolic health, practical barriers to long-term adherence remain, necessitating better integration with healthcare professionals and community resources.”

**In this case, perhaps the research problem could be specified before the objective. For example, it could be: The results on the population of this dietetic education are positive (?), but the professionals still have different opinions. What then are the opinions of the professionals on the ESBA education programme?
The objectives of the study are different and do not facilitate consistency between objective, data collection instrument (interview) and analysis. I would propose to select only one objective, the first one, at least as a macro-objective. The others could become sub-objectives.**

**Response:**
We appreciate the reviewer’s suggestion to refine the research problem and objectives for greater clarity and consistency. We have clarified the primary objective while integrating the other objectives within the same framework to ensure alignment between the study’s aim, data collection, and analysis. These revisions strengthen the connection between the research problem, objectives, and methodological approach, ensuring a clearer focus on how healthcare professionals perceive and engage with EBSA’s dietary recommendations.

“While some health professionals may support LCHF diets for certain individuals or specific health conditions, others may have reservations based on concerns about long-term health effects, individual variability, sustainability, adherence, and potential nutrient deficiencies [41,42]. Although the EBSA program has shown positive outcomes in improving dietary behaviours and metabolic health among participants, healthcare professionals hold varying opinions on its dietary approach, highlighting the need to explore their perspectives in greater depth. This article aims to explore healthcare professionals' perspectives on the EBSA program, particularly focusing on the effectiveness and sustainability of the LCHF diet in managing NCDs within South Africa. While the primary objective is to examine how healthcare professionals perceive the effectiveness of the EBSA program and its dietary recommendations, this study also investigates the barriers and facilitators that influence their willingness to support or integrate EBSA dietary guidance into their practice. Furthermore, it assesses how professional training, healthcare system constraints, and exposure to evolving nutrition science shape the attitudes of these healthcare professionals toward LCHF diets and nutrition education. It examines the alignment and potential conflicts between the nutritional advice given by professionals and the guidelines promoted by EBSA, identifying gaps in nutrition education and training. This article also explores the broader public health implications of integrating such dietary recommendations and seeks to provide insights for refining the EBSA program to better meet the health needs of South Africa's diverse population.”

**Material and methods
Since the setting is very detailed, insert a sub-paragraph with ‘setting’. Line 116**

**Response:**

We have added a sub-paragraph titled *Setting*, which describes the context of the healthcare facilities involved in the study. This helps to better frame the environment in which data collection took place, as follows:

**Participants. As a rule, participants in the study are also given a consent form for the use of the data. With this consent, anonymised and aggregated data can be disseminated. Has a particular choice been made in this respect?**

**Response:**

We appreciate the reviewer's concern regarding participant consent for data sharing. As outlined in the Data Availability section, participants did not specifically provide consent for their transcripts to be shared publicly. The consent statement, approved by the Human Research Ethics Committee of the University of Cape Town, did not include a provision for making the data publicly available. Therefore, we do not have participant consent to share these data openly. However, requests for anonymized data can be made to the Principal Investigator, Georgina Pujol-Busquets ([georgipbg@uoc.edu](mailto:georgipbg@uoc.edu)), in accordance with ethical guidelines. This ensures that data confidentiality and participant agreements are upheld.

**Inclusion criteria: ‘a community where EBSA is planning to run future programmes’. Why?**

**Response:**

The inclusion criterion regarding “communities where EBSA plans to run future programs” was included to explore prospective opinions of healthcare professionals who may soon work with the EBSA program. This forward-looking approach provides valuable insight into how the program could be received, adapted, or challenged in the future. This has been added in the Participants section.

“Additionally, including professionals from communities where EBSA was planning future programs allowed the study to capture prospective opinions on the program, offering insight into potential facilitators and barriers before implementation.”

**Procedures
Line 142: There is the problem of the year of the interviews, but it has already been addressed in the introduction.**

**Response:**

We have clarified in the text that the interviews were conducted between November 2019 and February 2020, and that this timing is acknowledged as a limitation in understanding any shifts in dietary discourse.

“This study has a temporal limitation, as data collection took place between November 2019 and February 2020, meaning the findings capture a specific moment in time. Dietary discourse in South Africa may have evolved since then, particularly due to pandemic-related shifts in food access, nutrition policies, and public health priorities. While the study provides valuable insights into health professionals' perspectives, future research is needed to explore how these views may have changed with emerging dietary trends and transition initiatives.”

**Line 144: I have some concerns about the sample and saturation. I prefer to talk about participants, rather than sampling, which is more typical of quantitative research. With respect to data saturation, opinions are still very diverse today. While I do not agree with what has been written, I would not change it as you have cited the literature. (I would add, however, that using thematic analysis by Braun and Clarke, these authors have written several articles on data saturation, always in a reflexive manner).**

**Response:**

We appreciate the reviewer’s concern regarding the terminology of sampling in qualitative research and the debate around data saturation in reflexive thematic analysis. In response, we have adjusted the wording in the Procedures section to emphasize that our study follows qualitative research principles that prioritize depth of insight. Additionally, we have acknowledged Braun and Clarke’s position on data saturation, which views thematic development as an interpretive process rather than a point of theoretical exhaustion. This ensures that the discussion of saturation is aligned with the methodological framework used in the study.

“The IDIs were conducted between 29th of November and 21st February 2020 and lasted, on average, 30 minutes. The number of participants aligns with qualitative research recommendations, which suggest that meaningful insights can be drawn from smaller, information-rich samples [44]. According to the literature, the first five to six IDIs generally produce the majority of new data, and the majority of concepts are identified within the first 10 interviews. As such, new participants were interviewed until saturation was reached [44]. However, we acknowledge that as outlined by Braun and Clarke, the concept of data saturation is debated, with an emphasis on depth of interpretation rather than a fixed threshold for thematic exhaustion [45].

**Line 149. It would be important to explain how the interview was constructed.**

**Response:**

We appreciate the reviewer’s concern and a new paragraph has been added to the Procedures section:

“The interview guide was developed based on a review of the literature on healthcare professionals’ perceptions of dietary interventions, particularly low-carbohydrate diets. It was piloted with two health professionals (not included in the final sample) to ensure the relevance, clarity, and cultural appropriateness of the questions. The final guide followed a semi-structured format, allowing for follow-up questions and probing to explore emerging topics in depth. It covered several key areas: healthcare providers’ perceptions of their role in community health; major health concerns identified in their communities; views on non- NCDs such as obesity, diabetes, and cardiovascular disease; the type of dietary advice offered at their healthcare facilities; perspectives on the role of nutrition in chronic disease prevention and treatment; their understanding of local dietary patterns; and their awareness and impressions of the EBSA program and the LCHF diet, including views on the feasibility of implementing such interventions in their communities.”

**Line 154. Ok the deregistration with software, but as you have reviewed the transcripts, I would ask you if you have pointed out the researchers‘ field notes or if you have retained part of the participants’ paraverbal communication, useful elements for the analysis.**

**Response:**

Thank you for this insightful observation. We have now clarified in the Data Analysis section that although field notes were not formally incorporated into the coding framework, researchers actively documented observations during and immediately after each interview. These notes captured contextual and paraverbal elements—such as tone of voice, significant pauses, and emphasis—that, while not coded directly, played an important role in informing and enriching our interpretative process. This documentation supported a deeper understanding of participants’ responses and contributed to the overall reflexivity of the analysis.

“During and after the interviews, the research team systematically recorded observations and took field notes, including contextual insights and paraverbal cues such as tone, pauses, and emphasis. Although this data was not formally coded, it was used in the process of interpreting and understanding the participants’ narratives and in the development of the themes”.

**Line 159: Table 1: I would suggest moving the detailed, very thorough interview to the annex. In the text insert a summary of the interview, reworked with main subject areas and, for each area, one or two sample questions.
Response:**

Thank you for this suggestion. The full interview guide has now been moved to a supplementary appendix, and the text in the Procedures section includes a summarized version highlighting key topic areas.

“The interview guide was developed based on a review of the literature on healthcare professionals’ perceptions of dietary interventions, particularly low-carbohydrate diets. It was piloted with two health professionals (not included in the final sample) to ensure the relevance, clarity, and cultural appropriateness of the questions. The final guide followed a semi-structured format, allowing for follow-up questions and probing to explore emerging topics in depth. It covered several key areas: healthcare providers’ perceptions of their role in community health; major health concerns identified in their communities; views on NCDs such as obesity, diabetes, and cardiovascular disease; the type of dietary advice offered at their healthcare facilities; perspectives on the role of nutrition in chronic disease prevention and treatment; their understanding of local dietary patterns; and their awareness and impressions of the EBSA program and the LCHF diet, including views on the feasibility of implementing such interventions in their communities.”

**Before analysing the data, you should include which instrument was used for reporting the qualitative research. On the basis of the chosen instrument, a paragraph on the research team could be inserted, specifying the expertise in qualitative research.**

**Response:**

We appreciate the reviewer’s suggestion to specify the qualitative reporting instrument and provide information on the research team’s expertise. In response, we have stated in the Data Analysis section that the study followed the COREQ guidelines to ensure comprehensive and transparent qualitative reporting. Additionally, in the Materials and Methods section, we have included a sentence about the research team’s expertise in qualitative research and analysis, highlighting how the involvement of multiple researchers strengthened the findings.

“The study adhered to the Consolidated Criteria for Reporting Qualitative Research (COREQ) guidelines to ensure that the methods and findings were reported in a transparent and rigorous manner [48].”

Reference

48. Tong A, Sainsbury P, Craig J. Consolidated criteria for reporting qualitative research (COREQ): a 32-item checklist for interviews and focus groups. *Int J Qual Health Care.* 2007;19(6):349-357. doi:10.1093/intqhc/mzm042.

**Data Analysis
Line 165. You cite Braun and Clarke (2006) and it is correct, but these authors have also written extensively on thematic analysis in very recent years. One could update the literature.**

**Response:**

Thank you for your suggestion. As we have explained in an earlier response, we followed the foundational guidance provided by Braun and Clarke (2006) in our analysis, which is more aligned with the procedures implemented in this study than their recent contributions (i.e., use of a collaboratively developed codebook and independent double coding), while still being a widely cited and relevant approach for conducting thematic analysis. We have clarified this in the manuscript to ensure methodological consistency and transparency.

“The 2006 version of Braun and Clarke’s thematic analysis was used. This version is characterised by its epistemological flexibility and openness to the use of codebooks, a feature that has since been abandoned by the authors in more recent iterations of this analytical method [45].”

**Line 169. The provision of codebooks for qualitative research is very good. I would suggest placing the basic data from which the analysis is developed in a repository linked to the article.**

**Response:**

Thank you for this helpful suggestion. As noted in the Data Availability section, participant consent did not include permission to share transcripts or raw data publicly. In accordance with the confidentiality agreements approved by the Human Research Ethics Committee of the University of Cape Town, we are therefore unable to make the basic data available in a public repository.

Regarding the use of a codebook approach, while we appreciate that its inclusion supports transparency, we note that Braun and Clarke (2022) have more recently distanced themselves from the use of codebooks in reflexive thematic analysis. They argue that the use of rigid codebooks can be at odds with the flexible, evolving nature of coding in reflexive approaches. That said, for the sake of transparency and clarity, we are open to providing a descriptive and anonymized version of the codes developed during analysis upon reasonable request.

**Line 179. Thematic analysis (by Braun &Clarke) is developed into themes and sub-themes. The transition to concepts is more titpical of grounded theory. I would relate the description of the analysis to your chosen model (themes and sub-themes)
The description of the analysis is very detailed and offers many insights into the rigour of the research. However, I would suggest pointing out a reference author with a citation for rigour (by including it as a citation in the analysis) or make a separate paragraph pointing out which dimensions of rigour you specifically considered (with reference author).**

**Response:**

Thank you for your thoughtful feedback. We have revised the Methods section substantially to clarify our analytical approach and ensure alignment with the model we used. Specifically, we have described our process as a codebook approach to thematic analysis guided by Braun and Clarke’s original 2006 framework, acknowledging that our approach does not follow the reflexive thematic analysis approach outlined in their recent publications. Accordingly, we now refer consistently to themes and sub-themes rather than "concepts," to remain methodologically consistent with thematic analysis rather than grounded theory.

In addition, we have expanded the description of how rigour was ensured throughout the study. Rather than referring to inductive analysis as a marker of rigour, we have added appropriate citations to support this section. We have clarified that we used the COREQ checklist [48] to strengthen reporting transparency and added references accordingly—see references 45, 47, and 48.

References:

45. Braun V, Clarke V. Thematic analysis: A practical guide. London: SAGE Publications; 2022.

47. Bloor M. Techniques of validation in qualitative research: a critical commentary. In: Miller G, Dingwall R, editors. Context and method in qualitative research. London: SAGE Publications; 1997. p. 37–50.

48. Tong A, Sainsbury P, Craig J. Consolidated criteria for reporting qualitative research (COREQ): a 32-item checklist for interviews and focus groups. Int J Qual Health Care. 2007;19(6):349-357. doi:10.1093/intqhc/mzm042.

**Results
Line 199. I would enter the total number of respondents in the introduction of the results. I would then imagine that a code, e.g. numeric, was assigned to each participant, while maintaining the difference in profession ( Cod. Ph. 1 to indicate the first code, e.g. Physician)**

**Response:**
We agree with this suggestion and have revised the Results introduction. This coding system is now used consistently throughout the Results section to label quotations.
"A total of 16 healthcare professionals participated in the study, including 7 medical physicians, 7 nurses, and 2 dietitians. To ensure transparency and traceability in the reporting of quotes, each participant was assigned a code reflecting their profession and participant number (e.g., MPh.1 for Male Physician 1, FN.1 for Female Nurse 1, FD.1 for Female Dietitian 1).”

**I would rework the table of themes, keeping the 4 main themes, but also inserting the sub-themes (which can be derived from the definitions) for each theme. The quotes are too many and too long (they should be an example), so I suggest selecting them and reducing them in length. For example.
Themes Sub-themes quotations
1. Foundations of dietary understanding
1.1. knowledge of nutritional guidelines, assessment.. (e.g.) ‘We basically follow the protocols of the National Health Department and (..) there isn’t really much with regards to prevention, they have information, but it’s outdated (..) (Male physician).
1.2. dietary counselling and nutrition education
Response:**

We have revised the Table 1 of themes to include sub-themes derived directly from the analysis. We have also carefully reviewed the quotes used in the Table 1 of the Results section, and have selected only the most illustrative and impactful examples for each theme. Each quote is short excerpt that conveys the core insight, while ensuring the participant’s profession and code are attached.

**Text of Results
The text of the results could also be reworked with themes and sub-themes. It would make comprehension more immediate, in my opinion.
I really appreciate it when the codes are included in the text in full or as parts of the quotes (e.g. lines 247-249, 252..). I would however add the code.**

**Response:**
We appreciate the reviewer’s suggestion to present the results with themes and sub-themes more explicitly for clarity. To address this, we have Table 1 that clearly outlines themes, sub-themes, definitions and illustrative quotes, allowing for a structured presentation of findings. However, in the Results section, we have chosen to present the findings at the theme level to maintain a coherent narrative flow and avoid excessive fragmentation of the discussion. This approach balances structured thematic presentation with a readable and engaging results section, ensuring that key findings remain accessible.

**Line 287. ‘For those who did not know about the diet..’ to remedy this, the fact of knowing about this diet could be included in the inclusion criteria.
Response:**
We appreciate the reviewer’s suggestion to include prior knowledge of the LCHF diet as part of the inclusion criteria. However, our inclusion criteria focused on selecting healthcare professionals working in communities where EBSA had implemented or planned to implement its nutrition education program, rather than requiring familiarity with the LCHF diet itself. This approach allowed us to capture a diverse range of perspectives, including those of professionals unfamiliar with LCHF, providing insights into both supportive and skeptical viewpoints. Additionally, as noted in the Participants section, while participants were selected from facilities in relevant communities, this did not guarantee their direct involvement in or awareness of the EBSA program or its dietary recommendations. Including professionals from communities where EBSA is planning future programs also allowed us to explore prospective opinions on the program’s feasibility and acceptance. This variation in awareness and familiarity with LCHF is an important part of our findings, highlighting the challenges in knowledge dissemination and the need for further professional education on different dietary approaches. Therefore, we believe maintaining the existing inclusion criteria was appropriate for the study’s objectives.

**Line 389. I would not include citations in the results**

**Response:**
Thank you for your valuable feedback. We have carefully reviewed the Results section and have rewritten Theme 4 to ensure that it is solely based on participant insights, removing references 49-52. Any necessary references from this section have been relocated to the Discussion where they provide additional context to our findings. This revision enhances the clarity and integrity of the results by ensuring that all statements are directly derived from the qualitative data. We appreciate this insightful suggestion and believe it strengthens the manuscript.

**The results, which are very extensive, go far beyond the professionals' view of the ESBA education programme, which is little or not at all known to the professionals interviewed. The various problems highlighted in the results concern the ESBA programme only in part, because they then range over the whole aspect of nutrition education, in poor populations for whom the food problem is not the priority, the professionals‘ lack of knowledge, their need for training, and above all the professionals’ doubts as to whether or not this programme is effective and how to do effective nutrition education, and the role of women in African communities. Even the title, with these results, is no longer explanatory. I therefore suggest that we seek consistency between the title, objectives and results, selecting results that express clarity on what is achieved with this study. It seems to me that the main underlining feature at the moment is the confusion of the professionals as to which programme to focus on and the request to be informed about nutrition education so as to be of help to the population**. **The conclusions follow from what has been said for the results.**

**Response:**
Thank you for your thoughtful feedback. In response to your comments and another reviewers’ ones, we have revised the title and rewritten the objectives to ensure greater consistency between the study’s aims and its findings. The updated objectives now better reflect the broader themes that emerged from the interviews, including health professionals' confusion about nutrition education, their need for further training, and their uncertainty about the effectiveness of the EBSA program within the healthcare system.

Additionally, we have refined a lot of the Results section to ensure that it remains focused on the study’s key objectives while still addressing the broader context of nutrition education in under-resourced settings. By clarifying the primary research aim, exploring healthcare professionals' perspectives on EBSA and their knowledge gaps in nutrition education, we ensure that the findings align with the study’s overall purpose.

We appreciate this insightful recommendation, as it has helped us strengthen the manuscript’s coherence and focus.

The revised title is:
*“Between support and skepticism: Health professionals’ perceptions on a nutrition education program promoting Low-Carbohydrate, High-fat diets in under-resourced South African communities.”*

The Conclusion has also been rewritten to focus explicitly on the study’s contributions, see the revised section:

“This study provides valuable insights into healthcare professionals’ perspectives on a nutrition education program recommending LCHF diets, highlighting key challenges in their understanding and implementation within under-resourced South African communities. Professionals expressed uncertainty around LCHF, often shaped by conflicting guidelines, limited exposure to recent evidence, and scepticism about its long-term effects. The findings also reveal significant training gaps in nutrition education, with many professionals, mostly doctors and nurses, lacking confidence in providing dietary guidance beyond conventional recommendations. Additionally, socioeconomic barriers such as food affordability, cultural norms, and structural inequalities further complicate dietary behaviour change in these communities. Despite these challenges, according to participants, EBSA presents a promising model for integrating nutrition education into healthcare strategies, particularly by offering structured support and practical guidance tailored to local contexts. Future efforts should focus on bridging knowledge gaps, strengthening professional training, and ensuring that dietary interventions are both evidence-based and accessible to those who need them most.”

**6. PLOS authors have the option to publish the peer review history of their article (what does this mean?<**[**https://track.editorialmanager.com/CL0/https:%2F%2Fjournals.plos.org%2Fplosone%2Fs%2Feditorial-and-peer-review-process%23loc-peer-review-history/1/010f019547d21ab6-08e7bc50-c91e-4e43-a149-14affdf0e8f0-000000/XI4asZMiNT-0EPVAjO5moDp19tutEvwUtzZofPwF3tU=200**](https://track.editorialmanager.com/CL0/https:%2F%2Fjournals.plos.org%2Fplosone%2Fs%2Feditorial-and-peer-review-process%23loc-peer-review-history/1/010f019547d21ab6-08e7bc50-c91e-4e43-a149-14affdf0e8f0-000000/XI4asZMiNT-0EPVAjO5moDp19tutEvwUtzZofPwF3tU=200)**>). If published, this will include your full peer review and any attached files.
If you choose “no”, your identity will remain anonymous but your review may still be made public.
Do you want your identity to be public for this peer review? For information about this choice, including consent withdrawal, please see our Privacy Policy<**[**https://track.editorialmanager.com/CL0/https:%2F%2Fwww.plos.org%2Fprivacy-policy/1/010f019547d21ab6-08e7bc50-c91e-4e43-a149-14affdf0e8f0-000000/RHicORkQCDHbpzx7TsW5gop56rrq6WXp5tKo5ZFwpc0=200**](https://track.editorialmanager.com/CL0/https:%2F%2Fwww.plos.org%2Fprivacy-policy/1/010f019547d21ab6-08e7bc50-c91e-4e43-a149-14affdf0e8f0-000000/RHicORkQCDHbpzx7TsW5gop56rrq6WXp5tKo5ZFwpc0=200)**>.

Reviewer #1: No

Reviewer #2: No

Reviewer #3: Yes: Abdelah Alifnur Mohammed

Reviewer #4: No

[NOTE: If reviewer comments were submitted as an attachment file, they will be attached to this email and accessible via the submission site. Please log into your account, locate the manuscript record, and check for the action link "View Attachments". If this link does not appear, there are no attachment files.]
While revising your submission, please upload your figure files to the Preflight Analysis and Conversion Engine (PACE) digital diagnostic tool,** [**https://pacev2.apexcovantage.com/**](https://pacev2.apexcovantage.com/)**<**[**https://track.editorialmanager.com/CL0/https:%2F%2Fpacev2.apexcovantage.com%2F/1/010f019547d21ab6-08e7bc50-c91e-4e43-a149-14affdf0e8f0-000000/qIu5THaYeTAR8UHYhmKIhKp-Eqwi3kurqp3yQi67OPM=200**](https://track.editorialmanager.com/CL0/https:%2F%2Fpacev2.apexcovantage.com%2F/1/010f019547d21ab6-08e7bc50-c91e-4e43-a149-14affdf0e8f0-000000/qIu5THaYeTAR8UHYhmKIhKp-Eqwi3kurqp3yQi67OPM=200)**>. PACE helps ensure that figures meet PLOS requirements. To use PACE, you must first register as a user. Registration is free. Then, login and navigate to the UPLOAD tab, where you will find detailed instructions on how to use the tool. If you encounter any issues or have any questions when using PACE, please email PLOS at** [**figures@plos.org**](mailto:figures@plos.org)**<mailto:**[**figures@plos.org**](mailto:figures@plos.org)**>. Please note that Supporting Information files do not need this step.
In compliance with data protection regulations, you may request that we remove your personal registration details at any time. (Remove my information/details)<**[**https://track.editorialmanager.com/CL0/https:%2F%2Fwww.editorialmanager.com%2Fpone%2Flogin.asp%3Fa=r/1/010f019547d21ab6-08e7bc50-c91e-4e43-a149-14affdf0e8f0-000000/uRQRV5v6A1j4LvS-xuljxaCPXWqS-wEDnsuXRxkgpYo=200**](https://track.editorialmanager.com/CL0/https:%2F%2Fwww.editorialmanager.com%2Fpone%2Flogin.asp%3Fa=r/1/010f019547d21ab6-08e7bc50-c91e-4e43-a149-14affdf0e8f0-000000/uRQRV5v6A1j4LvS-xuljxaCPXWqS-wEDnsuXRxkgpYo=200)**>. Please contact the publication office if you have any questions.[**[**http://track.editorialmanager.com/CI0/010f019547d21ab6-08e7bc50-c91e-4e43-a149-14affdf0e8f0-000000/wujjvCxXJxP7JYvFv3lar32WD_R8z_Q0gRxkc0axlZk=200**](http://track.editorialmanager.com/CI0/010f019547d21ab6-08e7bc50-c91e-4e43-a149-14affdf0e8f0-000000/wujjvCxXJxP7JYvFv3lar32WD_R8z_Q0gRxkc0axlZk=200)**]**
